# Supplementary material for: Adolescents’ reasons for accessing their health records online, perceived usefulness and experienced provider encouragement: a national survey in Sweden
Source: BMJ Paediatr Open. 2024 Mar 9;8(1):e002258. doi: 10.1136/bmjpo-2023-002258 (PMC10928755; doi:10.1136/bmjpo-2023-002258)
Supplement: Supplementary data [file bmjpo-2023-002258supp001.pdf]

Appendix A

Table 1. Original survey created in English

Number in survey	Question	Response alternatives
6	**How often do you think that you have accessed your health record during the last 12 months?**	1 - This is my first time<br>2 - 2 to 9 times<br>3 - 10 to 20 times<br>4 - More than 20 times
7	**Did any of the following encourage or remind you to read your health record?**	HCPs: Physician<br>HCPs: Nurse<br>HCPs: Psychologist<br>HCPs: Physiotherapist<br>HCPs: Other medical staff<br>Written information provided by the hospital/healthcare facility /clinic<br>Family or friends<br>Web-pages, such as the National health portal web-page, etc.<br>Newspapers, radio, TV, Facebook, etc.<br>Other patients<br>Nobody encouraged me<br>Other
8	Please indicate how much you disagree or agree with the following statement:<br>**I read my health record online…**	1 - Disagree,<br>2, 3, 4,<br>5 - Agree
	out of general curiosity	
	to improve my understanding about my health issue	
	to prepare myself for a consultation or hospitalization	
	to get an overview of my medical history and/or treatment	
	to be sure I understood what the physician/healthcare professional said	
	to remember the care plan/follow my treatment recommendations	
	because I suspect inaccuracies	
	to share documents with relatives	
	to share documents with friends	
	to share documents with health professionals who do not have access	
	because I am not sure if I got the right care	
	Other, please specify	

38	**How useful would it be to have access to the following information in the portal?**		1 - Not useful at all<br>2 - Not very useful<br>3 - Neither useful nor useless<br>4 - Useful<br>5 - Very useful
		The core/summary record with critical information	
		Referrals (content and how it is handled in care)	
		List of all pharmaceuticals/my medications	
		Overview of vaccinations	
		Results of tests	
		Clinical notes from primary care	
		Clinical notes from hospital care	
		Overview of all health care contacts	
39	**How useful would it be to have access to the following functions in the portal?**		1 - Not useful at all<br>2 - Not very useful<br>3 - Neither useful nor useless<br>4 - Useful<br>5 - Very useful
		Ability to access information and manage services for my children	
		Ability to access information and manage services for my family members	
		See which care units and staff groups have accessed my information (see log data)	
		Ability to block certain medical records from access by other medical staff	
		Ability to contact health care provider electronically and ask questions about medical record	
		Ability to point out errors I find in the health record	
		Ability to write own comments to text in the health record	
		Contribute with information on health, for example by providing a health declaration for next visit	
		Contribute with information of self-testing/monitoring at home	
		Contribute information about expectations for the health care visit	
		Ability to order and manage medical certificate and other certificates	
		Other	

Table B2. Swedish translation of survey

Number in survey	Question	Response alternatives
6	**Hur ofta har du läst i din journal under de senaste 12 månaderna?**	1 - Det här är första gången<br>2 - 2-9 gånger<br>3 - 10-20 gånger<br>4 - Mer än 20 gånger
7	**Har du blivit uppmuntrad eller påmind att läsa din journal av någon/något av följande:**	Vårdpersonal: Läkare<br>Vårdpersonal: Sjuksköterska<br>Vårdpersonal: Psykolog<br>Vårdpersonal: Fysioterapeut/sjukgymnast<br>Vårdpersonal: Annan vårdpersonal<br>Skriftlig information från sjukhuset/vårdinrättning/klinik<br>Familj eller vänner<br>Webbsidor, såsom 1177.se Vårdguiden, etc.<br>Tidningar, radio, TV, Facebook, etc.<br>Andra patienter<br>Ingen har uppmuntrat eller påmint mig<br>Annat
8	Markera i vilken utsträckning du instämmer med följande påståenden:<br>**Jag läser min journal på internet…**	1 - Instämmer inte, 2, 3, 4,<br>5 - Instämmer
	Av nyfikenhet	
	För att förbättra min förståelse för mitt hälsotillstånd	
	För att förbereda mig själv för ett vårdbesök eller sjukhusvistelse	
	För att få en överblick av min hälsohistoria och/eller bakgrund	
	För att försäkra mig om att jag förstått vad läkaren/vårdpersonalen sagt	
	För att komma ihåg vårdplanen/följa mina behandlingsrekommendationer	
	För att jag misstänker felaktigheter	
	För att dela med mig av information till släkt	
	För att dela med mig av information till vänner	
	För att dela med mig av information till vårdpersonal som inte har tillgång till min journal	
	För att jag är osäker på om jag fått rätt vård	
	Annat, specificera gärna	

38	**Hur användbart skulle det vara för dig att ha tillgång till följande information i *Journalen*?**		1 - Inte alls användbart<br>2 - Inte särskilt användbart<br>3 - Varken eller<br>4 - Användbart<br>5 - Väldigt användbart
		En sammanfattning av journalen med viktig information	
		Remisser (innehåll och status)	
		Lista över mina läkemedel/mediciner	
		Vaccinationer	
		Provsvar	
		Journalanteckningar från primärvården	
		Journalanteckningar från specialistvården	
		Överblick av samtliga kontakter med vården	
39	**Hur användbart skulle det vara för dig att ha tillgång till följande funktioner i *Journalen*?**		1 - Inte alls användbart<br>2 - Inte särskilt användbart<br>3 - Varken eller<br>4 - Användbart<br>5 - Väldigt användbart
		Möjlighet att ta del av information och hantera tjänster för mina barn	
		Möjlighet att ta del av information och hantera tjänster för andra familjemedlemmar	
		Möjlighet att kunna blockera specifika journalanteckningar för viss vårdpersonal	
		Se vilka vårdinrättningar och personal som har läst i min journal (loggar)	
		Möjlighet att kunna kontakta vårdpersonal online för att ställa frågor om min journal	
		Möjlighet att påpeka felaktigheter i journalen	
		Möjlighet att skriva egna kommentarer i journalen	
		Möjlighet att bidra med information om min hälsa, t ex genom att fylla i en hälsodeklaration, inför ett besök	
		Möjlighet att bidra med information från självtest/monitorering hemma	
		Möjlighet att bidra med information om mina förväntningar på vårdbesöket	
		Möjlighet att beställa och hantera sjukskrivning, medicinsk intyg och andra dokument (vaccinationsintyg t ex)	
		Annat	
