# Supplementary material for: Adolescents’ reasons for accessing their health records online, perceived usefulness and experienced provider encouragement: a national survey in Sweden
Source: BMJ Paediatr Open. 2024 Mar 9;8(1):e002258. doi: 10.1136/bmjpo-2023-002258 (PMC10928755; doi:10.1136/bmjpo-2023-002258)
Supplement: Supplementary data [file bmjpo-2023-002258supp002.pdf]

Appendix B

Detailed results from the survey questions chosen for this study.

Encouragement to Read.

**Did any of the following encourage or remind you to read your health record? n (%)**	**Frequency**
HCPs: Physician	24 (11.0)
HCPs: Nurse	26 (11.9)
HCPs: Psychologist	14 (6.4)
HCPs: Physiotherapist	6 (2.8)
HCPs: Other medical staff	9 (4.1)
Written information provided by the hospital/healthcare facility/clinic	12 (5.5)
Family or friends	63 (28.9)
Web-pages, such as the National health portal web-page, etc.	29 (13.3)
Newspapers, radio, TV, Facebook, etc.	1 (0.5)
Other patients	5 (2.3)
Nobody encouraged me	118 (54.1)
Other	4 (1.8)
**Encouraged by HCP or not, n (%)**	
Encouraged by HCP	47 (21.6)
Not encouraged by HCP	171 (78.4)

Reasons for reading health records online.

**I read my health record online…, n** (%)	**1. Disagree**	**2**	**3**	**4**	**5. Agree**
… out of general curiosity	9 (4.1)	10 (4.6)	29 (13.3)	35 (16.1)	135 (61.9)
… to improve my understanding about my health issue	16 (7.3)	22 (10.1)	42 (19.3)	49 (22.5)	89 (40.8)
… to prepare myself for a consultation or hospitalization	41 (18.8)	37 (17.0)	44 (20.2)	36 (16.5)	60 (27.5)
… to get an overview of my medical history and/or treatment	8 (3.7)	14 (6.4)	37 (17.0)	55 (25.2)	104 (47.7)
… to be sure I understood what the physician/healthcare professional said	20 (9.2)	19 (8.7)	30 (13.8)	50 (22.9)	99 (45.4)
… to remember the care plan/follow my treatment recommendations	49 (22.5)	32 (14.7)	36 (16.5)	37 (17.0)	64 (29.4)
… because I suspect inaccuracies	113 (51.8)	35 (16.1)	31 (14.2)	25 (11.5)	14 (6.4)
… to share documents with relatives	115 (52.8)	41 (18.8)	31 (14.2)	14 (6.4)	17 (7.8)
… to share documents with friends	122 (56.0)	38 (17.4)	31 (14.2)	14 (6.4)	13 (6.0)
… to share documents with healthcare professionals who do not have access	146 (67.0)	15 (6.9)	28 (12.8)	13 (6.0)	16 (7.3)
… because I am not sure if I got the right care	132 (60.6)	22 (10.1)	23 (10.6)	20 (9.2)	21 (9.6)
Other	168 (77.1)	20 (9.2)	5 (2.3)	3 (1.4)	22 (10.1)

Perceived usefulness of various types of information.

**Information**	**Not useful at all**	**Not very useful**	**Neither useful nor useless**	**Useful**	**Very useful**
The core/summary record with critical information	1 (0.5)	8 (3.7)	28 (12.8)	83 (38.1)	98 (45.0)
Referrals (content and how it is handled in care)	1 (0.5)	3 (1.4)	26 (11.9)	63 (28.9)	125 (57.3)
List of all pharmaceuticals / my medications	0 (0)	11 (5.0)	13 (6.0)	43 (19.7)	151 (69.3)
Overview of vaccinations	5 (2.3)	8 (3.7)	21 (9.6)	67 (30.7)	117 (53.7)
Results of tests (not available in all regions)	2 (0.9)	3 (1.4)	8 (3.7)	49 (22.5)	156 (71.6)
Clinical notes from primary care	0 (0)	1 (0.5)	24 (11.0)	61 (28.0)	132 (60.6)
Clinical notes from hospital care	0 (0)	2 (0.9)	18 (8.3)	63 (28.9)	135 (61.9)
Overview of all health care contacts	1 (0.5)	5 (2.3)	43 (19.7)	60 (27.5)	109 (50)

Perceived usefulness of various functions.

Function	Not useful at all	Not very useful	Neither useful nor useless	Useful	Very useful
Ability to access information and manage services for my children (not available in all regions).	31 (14.2)	9 (4.1)	73 (33.5)	46 (21.1)	59 (27.1)
Ability to access information and manage services for my family members.	46 (21.1)	30 (13.8)	83 (38.1)	39 (17.9)	20 (9.2)
Ability to block specific clinical notes from certain healthcare professionals/medical staff.	31 (14.2)	33 (15.1)	80 (36.7)	40 (18.3)	34 (15.6)
See which care units and staff groups have accessed my information (see log data).	10 (4.6)	8 (3.7)	43 (19.7)	77 (35.3)	80 (36.7)
Ability to contact health care provider electronically and ask questions about medical record.	3 (1.4)	10 (4.6)	26 (11.9)	90 (41.3)	89 (40.8)
Ability to point out errors I find in the health record.	9 (4.1)	7 (3.2)	33 (15.1)	90 (41.3)	79 (36.2)
Ability to write own comments to text in the health record.	19 (8.7)	26 (11.9)	48 (22.0)	70 (32.1)	55 (25.2)
Contribute with information on health, for example by providing a health declaration for next visit.	3 (1.4)	9 (4.1)	32 (14.7)	90 (41.3)	84 (38.5)
Contribute with information of self-testing/monitoring at home.	13 (6.0)	12 (5.5)	65 (29.8)	74 (33.9)	54 (24.8)
Contribute information about expectations for the health care visit.	22 (10.1)	26 (11.9)	80 (36.7)	51 (23.4)	39 (17.9)
Ability to order and manage medical certificate and other certificates.	7 (3.2)	4 (1.8)	36 (16.5)	65 (29.8)	106 (48.6)
