# Supplementary material for: Adolescents’ reasons for accessing their health records online, perceived usefulness and experienced provider encouragement: a national survey in Sweden
Source: BMJ Paediatr Open. 2024 Mar 9;8(1):e002258. doi: 10.1136/bmjpo-2023-002258 (PMC10928755; doi:10.1136/bmjpo-2023-002258)
Supplement: Author's manuscript [file bmjpo-2023-002258.draft_revisions.pdf]

BMJ Paediatrics Open

BMJ Paediatrics Open is committed to open peer review. As part of this commitment we make the peer review history of every article we publish publicly available.

When an article is published we post the peer reviewers' comments and the authors' responses online. We also post the versions of the paper that were used during peer review. These are the versions that the peer review comments apply to.

The versions of the paper that follow are the versions that were submitted during the peer review process. They are not the versions of record or the final published versions. They should not be cited or distributed as the published version of this manuscript.

BMJ Paediatrics Open is an open access journal and the full, final, typeset and author-corrected version of record of the manuscript is available on our site with no access controls, subscription charges or pay-per-view fees (http://bmjpaedsopen.bmj.com).

If you have any questions on BMJ Paediatrics Open's open peer review process please email info.bmjpo@bmj.com

BMJ Paediatrics Open

Views and experiences of adolescents reading their health records online: Results from a national survey

Journal:	*BMJ Paediatrics Open*
Manuscript ID	bmjpo-2023-002258
Article Type:	Original research
Date Submitted by the Author:	30-Aug-2023

Complete List of Authors:	Hagström, Josefin; Uppsala Universitet, Department of Women's and Children's Health<br>Blease, Charlotte; Uppsala Universitet, Department of Women's and Children's Health; Harvard Medical School, Dept of Psychiatry, Beth Israel Deaconess Medical Center<br>Scandurra, Isabella; Örebro University, Informatics, School of Business<br>Moll, Jonas; Örebro University, Informatics, School of Business<br>Cajander, Åsa; Uppsala Universitet, Department of Information Technology<br>Rexhepi, Hanife; University of Skövde, School of Informatics<br>Hägglund, Maria; Uppsala Universitet, Department of Women's and Children's Health; Uppsala University Hospital

Keywords:	Adolescent Health, Health services research, Data Collection

SCHOLARONE™
Manuscripts

I, the Submitting Author has the right to grant and does grant on behalf of all authors of the Work (as defined in the below author licence), an exclusive licence and/or a non-exclusive licence for contributions from authors who are: i) UK Crown employees; ii) where BMJ has agreed a CC-BY licence shall apply, and/or iii) in accordance with the terms applicable for US Federal Government officers or employees acting as part of their official duties; on a worldwide, perpetual, irrevocable, royalty-free basis to BMJ Publishing Group Ltd ("BMJ") its licensees and where the relevant Journal is co-owned by BMJ to the co-owners of the Journal, to publish the Work in this journal and any other BMJ products and to exploit all rights, as set out in our licence.

The Submitting Author accepts and understands that any supply made under these terms is made by BMJ to the Submitting Author unless you are acting as an employee on behalf of your employer or a postgraduate student of an affiliated institution which is paying any applicable article publishing charge ("APC") for Open Access articles. Where the Submitting Author wishes to make the Work available on an Open Access basis (and intends to pay the relevant APC), the terms of reuse of such Open Access shall be governed by a Creative Commons licence – details of these licences and which Creative Commons licence will apply to this Work are set out in our licence referred to above.

Other than as permitted in any relevant BMJ Author's Self Archiving Policies, I confirm this Work has not been accepted for publication elsewhere, is not being considered for publication elsewhere and does not duplicate material already published. I confirm all authors consent to publication of this Work and authorise the granting of this licence.

Confidential: For Review Only

TITLE PAGE

Article title: Views and experiences of adolescents reading their health records online: Results from a national survey

Author information:
Corresponding author:
Josefin Hagström, MSc (ORCID: 0000-0003-2835-0259)
Participatory eHealth and Health Data Research Group, Department of Women's and Children's Health, Uppsala University, Uppsala, Sweden

josefin.hagstrom@kbh.uu.se
+46184715080

Co-authors:
Charlotte Blease PhD[1,2] (ORCID: 0000-0002-0205-1165)
Isabella Scandurra PhD[3] (ORCID: 0000-0002-2597-1079)
Jonas Moll PhD[3] (ORCID: 0000-0002-4772-4730)
Åsa Cajander PhD[4] (ORCID: 0000-0001-7472-2215)
Hanife Rexhepi PhD[5] (ORCID: 0000-0001-8957-9853)
Maria Hägglund PhD[1, 6] (ORCID: 0000-0002-6839-3651)

[1] Participatory eHealth and Health Data Research Group, Department of Women's and Children's Health, Uppsala University, Uppsala, Sweden
[2] Digital Psychiatry, Dept of Psychiatry, Beth Israel Deaconess Medical Center, Harvard Medical School, Boston, MA, USA
[3] Centre for Empirical Research on Information Systems (CERIS), Informatics, School of Business, Örebro University, Örebro, Sweden
[4] Department of Information Technology, Uppsala University, Uppsala, Sweden
[5] School of Informatics, University of Skövde, Sweden
[6] Uppsala University Hospital

Disclaimers: The views expressed in the submitted article are his or her own and not an official position of the institution or funder.

Source(s) of support: This work was supported by NordForsk through the funding to Nordic eHealth for Patients: Benchmarking and Developing for the Future, NORDeHEALTH, (Project #100477), the Swedish Research Council for Health, Working Life and Welfare (FORTE) through the funding to Beyond Implementation of eHealth (Project #2020-01229).

Word count: 2429

Number of figures: 7

Number of tables: 2

Key words: adolescents; adolescent health; Patient Accessible Electronic Health Record (PAEHR); Electronic Health Record (EHR); patient portal; national survey; ehealth

References: 37

Disclosure of relationships and activities: There are no conflicts of interest to declare.

Confidential: For Review Only

ABSTRACT

Background: Having online access to electronic health records (EHRs) may help patients become engaged in their care at an early age. However, little is known about adolescents using patient portals. A national survey conducted within the Nordic eHealth project NORDeHEALTH provided an important opportunity to advance our understanding of adolescent users of patient portals. The present study explored reasons for reading the EHRs, the perceived usefulness of information and functions in a patient portal, and the association between frequency of use and encouragement to read the EHR.
Methods: Data were collected in a survey using convenience sampling, available through the Swedish online health portal during three weeks in January and February 2022. This study included a subset of items and only respondents aged 15-19. Demographic factors and frequencies on Likert-style questions were reported with descriptive statistics, while Fisher's exact test was used to explore differences in use frequency based on source of encouragement.
Results: Of 13 008 users who completed the survey, 218 (1.68%) were unique users aged 15-19 (females: 77.1%). One fifth (47/218, 21.6%) had been encouraged by HCPs to read their records, and higher use frequency was related to being encouraged by physicians (p=.003) and nurses (p=.031). All types of information were rated high on usefulness, while some functions were rated low, such as blocking specific clinical notes from HCPs and managing services for family members. The main reason for reading their health records online was out of curiosity.
Conclusions: Adolescents who read their records online perceive it to be useful. Encouragement by HCPs can lead to increased use of patient portals among adolescents, and particularly physicians' and nurses' encouragement need further investigation. Findings should be considered in the future design of patient portals for adolescents.

Keywords: adolescents; adolescent health; Patient Accessible Electronic Health Record (PAEHR); Electronic Health Record (EHR); patient portal; national survey; ehealth

Confidential: For Review Only

KEY MESSAGES

What is already known on this topic

- Implementation of online record access for adolescents is controversial
- Citizens in Sweden have had online record access via a national patient portal for a decade
- Prior research has focused on adolescents without experience of using a patient portal, while existing studies of adolescent patient portal users have been limited to contexts of serious illness.
- Adolescents report similar benefits of reading EHRs to those of adults, such as reduced anxiety, enhanced knowledge about their illness, ability to ask informed questions and reflecting more on their health

What this study adds

- Adolescents are rarely encouraged by HCPs to read their notes online. Encouragement by physicians and nurses was related to higher use frequency.
- Adolescents perceive all types of information in the EHR as useful.

How this study might affect research, practice or policy

- Enhanced encouragement and support for adolescents but also educating HCPs on access to EHRs will be required to support the transition into adulthood and involve adolescents in their own care.
- EHR vendors designing patient portals and patient portals intended for adolescent use in particular may benefit from considering the types of information and functions that adolescents find useful.

INTRODUCTION

An increasing number of healthcare providers are giving patients access to their electronic health records (EHR), including patient information such as clinical notes, diagnoses, and medication lists. The practice of enabling access to free-text notes written in the EHRs by healthcare professionals (HCPs) is sometimes called ‘open notes’.[1] Commonly, patients gain online record access (ORA) via secure online portals. Patient-accessible electronic health records (PAEHR) are becoming increasingly expected among patients, even affecting patients’ choice of healthcare provider. In particular, adolescents are growing up with expectations of digital convenience that also applies to healthcare.[2]

During early childhood, PAEHR portal systems can often allow for parental proxy access, where parents or guardians of the minor have access to the EHR of the child. However, adolescents needing healthcare face a hidden battle in balancing privacy and personal autonomy with the need for support from parents. Worries about sensitive health information (e.g. mental health issues and sexual health) being exposed to parents through the PAEHR can dissuade young people from accessing medical care.[3,4] The transition from adolescent to adult patienthood has therefore raised concerns among HCPs[5–7] and implementing online access to EHRs is especially controversial in this population.

The national e-service for PAEHRs in Sweden is Journalen, available via the web patient portal 1177.se (Vårdguiden). According to publicly available statistics,8 Journalen is being accessed by a steadily increasing number of users; approximately 1 360 000 unique users per month during 2022. Adult parent users have automatic access to their child’s records in Journalen, but lose this access when the child turns 13, and adolescents gain their record access at 16. When an adolescent is between 13 and 16 years old, parents and adolescents can apply for extended access outside of the default. Adolescent patients access the same information as adults. The content includes for example a list of prescribed medications, lab results, growth curves, vaccinations, diagnoses, maternity care records, referrals, warnings, and treatment plans (Figure 1). However, availability differs according to region and connected health care providers (who have agreed to give access) (Figure 2). For example, psychiatric notes are shown in 17 of 21 regions.9 Functions such as prescription renewal and appointment booking are conducted on the patient portal, yet externally from the PAEHR.

Confidential: For Review Only

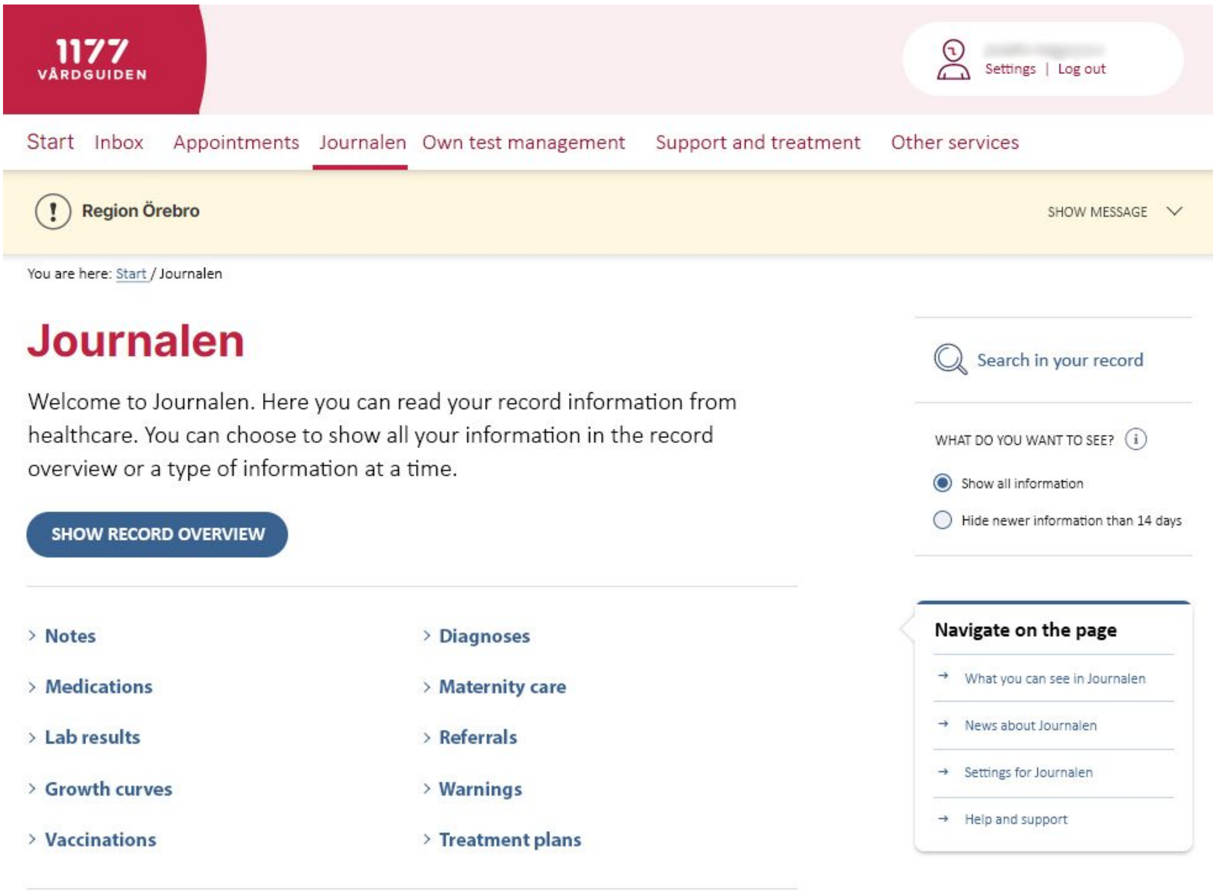

Figure 1. The patient-accessible electronic health record *Journalen* after log-in (manually translated), showing the functions and information available.[9,10] Service produced by Inera AB under the auspices of Swedish municipalities and regions.

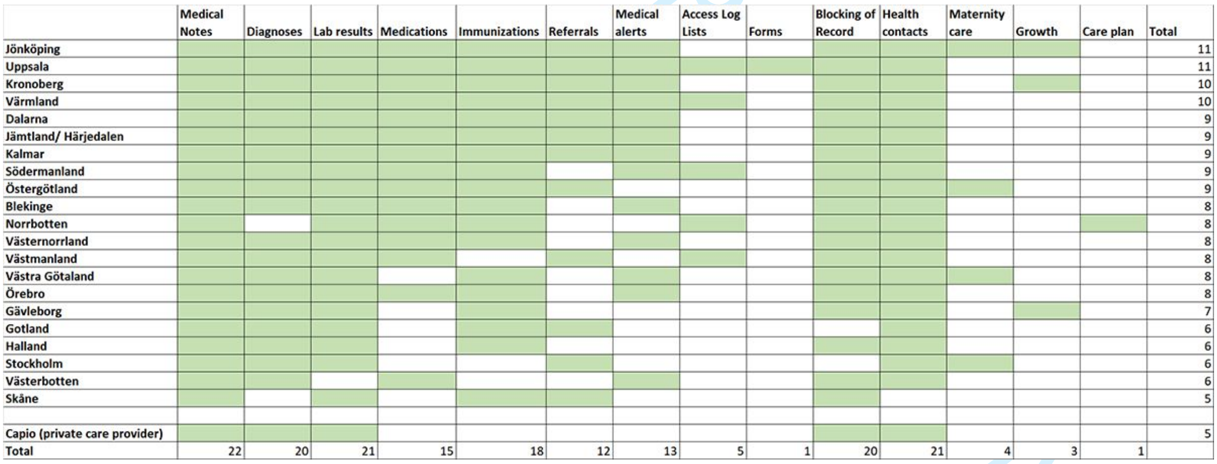

	Medical Notes	Diagnoses	Lab results	Medications	Immunizations	Referrals	Medical alerts	Access Log Lists	Forms	Blocking of Record	Health contacts	Maternity care	Growth	Care plan	Total
Jönköping															11
Uppsala															11
Kronoberg															10
Värmland															10
Dalarna															9
Jämtland/ Härjedalen															9
Kalmar															9
Södermanland															9
Östergötland															9
Blekinge															8
Norrbotten															8
Västernorrland															8
Västmanland															8
Västra Götaland															8
Örebro															8
Gävleborg															7
Gotland															6
Halland															6
Stockholm															6
Västerbotten															6
Skåne															5
Capio (private care provider)															5
Total	22	20	21	15	18	12	13	5	1	20	21	4	3	1	

Figure 2. The information shown in *Journalen* depending on region or health care provider (green squares) during the time of the survey.

Two literature reviews on pediatric patient portals found limited studies in this age group. One reviewed definition and utilization[11] while the other focused on use, views, and experiences among adolescents.[12] While expressing a lack of knowledge of how to use PAEHRs,[2,13] adolescents have stated a desire to receive such information from HCPs.[2,14] In a US study examining EHR access of adolescents' records by accounts divided into self, surrogate, and delegate, there was a significant increase of use during adolescence.[15] Furthermore, HCPs have stated the benefits of guiding adolescent patients to become more involved and activated in their care.[16,17] Research has mostly been

observational or focused on adolescents' attitudes towards patient portals, rather than experiences. In observational studies, adolescents demonstrate the use of the same portal features as adults,[18] including lab results,[15,19,20] appointment review,[15,19,20] messaging,[15,19] and medications.[20] Existing studies of adolescent users have been limited to contexts of serious illness, and these adolescents report similar benefits of reading EHRs to those of adults, such as reduced anxiety, enhanced knowledge about their illness, ability to ask informed questions and reflecting more on their health,[21] and empowerment.[5,21] A benefit unique for adolescents concerned a supported transition into adulthood.[21] To date, no studies have systematically examined how adolescents more broadly experience ORA.

Study Aim

A recent scoping review concluded that with few exceptions (Australia, Canada, New Zealand, and the United Kingdom), the entire body of literature on adolescent access to EHRs had been carried out in the United States.[11] The present research explored reasons for reading the EHRs, the perceived usefulness of information and functions in a patient portal in Sweden, and the association between frequency of use and encouragement to read the EHR.

METHODS

Study Design

Within the NORDeHEALTH research project,[22] an open survey designed to elicit the opinions and experiences of PAEHR users was conducted. The data were collected for three weeks from January to February 2022. Convenience sampling was used, and participants were recruited through the Swedish national PAEHR *Journalen*. When patients logged into *Journalen*, they received a request for voluntary survey participation together with information about the study. Thus, only active PAEHR users were invited to participate; accessing the survey without logging in was impossible. Participants provided electronic consent and there was no financial incentive. Only survey respondents between 15-19 years old were included for this paper. Due to an ethical-legal requirement of written parental consent for participants younger than 15 (The Act concerning the Ethical Review of Research Involving Humans[23]), which would complicate survey distribution, we opted to exclude those aged 13 and 14. Therefore, the first survey item after providing consent concerned age and selecting *14 or younger* triggered a redirection to survey completion. The Checklist for Reporting Results of Internet E-Surveys (CHERRIES)[24] was used to report this study.

Data Collection

An anonymous questionnaire covering 83 items was designed in English and translated into Swedish. The questionnaire included a mixture of close- and open-ended questions with various response options (yes/no, multiple choice, and free-text form). The design and selection of items were informed by prior studies.[1,18,25] Answering close-ended questions was mandatory, while free-text questions were optional. The Swedish survey was distributed over 12 pages and some items were conditionally displayed based on responses to other items. Including conditional questions, the number of items ranged between 1-10 item per page. Respondents could move back and forth between survey sections to change their answers. For this study, five items were included (see Figure 3) based on the study's aim. Three of these items used a 5-point Likert scale (ranging from disagree to agree), and two were close-ended questions. Data on use frequency was collected to enable comparison across groups. The items used in the current study are found in Figure 3, and answer options and item number can be found in Appendix A (English original and Swedish translation). The full survey and further details about the data collection procedure are found in a separate publication.[26] It should be noted that the availability of information and functions in *Journalen* varies across regions in Sweden, which may affect respondents' assessment. However, the items on perceived usefulness are formulated anticipatory (see Figure 3).

Confidential: For Review Only

1. How often do you think that you have accessed your health record during the last 12 months?
2. Did any of the following encourage or remind you to read your health record? (check all that apply)
3. Please indicate how much you disagree or agree with the following statement: I read my health record online… [reasons for reading]
4. How useful would it be to have access to the following information in the portal?
5. How useful would it be to have access to the following functions in the portal?

Figure 3. Survey items included in the study.

Confidential: For Review Only

Statistical Analysis

Only completed questionnaires were analyzed. Aside from using descriptive statistics for calculating percentages for different response options, the Fisher's exact test was used to explore differences in use frequency based on source of encouragement. Non-parametric tests were conducted due to the small sample size, and the significance level was set to 95%. The use frequency response options *10-20 times* and *More than 20 times* were merged for analysis due to a small sample. The SPSS 28 software was used for all analyses, and figures were produced using R.

Patient and public involvement

The survey was tested with the public before launch to assess its clarity and response burden, leading to minor adjustments. There was no other patient and public involvement in the design or conduct of the study.

RESULTS

Characteristics of the Participants

During the study period, 23 855 users opened the survey, and 15 867 (66.5%) started filling it in. Of these, 13 010 (82%) submitted the survey. Two (2/13 010, 0.02%) were 14 years old or younger and were excluded from the survey. Of the 13 008 respondents who completed the survey, 218 were 15-19 years old (1.68%). Of respondents aged between 15-19, 77.1% (168/218) identified as women, 17.9% (39/218) as men while 5% (11/218) selected Other. See data on survey completion in a previous publication[26] and descriptive statistics for all variables in Appendix C.

Experience with Accessing Records Online

Two out of five respondents had logged in more than 20 times (85/218, 39%) and one third had logged in 2-9 times (see Table 1).

Table 1. Participants' frequency of accessing their health records online.

**Frequency of health record access, n (%)**	**Participants (N=218)**
This is my first time	11 (5)
2 to 9 times	74 (33.9)
10-20 times	48 (22)
More than 20 times	85 (39)

Encouragement to Read the Health Records Online

One item in the survey focused on whether respondents had been encouraged or reminded by anyone or any media source to read their records online. The most common response was *nobody encouraged me* (118/218, 54.1%), see Figure 4. Of four respondents that chose *Other*, one respondent provided a free-text description, citing "myself". About one tenth had been encouraged by physicians (24/218, 11.0%) and/or nurses (26/218, 11.9%), and the combined group constituted 15.6% (34/218). Fisher's

exact tests revealed that those encouraged by physicians (*P*=.003) and nurses (*P*=.031) were more frequent users of the patient portal (detailed analysis data is available in Appendix C).

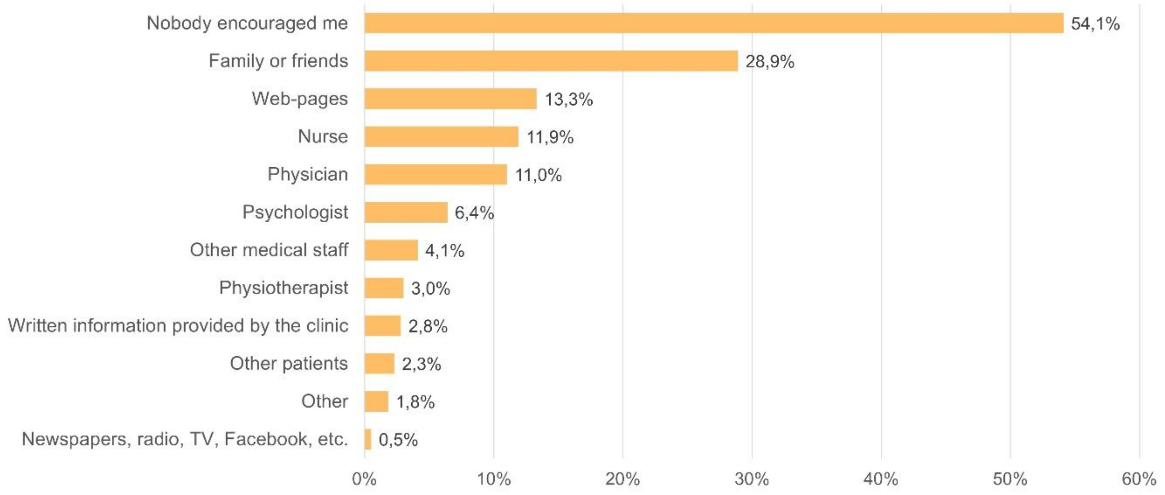

Figure 4. Encouragement to read health records online.

Reasons for Reading the Health Records Online

Four reasons were chosen by more than half of respondents: *out of general curiosity* (170/218, 78%), *to get an overview of one's medical history and/or treatment* (159/218, 72.9%), *to ensure having understood what the physician or nurse said* (149/218, 68.3%), and *to improve one's understanding for one's health issue* (138/218, 63.3%) (see Figure 5). Of 50 respondents who selected *Other*, 16 provided free-text comments. The comments included ensuring accuracy, memory reinforcement, poor notification frequency (i.e. logging in to check for new information due to lack of notifications), specification of sought information (e.g., test results, Covid-19 vaccinations), and retrieving a record copy for monetary activity compensation.

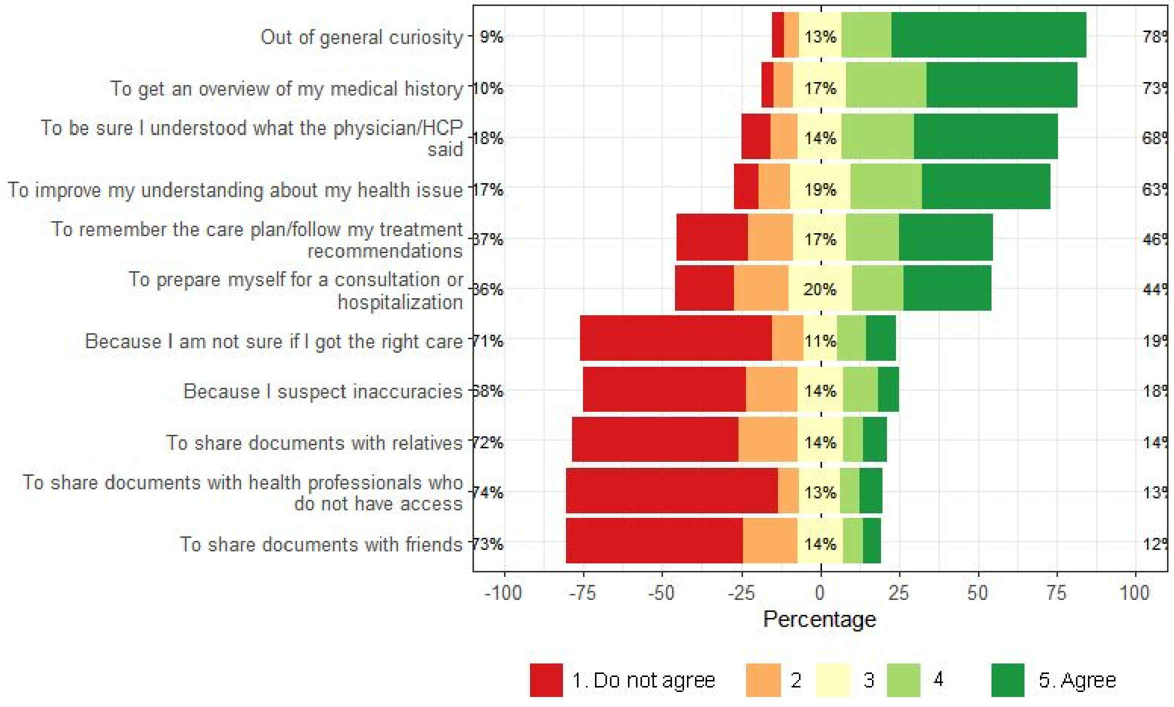

Figure 5. Adolescents' agreement with reasons for reading health records online.

Usefulness of Portal Information and Function

One survey item focused on perceived usefulness of various types of information in the records. All information types were considered useful by more than 3/4 of participants, see Figure 6.

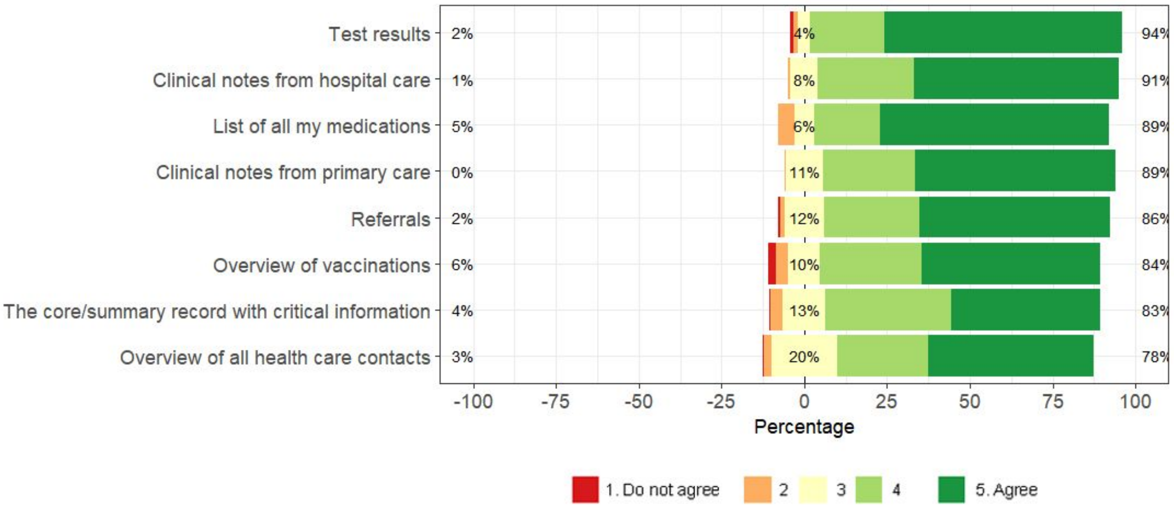

Figure 6. Adolescents' perceived usefulness of different types of patient portal information.

Another survey item focused on the perceived usefulness of various functions, most of which do not currently exist in *Journalen*. Most functions were perceived as useful by a majority of respondents, see Figure 7. Yet, four functions were considered useful by less than half of respondents: accessing information and managing services for my children and family members, contributing information about expectations for the health care visit, and blocking specific clinical notes from certain HCPs/medical staff. Of 24 participants who wrote a comment under *Other*, 7 referred to a specific function. Comments concerned the ability to view radiology images (median=4.5), diagnosis (median=3.5), dentistry records (median=5), ability to read children's records when under 18 (median=5), records from childhood (median=5), and to view appointments from gynecologic care (median=5). Other free-text answers were non-responses or unspecified. *Medical certificates and other certificates* concerned certificates from HCPs, such as COVID vaccination and for insurance and sick leave.

For Review Only

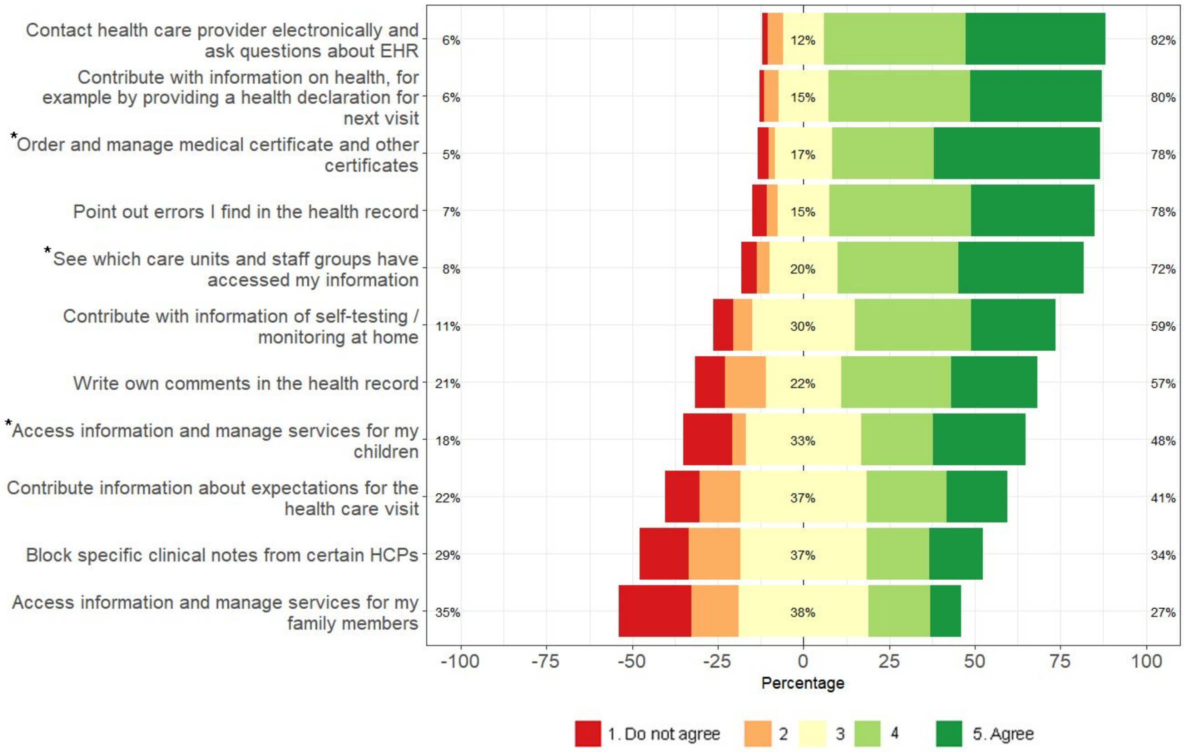

Figure 7. Adolescents' perceived usefulness of patient portal functions.

DISCUSSION

In our national survey of PAEHR experiences in Sweden, adolescents perceived all proposed clinical information as consistently useful. Ratings for portal functions varied. Common reasons for reading records were curiosity and to get an overview of medical history. Sharing clinical information was the least common reason for accessing the record. Some respondents were encouraged by HCPs to read records online, with reminders from physicians and nurses leading to more frequent use. The reasons for reading records align with previous research on adults[18] and US adolescents.[21]

While all information types were rated highly on usefulness, test results were considered the most useful. This result accords with earlier research on adolescents,[15,19,20] but also with research on parents[27,28] and general adult populations.[18] Unsurprisingly, adolescents rated the ability to manage family members or children low. The function perceived as the third most useful was the ability to order and manage various certificates. This was likely associated with the timing of survey distribution, as the ongoing COVID-19 pandemic required Swedish citizens to present COVID-19 certificates for traveling and attending events. The boost of PAEHR use as a result of the COVID-19 pandemic has been mentioned previously.[29] Related to this, it is somewhat unexpected that immunizations were rated among the least useful types of information. However, Swedish regions differed in managing COVID-19 vaccination through the national e-service or other apps, and not all regions displayed information on vaccinations. Another finding was that adolescents do not read their PAEHRs to share information. This finding is consistent with a previous survey study based in Canada, where adolescents without access to their records anticipated not being likely to share information from the records with others.[30]

Only physician and nurse encouragement (compared to encouragement from psychologists and physical therapists) was linked to higher use frequency, indicating their perceived importance and the influence of social factors in eHealth adoption.[31] Possibly, information provided by physicians and nurses required the patient's independent care management, such as treatment steps or medication instructions. Encouragement from healthcare providers and family/friends has increased PAEHR use in adults,[32] suggesting that this may be an important factor influencing adoption and use also in adolescents. Lack of HCP encouragement aligns with research on adults in the United States[33] and Sweden[34], and may stem from concerns about medical literacy, increased workload, uncertainty about

For Review Only

visible information, and shared access with parents.[2,5,35–37] Not encouraging adolescents to read their records may limit PAEHR usage and miss an opportunity for early patient involvement.[16] Increased PAEHR usage among adolescents can motivate policy-making stakeholders and EHR vendors to improve patient portals for this population.[5]

This study has limitations. Response biases in self-report surveys are unknown. Survey availability on the national patient portal may have influenced respondents' decisions based on their competence and skills in using the service, potentially causing positive bias as most adolescent respondents were frequent users. The majority of participants were women, which is consistent with adult and previous adolescent PAEHR users but represents a strong gender imbalance. Only 1.7% of the total respondents were aged 15-19, possibly due to younger individuals being less affected by health conditions and less engaged in personal health management.

The present results provide a broader understanding of adolescents reading their EHRs, however further work is required to establish the viability of the findings in other settings. Several questions remain, relating to for example eHealth literacy and views on confidentiality and security. It should be noted that the current study included only a subset of items from a larger survey, and there is an intention to publish additional findings on the adolescent population. Regarding our research methodology, we offer a few suggestions for future work. First, frequency of use was defined by how many times (first time, 2-5 times etc.) the users accessed their records in the past 12 months. Another approach is to ask how often users access their records (daily, weekly, monthly etc.), which allows for an understanding of the visit regularity. Second, further research may benefit from providing a clearer distinction between a specific PAEHR service and patients' ORA more broadly. Lastly, including a larger and more evenly gender-distributed sample and specific patient groups with chronic diseases could furnish greater insights into adolescent users' experiences.

Data availability statement
Data are available from the corresponding author on reasonable request.

Ethics statements
Patient consent for publication
Electronic consent obtained from survey respondents.

Ethics approval
Ethical approval for this study was obtained from the Regional Ethical Review Board in Uppsala, Sweden (EPN 2021/05229). All respondents received study information and provided informed consent.

Abbreviations
EHR: electronic health record
HCP: healthcare professional
ORA: online record access
PAEHR: patient-accessible electronic health record

FOOTNOTES

Contributors: JH wrote the manuscript, created the tables, and designed the figures. MH, CB, IS, JM, ÅC, and HR contributed to the study design. All authors read the manuscript, provided feedback, and approved the final draft for submission.

Funding: This work was supported by NordForsk through the funding to Nordic eHealth for Patients: Benchmarking and Developing for the Future, NORDeHEALTH, (Project #100477), the Swedish Research Council for Health, Working Life and Welfare (FORTE) through the funding to Beyond Implementation of eHealth (Project #2020-01229).

Confidential: For Review Only

Competing interests: None.

Patient and public involvement: Patients and/or the public were involved in survey testing but otherwise not involved in the design, or conduct, or reporting, or dissemination plans of this research.

Provenance and peer review: Not commissioned; externally peer reviewed.

Confidential: For Review Only

REFERENCES

1. Walker J, Leveille S, Bell S, et al. OpenNotes After 7 Years: Patient Experiences With Ongoing Access to Their Clinicians' Outpatient Visit Notes. J Med Internet Res. 2019;21(5):e13876. doi:10.2196/13876
2. Beaton L, Williams I, Sanci L. Exploring adolescent and clinician perspectives on Australia. Aust J Prim Health. Published online March 15, 2021. doi:10.1071/PY20169
3. Bayer R, Santelli J, Klitzman R. New Challenges for Electronic Health Records: Confidentiality and Access to Sensitive Health Information About Parents and Adolescents. JAMA. 2015;313(1):29. doi:10.1001/jama.2014.15391
4. Ford CA. Foregone Health Care Among Adolescents. JAMA. 1999;282(23):2227. doi:10.1001/jama.282.23.2227
5. Carlson JL, Goldstein R, Buhr T, Buhr N. Teenager, parent, and clinician perspectives on the electronic health record. Pediatrics. 2020;145(3). doi:10.1542/peds.2019-0193
6. Goldstein RL, Anoshiravani A, Svetaz MV, Carlson JL. Providers' Perspectives on Adolescent Confidentiality and the Electronic Health Record: A State of Transition. J Adolesc Health. 2020;66:296-300. doi:10.1016/j.jadohealth.2019.09.020
7. Stablein T, Loud KJ, DiCapua C, Anthony DL. The Catch to Confidentiality: The Use of Electronic Health Records in Adolescent Health Care. J Adolesc Health. 2018;62(5):577-582. doi:10.1016/j.jadohealth.2017.11.296
8. Statistik för Ineras tjänster [Statistics for Inera's services]. Inera AB. Accessed August 25, 2023. https://www.inera.se/tjanster/statistik-for-ineras-tjanster/
9. Bärkås A, Scandurra I, Rexhepi H, Blease C, Cajander Å, Hägglund M. Patients' Access to Their Psychiatric Notes: Current Policies and Practices in Sweden. Int J Environ Res Public Health. 2021;18(17):9140. doi:10.3390/ijerph18179140
10. Journalen. Inera. Published December 2022. Accessed December 8, 2022. https://journalen.1177.se/
11. Bush RA, Connelly CD, Fuller M, Pérez A. Implementation of the Integrated Electronic Patient Portal in the Pediatric Population: A Systematic Review. Telemed J E-Health Off J Am Telemed Assoc. 2016;22(2):144-152. doi:10.1089/tmj.2015.0033
12. Hagström J, Blease C, Haage B, Scandurra I, Hansson S, Hägglund M. Use, Views of and Experiences of Web-Based Access to Electronic Health Records for Children, Adolescents, and Parents: Scoping Review. J Med Internet Res. Published online 2022. doi:10.2196/40328
13. Miklin DJ, Vangara SS, Delamater AM, Goodman KW. Understanding of and Barriers to Electronic Health Record Patient Portal Access in a Culturally Diverse Pediatric Population. JMIR Med Inform. 2019;7(2):e11570. doi:10.2196/11570
14. Bergman DA, Brown NL, Wilson S. Teen Use of a Patient Portal: A Qualitative Study of Parent and Teen Attitudes. Perspect Health Inf Manag. 2008;5:13.
15. Steitz BD, Cronin RM, Davis SE, Yan E, Jackson GP. Long-term patterns of patient portal use for pediatric patients at an academic medical center. Appl Clin Inform. 2017;8(3):779-793. doi:10.4338/ACI-2017-01-RA-0005
16. Bourgeois FC, DesRoches CM, Bell SK. Ethical Challenges Raised by OpenNotes for Pediatric and Adolescent Patients. Pediatrics. 2018;141(6):e20172745. doi:10.1542/peds.2017-2745

17. Lam BD, Bourgeois F, DesRoches CM, Dong Z, Bell SK. Attitudes, experiences, and safety behaviours of adolescents and young adults who read visit notes: Opportunities to engage patients early in their care. Future Heal J. 2021;8(3):e585-e592. doi:10.7861/fhj.2021-0118
18. Moll J, Rexhepi H, Cajander Å, et al. Patients' Experiences of Accessing Their Electronic Health Records: National Patient Survey in Sweden. J Med Internet Res. 2018;20(11):e9492. doi:10.2196/jmir.9492
19. Calman N, Pfister HR, Lesnewski R, Hauser D, Shroff N. Electronic Access to Adolescents' Health Records: Legal, Policy, and Practice Implications. Vol 22.; 2014:11-14. https://www.aafp.org/fpm/2015/0300/p11.html
20. Thompson LA, Martinko T, Budd P, Mercado R, Schentrup AM. Meaningful use of a confidential adolescent patient portal. J Adolesc Health. 2016;58(2):134-140. doi:10.1016/j.jadohealth.2015.10.015
21. Hong MK, Wilcox L, Feustel C, Wasileski-Masker K, Olson TA, Simoneaux SF. Adolescent and Caregiver use of a Tethered Personal Health Record System. AMIA Annu Symp Proc AMIA Symp. 2016;2016:628-637.
22. Hägglund M. Nordic countries lead new initiative on patient access to EHRs. TheBMJopinion. Published May 18, 2021. Accessed January 16, 2022. https://blogs.bmj.com/bmj/2021/05/18/maria-hagglund-nordic-countries-lead-new-initiative-on-patient-access-to-ehrs/
23. Riksdagsförvaltningen. 2003:460.; 2003. Accessed August 3, 2022. https://www.riksdagen.se/sv/dokument-lagar/dokument/svensk-forfattningssamling/lag-2003460-om-etikprovning-av-forskning-som_sfs-2003-460
24. Eysenbach G. Improving the Quality of Web Surveys: The Checklist for Reporting Results of Internet E-Surveys (CHERRIES). J Med Internet Res. 2004;6(3):e34. doi:10.2196/jmir.6.3.e34
25. Zanaboni P, Kummervold PE, Sørensen T, Johansen MA. Patient use and experience with online access to electronic health records in Norway: Results from an online survey. J Med Internet Res. 2020;22(2). doi:10.2196/16144
26. Hägglund M, Kharko A, Hagström J, et al. The NORDeHEALTH 2022 Patient Survey: A cross-sectional survey of national patient portal users in Norway, Sweden, Finland, and Estonia. Published online 2023. Accessed June 9, 2023. https://preprints.jmir.org/preprint/47573
27. Ahlers-Schmidt CR, Nguyen M. Parent Intention to Use a Patient Portal as Related to Their Children Following a Facilitated Demonstration. Telemed E-Health. 2013;19(12):979-981. doi:10.1089/tmj.2013.0041
28. Thompson LA, Black EW, Saliba H, Schentrup AM. Parents' knowledge of and opinions about healthcare laws and technology in primary care. Inform Prim Care. 2012;20(1):69-74. doi:10.14236/jhi.v20i1.49
29. Blease C, Salmi L, Hägglund M, Wachenheim D, DesRoches C. COVID-19 and Open Notes: A New Method to Enhance Patient Safety and Trust. JMIR Ment Health. 2021;8(6):e29314. doi:10.2196/29314
30. Frazer C, Ratchford F, Roch J. Enabling Adolescent Electronic Access to Personal Health Information. Stud Health Technol Inform. 2017;234:115-119.
31. Venkatesh V, Morris MG, Davis GB, Davis FD. User Acceptance of Information Technology: Toward a Unified View. MIS Q. 2003;27(3):425-478. doi:10.2307/30036540
32. Mukhopadhyay S, Basak R, Carpenter D, Reithel BJ. Patient use of online medical records: an application of technology acceptance framework. Inf Comput Secur. 2019;28(1):97-115. doi:10.1108/ICS-07-2019-0076
33. DesRoches CM, Leveille S, Bell SK, et al. The Views and Experiences of Clinicians Sharing Medical Record Notes With Patients. JAMA Netw Open. 2020;3(3):e201753. doi:10.1001/jamanetworkopen.2020.1753
34. Rexhepi H, Moll J, Huvila I. Online electronic healthcare records: Comparing the views of cancer patients and others. Health Informatics J. 2020;26(4):2915-2929. doi:10.1177/1460458220944727

Confidential: For Review Only

35. Hagström J, Blease C, Haage B, Scandurra I, Hansson S, Hägglund M. Views, Use, and Experiences of Web-Based Access to Pediatric Electronic Health Records for Children, Adolescents, and Parents: Scoping Review. J Med Internet Res. 2022;24(11):e40328. doi:10.2196/40328
36. Grünloh C, Myreteg G, Cajander Å, Rexhepi H. "Why Do They Need to Check Me?" Patient Participation Through eHealth and the Doctor-Patient Relationship: Qualitative Study. J Med Internet Res. 2018;20(1):e8444. doi:10.2196/JMIR.8444
37. Petersson J, Backman C. Patient-accessible online health records: Reconfigurations of clinical rhythms and doctors' front- and backstage spaces. Soc Sci Med. 2022;292:114635. doi:10.1016/j.socscimed.2021.114635

Confidential: For Review Only

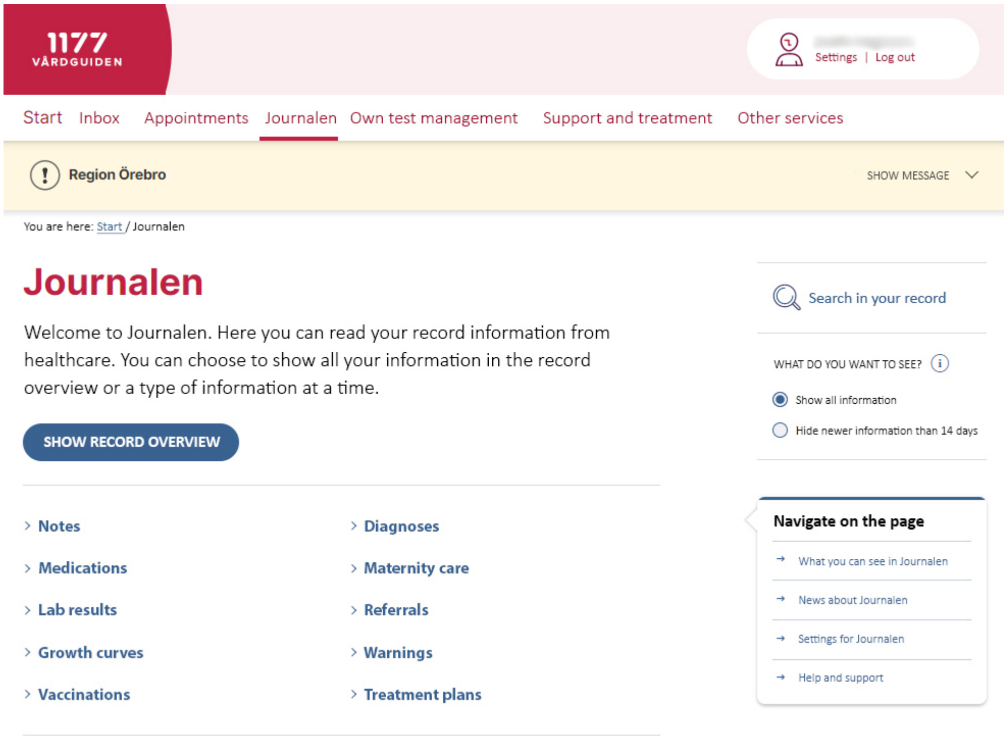

The patient-accessible electronic health record Journalen after log-in (manually translated), showing the functions and information available.[10] Service produced by Inera AB under the auspices of Swedish county councils and regions.

346x258mm (72 x 72 DPI)

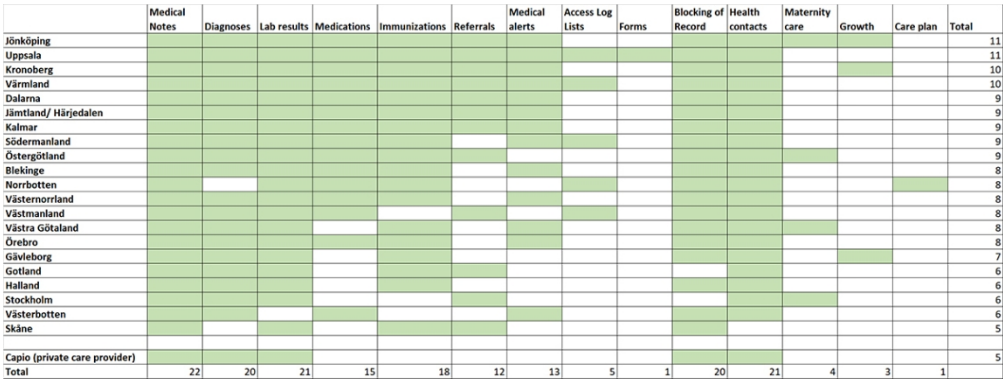

	Medical Notes	Diagnoses	Lab results	Medications	Immunizations	Referrals	Medical alerts	Access Log Lists	Forms	Blocking of Record	Health contacts	Maternity care	Growth	Care plan	Total
Jönköping															11
Uppsala															11
Kronoberg															10
Värmland															10
Dalarna															9
Jämtland/ Härjedalen															9
Kalmar															9
Södermanland															9
Östergötland															9
Blekinge															8
Norrbotten															8
Västernorrland															8
Västmanland															8
Västra Götaland															8
Örebro															8
Gävleborg															7
Gotland															6
Halland															6
Stockholm															6
Västerbotten															6
Skåne															5
Capio (private care provider)															5
Total	22	20	21	15	18	12	13	5	1	20	21	4	3	1	

The information shown in Journalen depending on region or health care provider (green squares) during the time of the survey.

423x159mm (72 x 72 DPI)

1. How often do you think that you have accessed your health record during the last 12 months?
2. Did any of the following encourage or remind you to read your health record? (check all that apply)
3. Please indicate how much you disagree or agree with the following statement: I read my health record online… [reasons for reading]
4. How useful would it be to have access to the following information in the portal?
5. How useful would it be to have access to the following functions in the portal?

Survey questions included in the study.

338x110mm (300 x 300 DPI)

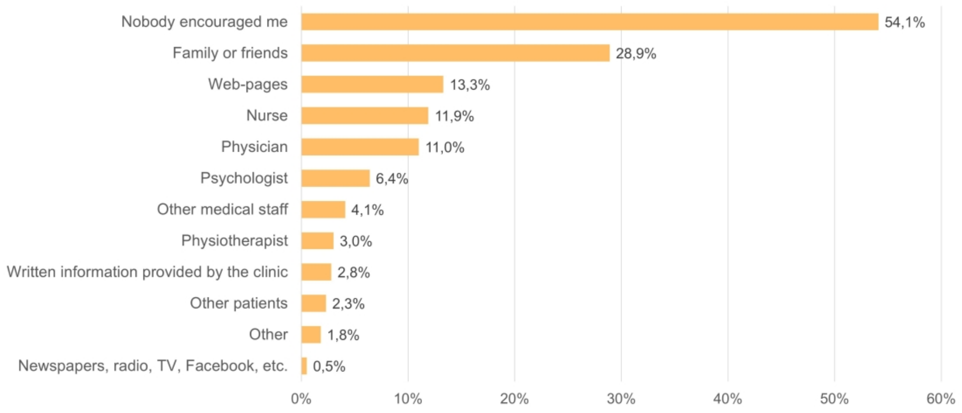

Encouragement to read health records online.

338x146mm (300 x 300 DPI)

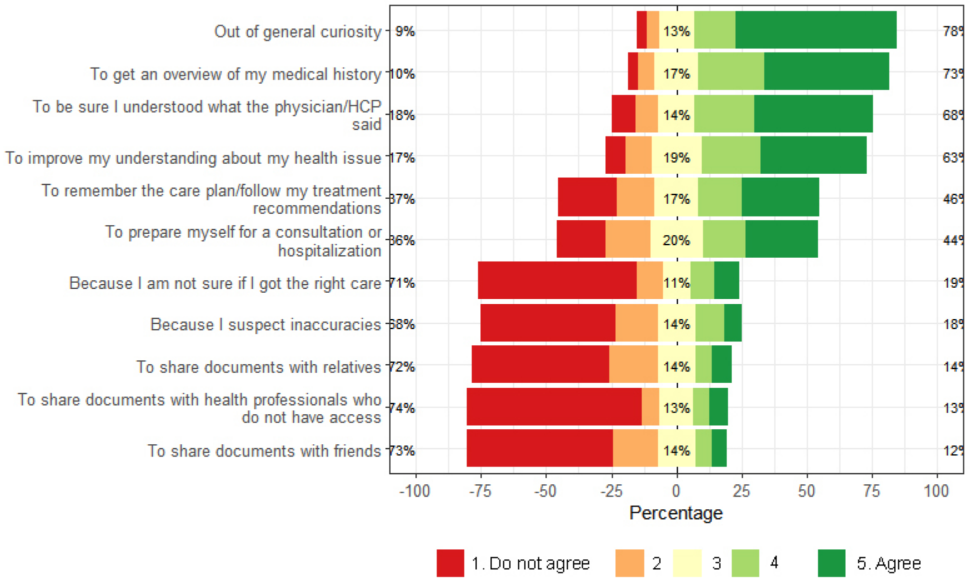

Adolescents' agreement with reasons for reading health records online.

282x169mm (72 x 72 DPI)

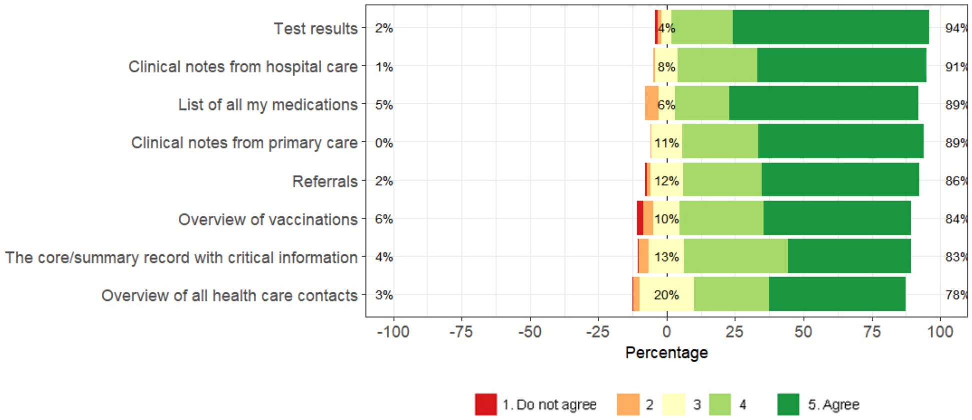

Adolescents’ perceived usefulness of different types of patient portal information.

389x168mm (72 x 72 DPI)

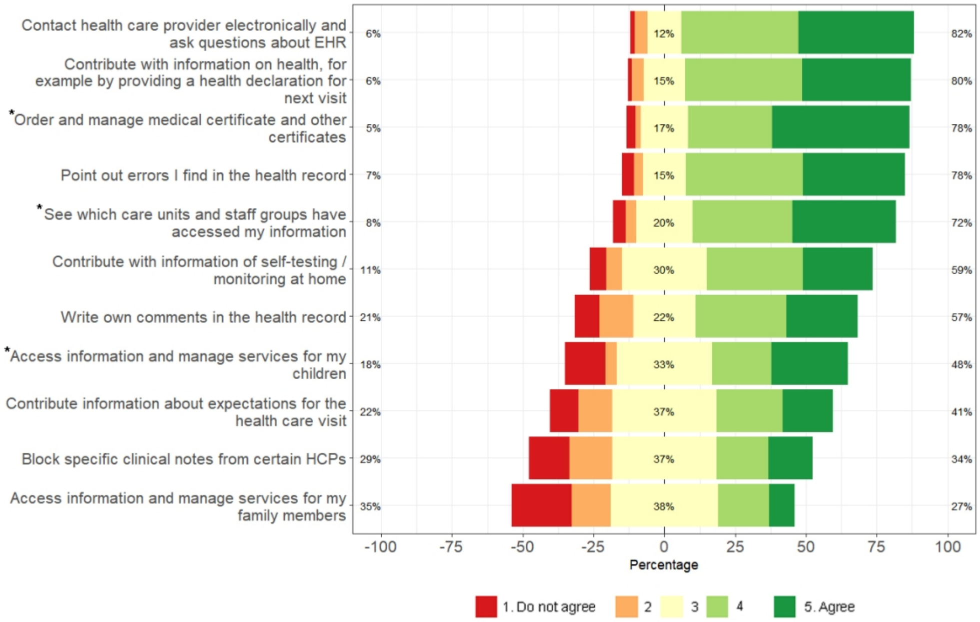

Adolescents' perceived usefulness of patient portal functions. Legend: *Functions that exist in all or some regions in Sweden. Only adult users over 18 years old have access to their children's records.

470x296mm (72 x 72 DPI)

Appendix A

Table 1. Original survey created in English

Number in survey	Question	Response alternatives
6	**How often do you think that you have accessed your health record during the last 12 months?**	1 - This is my first time<br>2 - 2 to 9 times<br>3 - 10 to 20 times<br>4 - More than 20 times
7	**Did any of the following encourage or remind you to read your health record?**	HCPs: Physician<br>HCPs: Nurse<br>HCPs: Psychologist<br>HCPs: Physiotherapist<br>HCPs: Other medical staff<br>Written information provided by the hospital/healthcare facility /clinic<br>Family or friends<br>Web-pages, such as the National health portal web-page, etc.<br>Newspapers, radio, TV, Facebook, etc.<br>Other patients<br>Nobody encouraged me<br>Other
8	**I read my health record online…**	1 - Disagree,<br>2, 3, 4,<br>5 - Agree
		out of general curiosity
		to improve my understanding about my health issue
		to prepare myself for a consultation or hospitalization
		to get an overview of my medical history and/or treatment
		to be sure I understood what the physician/healthcare professional said
		to remember the care plan/follow my treatment recommendations
		because I suspect inaccuracies
		to share documents with relatives
		to share documents with friends
		to share documents with health professionals who do not have access
		because I am not sure if I got the right care
		Other, please specify

38	**How useful would it be to have access to the following information in the portal?**		1 - Yes, 2 - No, 3 - Don't know
		The core/summary record with critical information	
		Referrals (content and how it is handled in care)	
		List of all pharmaceuticals/my medications	
		Overview of vaccinations	
		Results of tests	
		Clinical notes from primary care	
		Clinical notes from hospital care	
		Overview of all health care contacts	
39	**How useful would it be to have access to the following functions in the portal?**		1 - Not useful at all<br>2 - Not very useful<br>3 - Neither useful nor useless<br>4 - Useful<br>5 - Very useful
		Ability to access information and manage services for my children	
		Ability to access information and manage services for my family members	
		See which care units and staff groups have accessed my information (see log data)	
		Ability to block certain medical records from access by other medical staff	
		Ability to contact health care provider electronically and ask questions about medical record	
		Ability to point out errors I find in the health record	
		Ability to write own comments to text in the health record	
		Contribute with information on health, for example by providing a health declaration for next visit	
		Contribute with information of self-testing/monitoring at home	
		Contribute information about expectations for the health care visit	
		Ability to order and manage medical certificate and other certificates	
		Other	

Table B2. Swedish translation of survey

Number in survey	Question	Response alternatives
6	**Hur ofta har du läst i din journal under de senaste 12 månaderna?**	1 - This is my first time<br>2 - 2 to 9 times<br>3 - 10 to 20 timesa<br>4 - More than 20 times
7	**Har du blivit uppmuntrad eller påmind att läsa din journal av någon/något av följande:**	Vårdpersonal: Läkare<br>Vårdpersonal: Sjuksköterska<br>Vårdpersonal: Psykolog<br>Vårdpersonal: Fysioterapeut/sjukgymnast<br>Vårdpersonal: Annan vårdpersonal<br>Skriftlig information från sjukhuset/vårdinrättning/klinik<br>Familj eller vänner<br>Webbsidor, såsom 1177.se Vårdguiden, etc.<br>Tidningar, radio, TV, Facebook, etc.<br>Andra patienter<br>Ingen har uppmuntrat eller påmint mig<br>Annat
8	**Markera i vilken utsträckning du instämmer med följande påståenden:**	1 - Instämmer inte, 2, 3, 4,<br>5 - Instämmer
	Av nyfikenhet	
	För att förbättra min förståelse för mitt hälsotillstånd	
	För att förbereda mig själv för ett vårdbesök eller sjukhusvistelse	
	För att få en överblick av min hälsohistoria och/eller bakgrund	
	För att försäkra mig om att jag förstått vad läkaren/vårdpersonalen sagt	
	För att komma ihåg vårdplanen/följa mina behandlingsrekommendationer	
	För att jag misstänker felaktigheter	
	För att dela med mig av information till släkt	
	För att dela med mig av information till vänner	
	För att dela med mig av information till vårdpersonal som inte har tillgång till min journal	
	För att jag är osäker på om jag fått rätt vård	
	Annat, specificera gärna	

38	**Hur användbart skulle det vara för dig att ha tillgång till följande information i Journalen?**		1 - Inte alls användbart<br>2 - Inte särskilt användbart<br>3 - Varken eller<br>4 - Användbart<br>5 - Väldigt användbart
		En sammanfattning av journalen med viktig information	
		Remisser (innehåll och status)	
		Lista över mina läkemedel/mediciner	
		Vaccinationer	
		Provsvar	
		Journalanteckningar från primärvården	
		Journalanteckningar från specialistvården	
		Överblick av samtliga kontakter med vården	
39	**Hur användbart skulle det vara för dig att ha tillgång till följande funktioner i Journalen?**		1 - Inte alls användbart<br>2 - Inte särskilt användbart<br>3 - Varken eller<br>4 - Användbart<br>5 - Väldigt användbart
		Möjlighet att ta del av information och hantera tjänster för mina barn	
		Möjlighet att ta del av information och hantera tjänster för andra familjemedlemmar	
		Möjlighet att kunna blockera specifika journalanteckningar för viss vårdpersonal	
		Se vilka vårdinrättningar och personal som har läst i min journal (loggar)	
		Möjlighet att kunna kontakta vårdpersonal online för att ställa frågor om min journal	
		Möjlighet att påpeka felaktigheter i journalen	
		Möjlighet att skriva egna kommentarer i journalen	
		Möjlighet att bidra med information om min hälsa, t ex genom att fylla i en hälsodeklaration, inför ett besök	
		Möjlighet att bidra med information från självtest/monitorering hemma	
		Möjlighet att bidra med information om mina förväntningar på vårdbesöket	
		Möjlighet att beställa och hantera sjukskrivning, medicinsk intyg och andra dokument (vaccinationsintyg t ex)	
		Annat	

Appendix B

Detailed results from the survey questions chosen for this study.

Encouragement to Read.

**Did any of the following encourage or remind you to read your health record? n (%)**	**Yes**	**No**
HCPs: Physician	24 (11.0)	194 (89.0)
HCPs: Nurse	26 (11.9)	192 (88.1)
HCPs: Psychologist	14 (6.4)	204 (93.6)
HCPs: Physiotherapist	6 (2.8)	212 (97.2)
HCPs: Other medical staff	9 (4.1)	209 (95.9)
Written information provided by the hospital/healthcare facility/clinic	12 (5.5)	206 (94.5)
Family or friends	63 (28.9)	155 (71.1)
Web-pages, such as the National health portal web-page, etc.	29 (13.3)	189 (86.7)
Newspapers, radio, TV, Facebook, etc.	1 (0.5)	217 (99.5)
Other patients	5 (2.3)	213 (97.7)
Nobody encouraged me	118 (54.1)	100 (45.9)
Other	4 (1.8)	214 (98.2)

Reasons for reading health records online.

**I read my health record online…, n** (%)	**1. Disagree**	**2**	**3**	**4**	**5. Agree**
… out of general curiosity	9 (4.1)	10 (4.6)	29 (13.3)	35 (16.1)	135 (61.9)
… to improve my understanding about my health issue	16 (7.3)	22 (10.1)	42 (19.3)	49 (22.5)	89 (40.8)
… to prepare myself for a consultation or hospitalization	41 (18.8)	37 (17.0)	44 (20.2)	36 (16.5)	60 (27.5)
… to get an overview of my medical history and/or treatment	8 (3.7)	14 (6.4)	37 (17.0)	55 (25.2)	104 (47.7)
… to be sure I understood what the physician/healthcare professional said	20 (9.2)	19 (8.7)	30 (13.8)	50 (22.9)	99 (45.4)
… to remember the care plan/follow my treatment recommendations	49 (22.5)	32 (14.7)	36 (16.5)	37 (17.0)	64 (29.4)
… because I suspect inaccuracies	113 (51.8)	35 (16.1)	31 (14.2)	25 (11.5)	14 (6.4)
… to share documents with relatives	115 (52.8)	41 (18.8)	31 (14.2)	14 (6.4)	17 (7.8)
… to share documents with friends	122 (56.0)	38 (17.4)	31 (14.2)	14 (6.4)	13 (6.0)
… to share documents with healthcare professionals who do not have access	146 (67.0)	15 (6.9)	28 (12.8)	13 (6.0)	16 (7.3)
… because I am not sure if I got the right care	132 (60.6)	22 (10.1)	23 (10.6)	20 (9.2)	21 (9.6)
Other	168 (77.1)	20 (9.2)	5 (2.3)	3 (1.4)	22 (10.1)

Perceived usefulness of various types of information.

**Information**	Not useful at all	Not very useful	Neither useful nor useless	Useful	Very useful
The core/summary record with critical information	1 (0.5)	8 (3.7)	28 (12.8)	83 (38.1)	98 (45.0)
Referrals (content and how it is handled in care)	1 (0.5)	3 (1.4)	26 (11.9)	63 (28.9)	125 (11.9)
List of all pharmaceuticals / my medications	0 (0)	11 (5.0)	13 (6.0)	43 (19.7)	151 (69.3)
Overview of vaccinations	5 (2.3)	8 (3.7)	21 (9.6)	67 (30.7)	117 (53.7)
Results of tests (not available in all regions)	2 (0.9)	3 (1.4)	8 (3.7)	49 (22.5)	156 (71.6)
Clinical notes from primary care	0 (0)	1 (0.5)	24 (11.0)	61 (28.0)	132 (60.6)
Clinical notes from hospital care	0 (0)	2 (0.9)	18 (8.3)	63 (28.9)	135 (61.9)
Overview of all health care contacts	1 (0.5)	5 (2.3)	43 (19.7)	60 (27.5)	109 (50)

Perceived usefulness of various functions.

**Function**	Not useful at all	Not very useful	Neither useful nor useless	Useful	Very useful
Ability to access information and manage services for my children (not available in all regions).	31 (14.2)	9 (4.1)	73 (33.5)	46 (21.1)	59 (27.1)
Ability to access information and manage services for my family members.	46 (21.1)	30 (13.8)	83 (38.1)	39 (17.9)	20 (9.2)
Ability to block specific clinical notes from certain healthcare professionals/medical staff.	31 (14.2)	33 (15.1)	80 (36.7)	40 (18.3)	34 (15.6)
See which care units and staff groups have accessed my information (see log data).	10 (4.6)	8 (3.7)	43 (19.7)	77 (35.3)	80 (36.7)
Ability to contact health care provider electronically and ask questions about medical record.	3 (1.4)	10 (4.6)	26 (11.9)	90 (41.3)	89 (40.8)
Ability to point out errors I find in the health record.	9 (4.1)	7 (3.2)	33 (15.1)	90 (41.3)	79 (36.2)
Ability to write own comments to text in the health record.	19 (8.7)	26 (11.9)	48 (22.0)	70 (32.1)	55 (25.2)
Contribute with information on health, for example by providing a health declaration for next visit.	3 (1.4)	9 (4.1)	32 (14.7)	90 (41.3)	84 (38.5)
Contribute with information of self-testing/monitoring at home.	13 (6.0)	12 (5.5)	65 (29.8)	74 (33.9)	54 (24.8)
Contribute information about expectations for the health care visit.	22 (10.1)	26 (11.9)	80 (36.7)	51 (23.4)	39 (17.9)
Ability to order and manage medical certificate and other certificates.	7 (3.2)	4 (1.8)	36 (16.5)	65 (29.8)	106 (48.6)

Appendix C

Relationship between encouragement to read records online and use frequency.

**Encouragement to read records**	**n (%)**	**p**
HCPs: Physician	24 (11.0)	.003*
HCPs: Nurse	26 (11.9)	.031*
HCPs: Psychologist	14 (6.4)	.479
HCPs: Physiotherapist	6 (2.8)	1.000
HCPs: Other medical staff	9 (4.1)	.195
Written information provided by the hospital / healthcare facility/clinic	12 (5.5)	.759
Family or friends	63 (28.9)	.399
Web-pages, such as the National health portal web-page, etc.	29 (13.3)	.578
Newspapers, radio, TV, Facebook, etc.	1 (0.5)	1.000
Other patients	5 (2.3)	.736
Nobody encouraged me	118 (54.1)	.141

BMJ Paediatrics Open

Views and experiences of adolescents reading their health records online: A national survey

Journal:	*BMJ Paediatrics Open*
Manuscript ID	bmjpo-2023-002258.R1
Article Type:	Original research
Date Submitted by the Author:	23-Nov-2023
Complete List of Authors:	Hagström, Josefin; Uppsala Universitet, Department of Women's and Children's Health<br>Blease, Charlotte; Uppsala Universitet, Department of Women's and Children's Health; Harvard Medical School, Dept of Psychiatry, Beth Israel Deaconess Medical Center<br>Scandurra, Isabella; Örebro University, Informatics, School of Business<br>Moll, Jonas; Örebro University, Informatics, School of Business<br>Cajander, Åsa; Uppsala Universitet, Department of Information Technology<br>Rexhepi, Hanife; University of Skövde, School of Informatics<br>Hägglund, Maria; Uppsala Universitet, Department of Women's and Children's Health; Uppsala University Hospital
Keywords:	Adolescent Health, Health services research, Data Collection

SCHOLARONE™
Manuscripts

I, the Submitting Author has the right to grant and does grant on behalf of all authors of the Work (as defined in the below author licence), an exclusive licence and/or a non-exclusive licence for contributions from authors who are: i) UK Crown employees; ii) where BMJ has agreed a CC-BY licence shall apply, and/or iii) in accordance with the terms applicable for US Federal Government officers or employees acting as part of their official duties; on a worldwide, perpetual, irrevocable, royalty-free basis to BMJ Publishing Group Ltd ("BMJ") its licensees and where the relevant Journal is co-owned by BMJ to the co-owners of the Journal, to publish the Work in this journal and any other BMJ products and to exploit all rights, as set out in our licence.

The Submitting Author accepts and understands that any supply made under these terms is made by BMJ to the Submitting Author unless you are acting as an employee on behalf of your employer or a postgraduate student of an affiliated institution which is paying any applicable article publishing charge ("APC") for Open Access articles. Where the Submitting Author wishes to make the Work available on an Open Access basis (and intends to pay the relevant APC), the terms of reuse of such Open Access shall be governed by a Creative Commons licence – details of these licences and which Creative Commons licence will apply to this Work are set out in our licence referred to above.

Other than as permitted in any relevant BMJ Author's Self Archiving Policies, I confirm this Work has not been accepted for publication elsewhere, is not being considered for publication elsewhere and does not duplicate material already published. I confirm all authors consent to publication of this Work and authorise the granting of this licence.

Confidential: For Review Only

TITLE PAGE

Article title: Views and experiences of adolescents reading their health records online: A national survey

Author information:
Corresponding author:
Josefin Hagström, MSc (ORCID: 0000-0003-2835-0259)
Participatory eHealth and Health Data Research Group, Department of Women's and Children's Health, Uppsala University, Uppsala, Sweden

josefin.hagstrom@kbh.uu.se
+46184715080

Co-authors:
Charlotte Blease PhD[1,2] (ORCID: 0000-0002-0205-1165)
Isabella Scandurra PhD[3] (ORCID: 0000-0002-2597-1079)
Jonas Moll PhD[3] (ORCID: 0000-0002-4772-4730)
Åsa Cajander PhD[4] (ORCID: 0000-0001-7472-2215)
Hanife Rexhepi PhD[5] (ORCID: 0000-0001-8957-9853)
Maria Hägglund PhD[1, 6] (ORCID: 0000-0002-6839-3651)

[1] Participatory eHealth and Health Data Research Group, Department of Women's and Children's Health, Uppsala University, Uppsala, Sweden
[2] Digital Psychiatry, Dept of Psychiatry, Beth Israel Deaconess Medical Center, Harvard Medical School, Boston, MA, USA
[3] Centre for Empirical Research on Information Systems (CERIS), Informatics, School of Business, Örebro University, Örebro, Sweden
[4] Department of Information Technology, Uppsala University, Uppsala, Sweden
[5] School of Informatics, University of Skövde, Sweden
[6] Uppsala University Hospital

Disclaimers: The views expressed in the submitted article are his or her own and not an official position of the institution or funder.

Source(s) of support: This work was supported by NordForsk through the funding to Nordic eHealth for Patients: Benchmarking and Developing for the Future, NORDeHEALTH, (Project #100477), the Swedish Research Council for Health, Working Life and Welfare (FORTE) through the funding to Beyond Implementation of eHealth (Project #2020-01229).

Word count: 2499

Number of figures: 7

Number of tables: 2

Key words: adolescents; adolescent health; Patient Accessible Electronic Health Record (PAEHR); Electronic Health Record (EHR); patient portal; national survey; ehealth

References: 38

Disclosure of relationships and activities: There are no conflicts of interest to declare.

Confidential: For Review Only

ABSTRACT

Background: Having online access to electronic health records (EHRs) may help patients become engaged in their care at an early age. However, little is known about adolescents using patient portals. A national survey conducted within the Nordic eHealth project NORDeHEALTH provided an important opportunity to advance our understanding of adolescent users of patient portals. The present study explored reasons for reading the EHRs, the perceived usefulness of information and functions in a patient portal, and the association between frequency of use and encouragement to read the EHR.

Methods: Data were collected in a survey using convenience sampling, available through the Swedish online health portal during three weeks in January and February 2022. This study included a subset of items and only respondents aged 15-19. Demographic factors and frequencies on Likert-style questions were reported with descriptive statistics, while Fisher's exact test was used to explore differences in use frequency based on having been encouraged to read by a HCP.

Results: Of 13 008 users who completed the survey, 218 (1.7%) were unique users aged 15-19 (females: 77.1%). One fifth (47/218, 21.6%) had been encouraged by HCPs to read their records, and having been encouraged by HCP was related to higher use frequency (p=.043). All types of information were rated high on usefulness, while some functions were rated low, such as blocking specific clinical notes from HCPs and managing services for family members. The main reason for reading their health records online was out of curiosity.

Conclusions: Adolescents who read their records online perceive it to be useful. Encouragement by HCPs can lead to increased use of patient portals among adolescents. Findings should be considered in the future design of patient portals for adolescents.

Keywords: adolescents; adolescent health; Patient Accessible Electronic Health Record (PAEHR); Electronic Health Record (EHR); patient portal; national survey; ehealth

KEY MESSAGES

What is already known on this topic

- Implementation of online record access for adolescents is controversial
- Citizens in Sweden have had online record access via a national patient portal for a decade
- Prior research has focused on adolescents without experience of using a patient portal, while existing studies of adolescent patient portal users have been limited to contexts of serious illness.
- Adolescents report similar benefits of reading EHRs to those of adults, such as reduced anxiety, enhanced knowledge about their illness, ability to ask informed questions and reflecting more on their health

What this study adds

- Encouragement by HCPs was related to higher use frequency. However, adolescents are rarely encouraged by HCPs to read their notes online.
- Adolescents reading their records perceive all types of information in the EHR as useful.

How this study might affect research, practice or policy

- Enhanced encouragement and support for adolescents will be required to support the transition into adulthood and involve adolescents in their own care.
- EHR vendors designing patient portals and patient portals intended for adolescent use in particular may benefit from considering the types of information and functions that adolescents find useful.

Confidential: For Review Only

INTRODUCTION

An increasing number of healthcare providers are giving patients access to their electronic health records (EHR), including patient information such as clinical notes, diagnoses, and medication lists. The practice of enabling access to free-text notes written in the EHRs by healthcare professionals (HCPs) is sometimes called 'open notes'.[1] Commonly, patients gain online record access (ORA) via secure online portals. Patient-accessible electronic health records (PAEHR) are becoming increasingly expected among patients, even affecting patients' choice of healthcare provider. In particular, adolescents are growing up with expectations of digital convenience that also applies to healthcare.[2]

During early childhood, PAEHR portal systems can often allow for parental proxy access, where parents or guardians of the minor have access to the EHR of the child. However, adolescents needing healthcare face a hidden battle in balancing privacy and personal autonomy with the need for support from parents. Worries about sensitive health information (e.g. mental health issues and sexual health) being exposed to parents through the PAEHR can dissuade young people from accessing medical care.[3-4] The transition from adolescent to adult patienthood has therefore raised concerns among HCPs[5–7] and implementing online access to EHRs is especially controversial in this population.

The e-service for PAEHRs in Sweden is *Journalen*, available via the national web patient portal 1177.se, accessible to patients in all 21 Swedish regions.[8] According to publicly available statistics,[9] Journalen is being accessed by a steadily increasing number of users; approximately 1 360 000 unique users per month during 2022. Adult parent users have automatic access to their child's records in Journalen, but lose this access when the child turns 13, and adolescents gain their record access at 16. When an adolescent is between 13 and 16 years old, parents and adolescents can apply for extended access outside of the default. Adolescent patients access the same information as adults. The content includes for example a list of prescribed medications, lab results, growth curves, vaccinations, diagnoses, maternity care records, referrals, warnings, and treatment plans (Figure 1). However, availability differs according to region and connected health care providers (who have agreed to give access) (Figure 2). For example, psychiatric notes are shown in 17 of 21 regions.[10] Functions such as prescription renewal and appointment booking are conducted on the patient portal, yet externally from the PAEHR.

Figure 1. The patient-accessible electronic health record *Journalen* after log-in (manually translated), showing the functions and information available.[11] Service produced by Inera AB under the auspices of Swedish municipalities and regions.

Figure 2. The information shown in *Journalen* depending on region or health care provider (green squares) during the time of the survey.

Two literature reviews on pediatric patient portals found limited studies in this age group. One reviewed definition and utilization[12] while the other focused on use, views, and experiences among adolescents.[13] While expressing a lack of knowledge of how to use PAEHRs,[2,14] adolescents have stated a desire to receive such information from HCPs.[2,15] In a US study examining EHR access of adolescents' records by accounts divided into self, surrogate, and delegate, there was a significant increase of use during adolescence.[16] Furthermore, HCPs have stated the benefits of guiding adolescent patients to become more involved and activated in their care.[17-18] Research has mostly been observational or focused on adolescents' attitudes towards patient portals, rather than experiences. In observational studies, adolescents demonstrate the use of the same portal features as adults,[19] including lab results,[16,20-21] appointment review,[16,20-21] messaging,[16,20] and medications.[21] Existing studies of adolescent users have been limited to contexts of serious illness, and these adolescents report similar benefits of reading EHRs to those of adults, such as reduced anxiety, enhanced knowledge about their illness, ability to ask informed questions and reflecting more on their health,[22] and empowerment.[5,22] A benefit unique for adolescents concerned a supported transition into adulthood.[22] To date, no studies have systematically examined how adolescents more broadly experience ORA.

Confidential: For Review Only

Study Aim

A recent scoping review concluded that with few exceptions (Australia, Canada, New Zealand, and the United Kingdom), the entire body of literature on adolescent access to EHRs had been carried out in the United States.[13] The present research explored reasons for reading the EHRs, the perceived usefulness of information and functions in a patient portal, and the association between frequency of use and HCP encouragement to read the EHR, among adolescents in Sweden.

METHODS

Study Design

Within the NORDeHEALTH research project,[23] an open survey designed to elicit the opinions and experiences of PAEHR users was conducted. The data were collected for three weeks from January to February 2022. Convenience sampling was used. Participants were notified about the survey when they logged into their records through the Swedish national PAEHR *Journalen*, by receiving a request to voluntarily participate in the survey, along with detailed information about the study. Accessing the survey without logging in was impossible. The participation request did not appear once the patient had completed the survey. Participants provided electronic consent and there was no financial incentive. Only survey respondents between 15-19 years old were included for this paper. Due to an ethical-legal requirement of written parental consent for participants younger than 15 (The Act concerning the Ethical Review of Research Involving Humans[24]), which would complicate survey distribution, we opted to exclude those aged 13 and 14. Therefore, the first survey item after consent concerned age and selecting *14 or younger* triggered information about exclusion and survey termination. The Checklist for Reporting Results of Internet E-Surveys (CHERRIES)[25] was used to report this study.

Data Collection

An anonymous questionnaire covering 83 items was designed in English and translated into Swedish. The questionnaire included a mixture of close- and open-ended questions with various response options (yes/no, multiple choice, and free-text form). The design and selection of items were informed by prior studies.[1,19,26] Answering close-ended questions was mandatory, while free-text questions were optional. The Swedish survey was distributed over 12 pages and some items were conditionally displayed based on responses to other items. Including conditional questions, the number of items ranged between 1-10 item per page. Respondents could move back and forth between survey sections, however did not have an option to review the filled form before submission. For this study, five items were included (see Figure 3) based on the study's aim. Three of these items used a 5-point Likert scale (ranging from disagree to agree), and two were close-ended questions. Data on use frequency was collected to enable comparison across groups. The items used in the current study are found in Figure 3, and answer options and item number can be found in Appendix A (English original and Swedish translation). The full survey and further details about the data collection procedure are found in a separate publication.[27] It should be noted that the availability of information and functions in *Journalen* varies across regions in Sweden, which may affect respondents' assessment. However, the items on perceived usefulness are formulated anticipatory (see Figure 3).

Figure 3. Survey items included in the study.

Statistical Analysis

Only completed questionnaires were analyzed. Aside from using descriptive statistics for calculating percentages for different response options, the Fisher's exact test was used to explore the difference in use frequency based on whether a HCP (physician, nurse, psychologist, physiotherapist, or other medical staff) had encouraged PAEHR use. Non-parametric tests were conducted due to the small sample size, and the significance level was set to 95%. The use frequency response options *10-20 times* and *More than 20 times* were merged for analysis due to a small sample. The SPSS 28 software was used for all analyses, and figures were produced using R.

Confidential: For Review Only

Patient and public involvement

The survey was tested with the public before launch to assess its clarity and response burden, leading to minor adjustments. There was no other patient and public involvement in the design or conduct of the study.

RESULTS

Characteristics of the Participants

During the study period, 23 855 users opened the survey, and 15 867 (66.5%) started filling it in. Of these, 13 010 (82.0%) submitted the survey. Two (2/13 010, 0.02%) were 14 years old or younger and were excluded from the survey. Of the 13 008 respondents who completed the survey, 218 were 15-19 years old (1.7%). Of respondents aged between 15-19, 77.1% (168/218) identified as women, 17.9% (39/218) as men while 5.0% (11/218) selected Other. See data on survey completion in a previous publication[26] and descriptive statistics for all variables in Appendix B.

Experience with Accessing Records Online

Two out of five respondents had logged in more than 20 times (85/218, 39.0%) and one third had logged in 2-9 times (74/218, 33.9%) (see Table 1).

Table 1. Participants' frequency of accessing their health records online.

Frequency of health record access, n (%)	Participants (N=218)
This is my first time	11 (5)
2 to 9 times	74 (33.9)
10-20 times	48 (22)
More than 20 times	85 (39)

Encouragement to Read the Health Records Online

One survey question focused on whether respondents had been encouraged or reminded by anyone or any media source to read their records online. The most common response was *nobody encouraged me* (118/218, 54.1%), see Figure 4. Of four respondents that chose *Other*, one respondent provided a free-text description, citing "myself". About a fifth (48/218, 22.0%) had been encouraged by some type of HCP, and Fisher's exact test revealed that those encouraged by a HCP were more frequent users of the patient portal than those who had not (p=.043).

Figure 4. Encouragement to read health records online. Because respondents could select multiple items, the total number can exceed 100%. Legend: *Types of HCPs.

Reasons for Reading the Health Records Online

Four reasons were chosen by more than half of respondents: *out of general curiosity* (170/218, 78.0%), *to get an overview of one's medical history and/or treatment* (159/218, 72.9%), *to ensure having understood what the physician or nurse said* (149/218, 68.3%), and *to improve one's understanding for one's health issue* (138/218, 63.3%) (see Figure 5). Of 50 respondents who selected *Other*, 16 provided free-text comments. The comments included ensuring accuracy, memory reinforcement, poor notification frequency (i.e. logging in to check for new information due to lack of notifications), specification of sought information (e.g., test results, Covid-19 vaccinations), and retrieving a record copy for monetary activity compensation.

Figure 5. Adolescents' reasons for reading health records online.

Usefulness of Portal Information and Function

All information types were considered useful by more than 3/4 of participants, see Figure 6.

Figure 6. Adolescents' perceived usefulness of different types of patient portal information.

Confidential: For Review Only

Perceived usefulness of various functions was also examined, most of which do not currently exist in *Journalen*. Most functions were perceived as useful by a majority of respondents, see Figure 7. Yet, four functions were considered useful by less than half of respondents: *accessing information and managing services for children* and for *family members*, *contributing information about expectations for the health care visit*, and *blocking specific clinical notes from certain HCPs/medical staff*. Of 24 participants who wrote a comment under *Other*, 7 referred to a specific function. Comments concerned the ability to view radiology images (median=4.5), diagnosis (median=3.5), dentistry records (median=5), ability to read children's records when under 18 (median=5), records from childhood (median=5), and to view appointments from gynecologic care (median=5). Other free-text answers were non-responses or unspecified. *Medical certificates and other certificates* concerned certificates from HCPs, such as COVID vaccination and for insurance and sick leave.

Figure 7. Adolescents' perceived usefulness of patient portal functions.

DISCUSSION

In our national survey of PAEHR experiences in Sweden, adolescents perceived all proposed clinical information as consistently useful. Ratings for portal functions varied. Common reasons for reading records were curiosity and to get an overview of medical history. Sharing clinical information was the least common reason for accessing the record. Encouragement by HCPs to read records was associated with higher use. The reasons for reading records align with previous research on adults[19] and US adolescents.[22]

While all information types were rated highly on usefulness, test results were considered the most useful. This result accords with earlier research on adolescents,[16,20-21] but also with research on parents[28-29] and general adult populations.[19] Unsurprisingly, adolescents rated the ability to manage family members or children low. The function perceived as the third most useful was the ability to order and manage various certificates. This was likely associated with the timing of survey distribution, as the ongoing COVID-19 pandemic required Swedish citizens to present COVID-19 certificates for traveling and attending events. The boost of PAEHR use as a result of the COVID-19 pandemic has been mentioned previously.[30] Related to this, it is somewhat unexpected that immunizations were rated among the least useful types of information. However, Swedish regions differed in managing COVID-19 vaccination through the national e-service or other apps, and not all regions displayed information on vaccinations. Another finding was that adolescents do not read their PAEHRs to share information. This finding is consistent with a previous survey study based in Canada, where adolescents without access to their records anticipated not being likely to share information from the records with others.[31]

Encouragement from HCPs was linked to higher use, indicating the influence of social factors in eHealth adoption.[32] Another possible explanation is that those with more active medical issues needed to review their health record more frequently. Encouragement from HCPs and family/friends has increased PAEHR use in adults,[33] suggesting that this may be an important factor influencing adoption and use also in adolescents. Lack of HCP encouragement aligns with research on adults in the United States[34] and Sweden,[35] and may stem from concerns about medical literacy, increased workload, and uncertainty about visible information.[2,5,36–38] Not encouraging adolescents may lead to a missed opportunity for early patient involvement.[17] Increased PAEHR usage among adolescents can motivate policy-making stakeholders and EHR vendors to improve patient portals for this population.[5]

This study has limitations. Response biases in self-report surveys are unknown. Survey availability on the national patient portal may have influenced respondents' decisions based on their competence and skills in using the service, potentially limiting the generalizability of the findings and causing positive bias as most adolescent respondents were frequent users. However, we opted to use convenience sampling as part of an exploratory approach. The majority of participants were women, which is consistent with adult and previous adolescent PAEHR users but represents a strong gender imbalance. Only 1.7% of the total respondents were aged 15-19, possibly due to younger individuals being less affected by health conditions and less engaged in personal health management. The survey

Confidential: For Review Only

was also designed for adults, and adolescents may have struggled to understand some questions. Furthermore, the reliability and validity of the survey utilized in this study have not been formally evaluated. While the survey was designed to capture the intended data, a comprehensive assessment of its psychometric properties was beyond the scope of this research. Still, the survey was tested with the public, as stated under Patient and public involvement.

The present results provide a broader understanding of adolescents reading their EHRs, however further work is required to establish the viability of the findings in other settings. Several questions remain, relating to for example eHealth literacy and confidentiality. It should be noted that the current study included only a subset of items from a larger survey, and there is an intention to publish additional findings on the adolescent population, on participants' views about security and privacy. Regarding our research methodology, we offer a few suggestions for future work. First, frequency of use was defined by how many times (first time, 2-5 times etc.) the users accessed their records in the past 12 months. Another approach is to ask how often users access their records (daily, weekly, etc.), which allows for an understanding of the visit regularity. Second, further research may benefit from providing a clearer distinction between a specific PAEHR service and patients' ORA more broadly. Lastly, including a larger and more evenly gender-distributed sample and specific patient groups with chronic diseases could furnish greater insights into adolescent users' experiences.

Data availability statement

Data are available from the corresponding author on reasonable request.

Ethics statements

Patient consent for publication

Electronic consent obtained from survey respondents.

Ethics approval

Ethical approval for this study was obtained from the Regional Ethical Review Board in Uppsala, Sweden (EPN 2021/05229). All respondents received study information and provided informed consent.

Abbreviations

EHR: electronic health record

HCP: healthcare professional

ORA: online record access

PAEHR: patient-accessible electronic health record

FOOTNOTES

Contributors: JH wrote the manuscript, created the tables, and designed the figures. MH, CB, IS, JM, ÅC, and HR contributed to the study design. All authors read the manuscript, provided feedback, and approved the final draft for submission.

Funding: This work was supported by NordForsk through the funding to Nordic eHealth for Patients: Benchmarking and Developing for the Future, NORDeHEALTH, (Project #100477), the Swedish Research Council for Health, Working Life and Welfare (FORTE) through the funding to Beyond Implementation of eHealth (Project #2020-01229).

Competing interests: None.

Patient and public involvement: Patients and/or the public were involved in survey testing but otherwise not involved in the design, or conduct, or reporting, or dissemination plans of this research.

Provenance and peer review: Not commissioned; externally peer reviewed.

Confidential: For Review Only

REFERENCES

1. Walker J, Leveille S, Bell S, et al. OpenNotes After 7 Years: Patient Experiences With Ongoing Access to Their Clinicians' Outpatient Visit Notes. J Med Internet Res. 2019;21(5):e13876. doi:10.2196/13876
2. Beaton L, Williams I, Sanci L. Exploring adolescent and clinician perspectives on Australia. Aust J Prim Health. Published online March 15, 2021. doi:10.1071/PY20169
3. Bayer R, Santelli J, Klitzman R. New Challenges for Electronic Health Records: Confidentiality and Access to Sensitive Health Information About Parents and Adolescents. JAMA. 2015;313(1):29. doi:10.1001/jama.2014.15391
4. Ford CA. Foregone Health Care Among Adolescents. JAMA. 1999;282(23):2227. doi:10.1001/jama.282.23.2227
5. Carlson JL, Goldstein R, Buhr T, Buhr N. Teenager, parent, and clinician perspectives on the electronic health record. Pediatrics. 2020;145(3). doi:10.1542/peds.2019-0193
6. Goldstein RL, Anoshiravani A, Svetaz MV, Carlson JL. Providers' Perspectives on Adolescent Confidentiality and the Electronic Health Record: A State of Transition. J Adolesc Health. 2020;66:296-300. doi:10.1016/j.jadohealth.2019.09.020
7. Stablein T, Loud KJ, DiCapua C, Anthony DL. The Catch to Confidentiality: The Use of Electronic Health Records in Adolescent Health Care. J Adolesc Health. 2018;62(5):577-582. doi:10.1016/j.jadohealth.2017.11.296
8. Hägglund M, Scandurra I. Patients' Online Access to Electronic Health Records: Current Status and Experiences from the Implementation in Sweden. MEDINFO 2017 Precis Healthc Inform. Published online 2017:723-727. doi:10.3233/978-1-61499-830-3-723
9. Statistik för Ineras tjänster [Statistics for Inera's services]. Inera AB. Accessed August 25, 2023. https://www.inera.se/tjanster/statistik-for-ineras-tjanster/
10. Bärkås A, Scandurra I, Rexhepi H, Blease C, Cajander Å, Hägglund M. Patients' Access to Their Psychiatric Notes: Current Policies and Practices in Sweden. Int J Environ Res Public Health. 2021;18(17):9140. doi:10.3390/ijerph18179140
11. Journalen. Inera. Published December 2022. Accessed December 8, 2022. https://journalen.1177.se/
12. Bush RA, Connelly CD, Fuller M, Pérez A. Implementation of the Integrated Electronic Patient Portal in the Pediatric Population: A Systematic Review. Telemed J E-Health Off J Am Telemed Assoc. 2016;22(2):144-152. doi:10.1089/tmj.2015.0033
13. Hagström J, Blease C, Haage B, Scandurra I, Hansson S, Hägglund M. Use, Views of and Experiences of Web-Based Access to Electronic Health Records for Children, Adolescents, and Parents: Scoping Review. J Med Internet Res. Published online 2022. doi:10.2196/40328
14. Miklin DJ, Vangara SS, Delamater AM, Goodman KW. Understanding of and Barriers to Electronic Health Record Patient Portal Access in a Culturally Diverse Pediatric Population. JMIR Med Inform. 2019;7(2):e11570. doi:10.2196/11570
15. Bergman DA, Brown NL, Wilson S. Teen Use of a Patient Portal: A Qualitative Study of Parent and Teen Attitudes. Perspect Health Inf Manag. 2008;5:13.
16. Steitz BD, Cronin RM, Davis SE, Yan E, Jackson GP. Long-term patterns of patient portal use for pediatric patients at an academic medical center. Appl Clin Inform. 2017;8(3):779-793. doi:10.4338/ACI-2017-01-RA-0005
17. Bourgeois FC, DesRoches CM, Bell SK. Ethical Challenges Raised by OpenNotes for Pediatric and Adolescent Patients. Pediatrics. 2018;141(6):e20172745. doi:10.1542/peds.2017-2745
18. Lam BD, Bourgeois F, DesRoches CM, Dong Z, Bell SK. Attitudes, experiences, and safety behaviours of adolescents and young adults who read visit notes: Opportunities to engage patients early in their care. Future Heal J. 2021;8(3):e585-e592. doi:10.7861/fhj.2021-0118

Confidential: For Review Only

19. Moll J, Rexhepi H, Cajander Å, et al. Patients' Experiences of Accessing Their Electronic Health Records: National Patient Survey in Sweden. J Med Internet Res. 2018;20(11):e9492. doi:10.2196/jmir.9492
20. Calman N, Pfister HR, Lesnewski R, Hauser D, Shroff N. Electronic Access to Adolescents' Health Records: Legal, Policy, and Practice Implications. Vol 22.; 2014:11-14. https://www.aafp.org/fpm/2015/0300/p11.html
21. Thompson LA, Martinko T, Budd P, Mercado R, Schentrup AM. Meaningful use of a confidential adolescent patient portal. J Adolesc Health. 2016;58(2):134-140. doi:10.1016/j.jadohealth.2015.10.015
22. Hong MK, Wilcox L, Feustel C, Wasileski-Masker K, Olson TA, Simoneaux SF. Adolescent and Caregiver use of a Tethered Personal Health Record System. AMIA Annu Symp Proc AMIA Symp. 2016;2016:628-637.
23. Hägglund M. Nordic countries lead new initiative on patient access to EHRs. TheBMJopinion. Published May 18, 2021. Accessed January 16, 2022. https://blogs.bmj.com/bmj/2021/05/18/maria-hagglund-nordic-countries-lead-new-initiative-on-patient-access-to-ehrs/
24. Riksdagsförvaltningen. 2003:460.; 2003. Accessed August 3, 2022. https://www.riksdagen.se/sv/dokument-lagar/dokument/svensk-forfattningssamling/lag-2003460-om-etikprovning-av-forskning-som_sfs-2003-460
25. Eysenbach G. Improving the Quality of Web Surveys: The Checklist for Reporting Results of Internet E-Surveys (CHERRIES). J Med Internet Res. 2004;6(3):e34. doi:10.2196/jmir.6.3.e34
26. Zanaboni P, Kummervold PE, Sørensen T, Johansen MA. Patient use and experience with online access to electronic health records in Norway: Results from an online survey. J Med Internet Res. 2020;22(2). doi:10.2196/16144
27. Hägglund M, Kharko A, Hagström J, et al. The NORDeHEALTH 2022 Patient Survey: A cross-sectional survey of national patient portal users in Norway, Sweden, Finland, and Estonia. J Med Internet Res. 2023;25:e47573. doi:10.2196/47573
28. Ahlers-Schmidt CR, Nguyen M. Parent Intention to Use a Patient Portal as Related to Their Children Following a Facilitated Demonstration. Telemed E-Health. 2013;19(12):979-981. doi:10.1089/tmj.2013.0041
29. Thompson LA, Black EW, Saliba H, Schentrup AM. Parents' knowledge of and opinions about healthcare laws and technology in primary care. Inform Prim Care. 2012;20(1):69-74. doi:10.14236/jhi.v20i1.49
30. Blease C, Salmi L, Hägglund M, Wachenheim D, DesRoches C. COVID-19 and Open Notes: A New Method to Enhance Patient Safety and Trust. JMIR Ment Health. 2021;8(6):e29314. doi:10.2196/29314
31. Frazer C, Ratchford F, Roch J. Enabling Adolescent Electronic Access to Personal Health Information. Stud Health Technol Inform. 2017;234:115-119.
32. Venkatesh V, Morris MG, Davis GB, Davis FD. User Acceptance of Information Technology: Toward a Unified View. MIS Q. 2003;27(3):425-478. doi:10.2307/30036540
33. Mukhopadhyay S, Basak R, Carpenter D, Reithel BJ. Patient use of online medical records: an application of technology acceptance framework. Inf Comput Secur. 2019;28(1):97-115. doi:10.1108/ICS-07-2019-0076
34. DesRoches CM, Leveille S, Bell SK, et al. The Views and Experiences of Clinicians Sharing Medical Record Notes With Patients. JAMA Netw Open. 2020;3(3):e201753. doi:10.1001/jamanetworkopen.2020.1753
35. Rexhepi H, Moll J, Huvila I. Online electronic healthcare records: Comparing the views of cancer patients and others. Health Informatics J. 2020;26(4):2915-2929. doi:10.1177/1460458220944727
36. Hagström J, Blease C, Haage B, Scandurra I, Hansson S, Hägglund M. Views, Use, and Experiences of Web-Based Access to Pediatric Electronic Health Records for Children, Adolescents, and Parents: Scoping Review. J Med Internet Res. 2022;24(11):e40328. doi:10.2196/40328

Confidential: For Review Only

37. Grünloh C, Myreteg G, Cajander Å, Rexhepi H. “Why Do They Need to Check Me?” Patient Participation Through eHealth and the Doctor-Patient Relationship: Qualitative Study. J Med Internet Res. 2018;20(1):e8444. doi:10.2196/JMIR.8444
38. Petersson J, Backman C. Patient-accessible online health records: Reconfigurations of clinical rhythms and doctors’ front- and backstage spaces. Soc Sci Med. 2022;292:114635. doi:10.1016/j.socscimed.2021.114635

Confidential: For Review Only

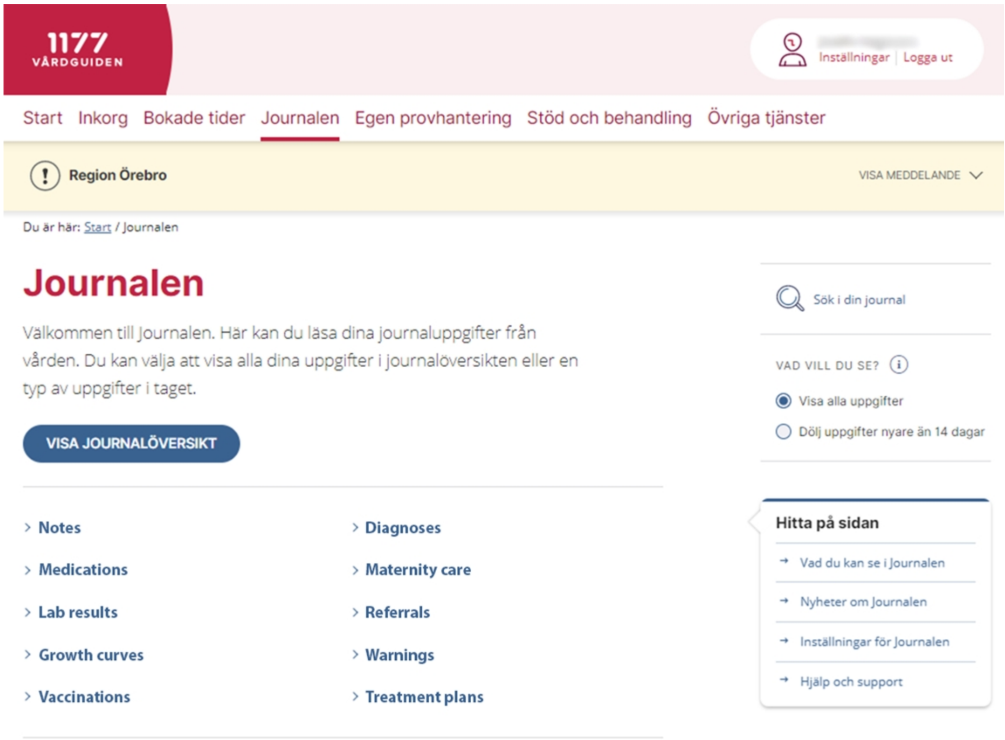

Figure 1. The patient-accessible electronic health record Journalen after log-in (manually translated), showing the functions and information available.[11] Service produced by Inera AB under the auspices of Swedish county councils and regions.

578x427mm (300 x 300 DPI)

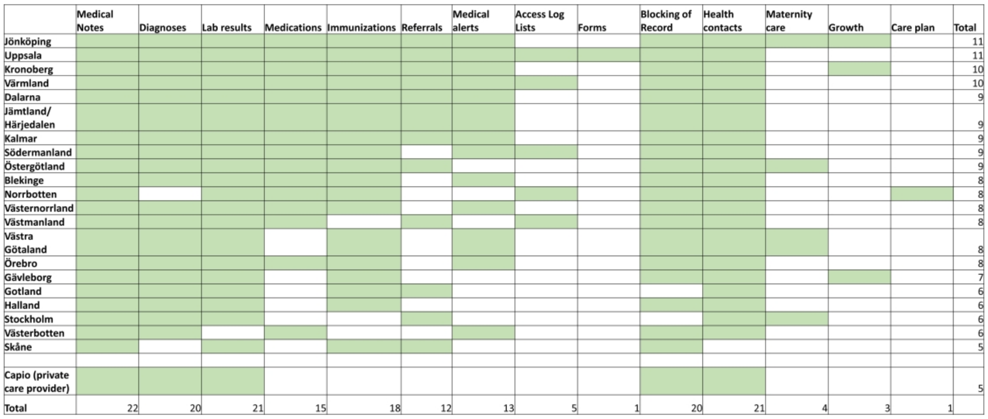

Figure 2. The information shown in Journalen depending on region or health care provider (green squares) during the time of the survey.

758x320mm (300 x 300 DPI)

1. How often do you think that you have accessed your health record during the last 12 months?
2. Did any of the following encourage or remind you to read your health record?
   (check all that apply)
3. Please indicate how much you disagree or agree with the following statement:
   I read my health record online… [reasons for reading]
4. How useful would it be to have access to the following information in the portal?
5. How useful would it be to have access to the following functions in the portal?

Figure 3. Survey questions included in the study.

520x176mm (300 x 300 DPI)

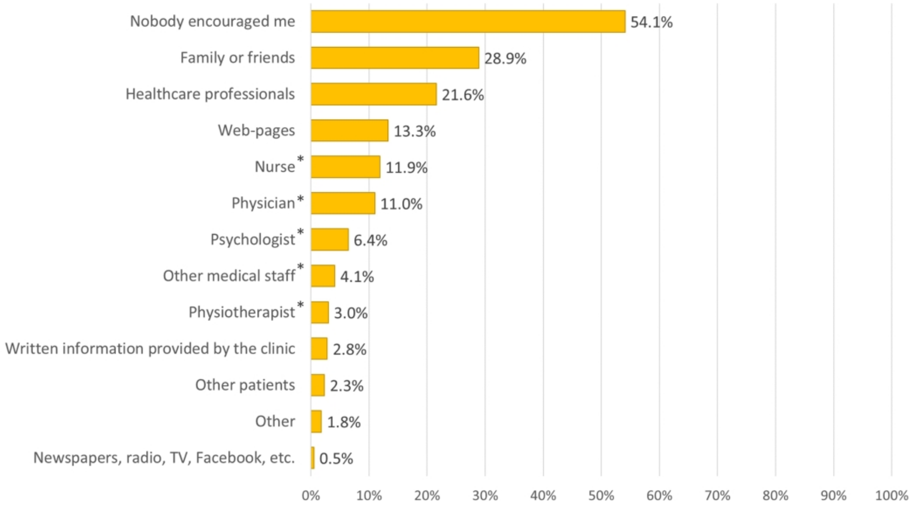

Figure 4. Encouragement to read health records online. Because respondents could select yes for multiple items, the total number can exceed 100%. Legend: *Types of HCPs.

478x250mm (300 x 300 DPI)

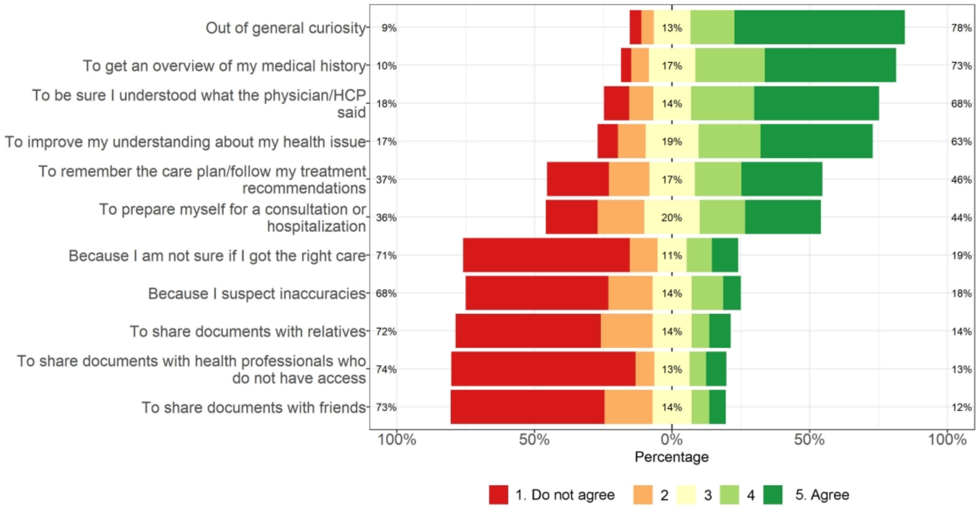

Figure 5. Adolescents’ reasons for reading health records online.

292x152mm (300 x 300 DPI)

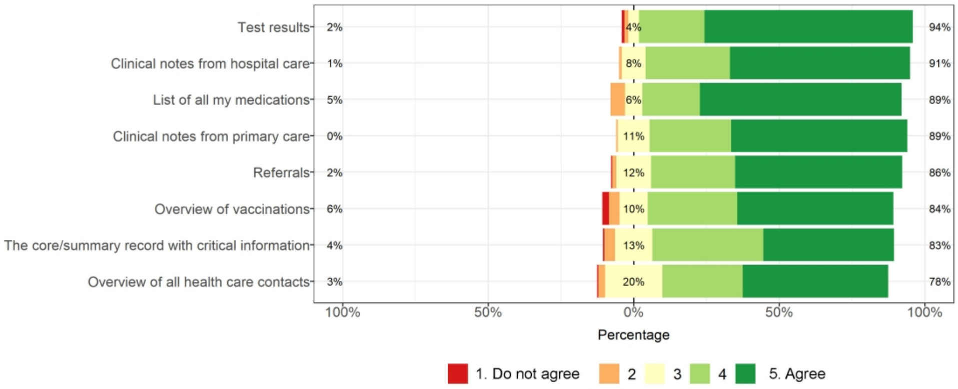

Figure 6. Adolescents' perceived usefulness of different types of patient portal information.

279x112mm (300 x 300 DPI)

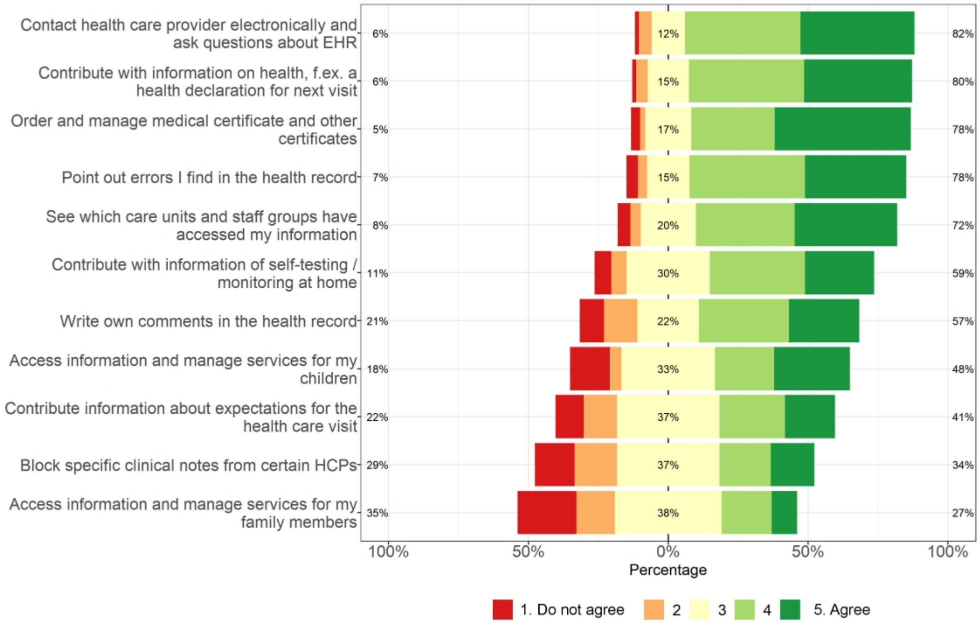

Figure 7. Adolescents' perceived usefulness of patient portal functions. Legend: *Functions that exist in all or some regions in Sweden. Only adult users over 18 years old have access to their children's records.

279x177mm (300 x 300 DPI)

Appendix A

Table 1. Original survey created in English

Number in survey	Question	Response alternatives
6	**How often do you think that you have accessed your health record during the last 12 months?**	1 - This is my first time<br>2 - 2 to 9 times<br>3 - 10 to 20 times<br>4 - More than 20 times
7	**Did any of the following encourage or remind you to read your health record?**	HCPs: Physician<br>HCPs: Nurse<br>HCPs: Psychologist<br>HCPs: Physiotherapist<br>HCPs: Other medical staff<br>Written information provided by the hospital/healthcare facility /clinic<br>Family or friends<br>Web-pages, such as the National health portal web-page, etc.<br>Newspapers, radio, TV, Facebook, etc.<br>Other patients<br>Nobody encouraged me<br>Other
8	Please indicate how much you disagree or agree with the following statement:<br>**I read my health record online…**	1 - Disagree,<br>2, 3, 4,<br>5 - Agree
		out of general curiosity
		to improve my understanding about my health issue
		to prepare myself for a consultation or hospitalization
		to get an overview of my medical history and/or treatment
		to be sure I understood what the physician/healthcare professional said
		to remember the care plan/follow my treatment recommendations
		because I suspect inaccuracies
		to share documents with relatives
		to share documents with friends
		to share documents with health professionals who do not have access
		because I am not sure if I got the right care
		Other, please specify

38	**How useful would it be to have access to the following information in the portal?**		1 - Not useful at all<br>2 - Not very useful<br>3 - Neither useful nor useless<br>4 - Useful<br>5 - Very useful
		The core/summary record with critical information	
		Referrals (content and how it is handled in care)	
		List of all pharmaceuticals/my medications	
		Overview of vaccinations	
		Results of tests	
		Clinical notes from primary care	
		Clinical notes from hospital care	
		Overview of all health care contacts	
39	**How useful would it be to have access to the following functions in the portal?**		1 - Not useful at all<br>2 - Not very useful<br>3 - Neither useful nor useless<br>4 - Useful<br>5 - Very useful
		Ability to access information and manage services for my children	
		Ability to access information and manage services for my family members	
		See which care units and staff groups have accessed my information (see log data)	
		Ability to block certain medical records from access by other medical staff	
		Ability to contact health care provider electronically and ask questions about medical record	
		Ability to point out errors I find in the health record	
		Ability to write own comments to text in the health record	
		Contribute with information on health, for example by providing a health declaration for next visit	
		Contribute with information of self-testing/monitoring at home	
		Contribute information about expectations for the health care visit	
		Ability to order and manage medical certificate and other certificates	
		Other	

Table B2. Swedish translation of survey

Number in survey	Question	Response alternatives
6	**Hur ofta har du läst i din journal under de senaste 12 månaderna?**	1 - This is my first time<br>2 - 2 to 9 times<br>3 - 10 to 20 timesa<br>4 - More than 20 times
7	**Har du blivit uppmuntrad eller påmind att läsa din journal av någon/något av följande:**	Vårdpersonal: Läkare<br>Vårdpersonal: Sjuksköterska<br>Vårdpersonal: Psykolog<br>Vårdpersonal: Fysioterapeut/sjukgymnast<br>Vårdpersonal: Annan vårdpersonal<br>Skriftlig information från sjukhuset/vårdinrättning/klinik<br>Familj eller vänner<br>Webbsidor, såsom 1177.se Vårdguiden, etc.<br>Tidningar, radio, TV, Facebook, etc.<br>Andra patienter<br>Ingen har uppmuntrat eller påmint mig<br>Annat
8	Markera i vilken utsträckning du instämmer med följande påståenden:<br>**Jag läser min journal på internet…**	1 - Instämmer inte, 2, 3, 4,<br>5 - Instämmer
	Av nyfikenhet	
	För att förbättra min förståelse för mitt hälsotillstånd	
	För att förbereda mig själv för ett vårdbesök eller sjukhusvistelse	
	För att få en överblick av min hälsohistoria och/eller bakgrund	
	För att försäkra mig om att jag förstått vad läkaren/vårdpersonalen sagt	
	För att komma ihåg vårdplanen/följa mina behandlingsrekommendationer	
	För att jag misstänker felaktigheter	
	För att dela med mig av information till släkt	
	För att dela med mig av information till vänner	
	För att dela med mig av information till vårdpersonal som inte har tillgång till min journal	
	För att jag är osäker på om jag fått rätt vård	
	Annat, specificera gärna	

38		**Hur användbart skulle det vara för dig att ha tillgång till följande information i Journalen?**	1 - Inte alls användbart<br>2 - Inte särskilt användbart<br>3 - Varken eller<br>4 - Användbart<br>5 - Väldigt användbart
		En sammanfattning av journalen med viktig information	
		Remisser (innehåll och status)	
		Lista över mina läkemedel/mediciner	
		Vaccinationer	
		Provsvar	
		Journalanteckningar från primärvården	
		Journalanteckningar från specialistvården	
		Överblick av samtliga kontakter med vården	
39		**Hur användbart skulle det vara för dig att ha tillgång till följande funktioner i Journalen?**	1 - Inte alls användbart<br>2 - Inte särskilt användbart<br>3 - Varken eller<br>4 - Användbart<br>5 - Väldigt användbart
		Möjlighet att ta del av information och hantera tjänster för mina barn	
		Möjlighet att ta del av information och hantera tjänster för andra familjemedlemmar	
		Möjlighet att kunna blockera specifika journalanteckningar för viss vårdpersonal	
		Se vilka vårdinrättningar och personal som har läst i min journal (loggar)	
		Möjlighet att kunna kontakta vårdpersonal online för att ställa frågor om min journal	
		Möjlighet att påpeka felaktigheter i journalen	
		Möjlighet att skriva egna kommentarer i journalen	
		Möjlighet att bidra med information om min hälsa, t ex genom att fylla i en hälsodeklaration, inför ett besök	
		Möjlighet att bidra med information från självtest/monitorering hemma	
		Möjlighet att bidra med information om mina förväntningar på vårdbesöket	
		Möjlighet att beställa och hantera sjukskrivning, medicinsk intyg och andra dokument (vaccinationsintyg t ex)	
		Annat	

Appendix B

Detailed results from the survey questions chosen for this study.

Encouragement to Read.

Did any of the following encourage or remind you to read your health record? n (%)	Frequency
HCPs: Physician	24 (11.0)
HCPs: Nurse	26 (11.9)
HCPs: Psychologist	14 (6.4)
HCPs: Physiotherapist	6 (2.8)
HCPs: Other medical staff	9 (4.1)
Written information provided by the hospital/healthcare facility/clinic	12 (5.5)
Family or friends	63 (28.9)
Web-pages, such as the National health portal web-page, etc.	29 (13.3)
Newspapers, radio, TV, Facebook, etc.	1 (0.5)
Other patients	5 (2.3)
Nobody encouraged me	118 (54.1)
Other	4 (1.8)

Reasons for reading health records online.

**I read my health record online…, n** (%)	1. Disagree	2	3	4	5. Agree
… out of general curiosity	9 (4.1)	10 (4.6)	29 (13.3)	35 (16.1)	135 (61.9)
… to improve my understanding about my health issue	16 (7.3)	22 (10.1)	42 (19.3)	49 (22.5)	89 (40.8)
… to prepare myself for a consultation or hospitalization	41 (18.8)	37 (17.0)	44 (20.2)	36 (16.5)	60 (27.5)
… to get an overview of my medical history and/or treatment	8 (3.7)	14 (6.4)	37 (17.0)	55 (25.2)	104 (47.7)
… to be sure I understood what the physician/healthcare professional said	20 (9.2)	19 (8.7)	30 (13.8)	50 (22.9)	99 (45.4)
… to remember the care plan/follow my treatment recommendations	49 (22.5)	32 (14.7)	36 (16.5)	37 (17.0)	64 (29.4)
… because I suspect inaccuracies	113 (51.8)	35 (16.1)	31 (14.2)	25 (11.5)	14 (6.4)
… to share documents with relatives	115 (52.8)	41 (18.8)	31 (14.2)	14 (6.4)	17 (7.8)
… to share documents with friends	122 (56.0)	38 (17.4)	31 (14.2)	14 (6.4)	13 (6.0)
… to share documents with healthcare professionals who do not have access	146 (67.0)	15 (6.9)	28 (12.8)	13 (6.0)	16 (7.3)
… because I am not sure if I got the right care	132 (60.6)	22 (10.1)	23 (10.6)	20 (9.2)	21 (9.6)
Other	168 (77.1)	20 (9.2)	5 (2.3)	3 (1.4)	22 (10.1)

Perceived usefulness of various types of information.

Information	Not useful at all	Not very useful	Neither useful nor useless	Useful	Very useful
The core/summary record with critical information	1 (0.5)	8 (3.7)	28 (12.8)	83 (38.1)	98 (45.0)
Referrals (content and how it is handled in care)	1 (0.5)	3 (1.4)	26 (11.9)	63 (28.9)	125 (11.9)
List of all pharmaceuticals / my medications	0 (0)	11 (5.0)	13 (6.0)	43 (19.7)	151 (69.3)
Overview of vaccinations	5 (2.3)	8 (3.7)	21 (9.6)	67 (30.7)	117 (53.7)
Results of tests (not available in all regions)	2 (0.9)	3 (1.4)	8 (3.7)	49 (22.5)	156 (71.6)
Clinical notes from primary care	0 (0)	1 (0.5)	24 (11.0)	61 (28.0)	132 (60.6)
Clinical notes from hospital care	0 (0)	2 (0.9)	18 (8.3)	63 (28.9)	135 (61.9)
Overview of all health care contacts	1 (0.5)	5 (2.3)	43 (19.7)	60 (27.5)	109 (50)

Perceived usefulness of various functions.

Function	Not useful at all	Not very useful	Neither useful nor useless	Useful	Very useful
Ability to access information and manage services for my children (not available in all regions).	31 (14.2)	9 (4.1)	73 (33.5)	46 (21.1)	59 (27.1)
Ability to access information and manage services for my family members.	46 (21.1)	30 (13.8)	83 (38.1)	39 (17.9)	20 (9.2)
Ability to block specific clinical notes from certain healthcare professionals/medical staff.	31 (14.2)	33 (15.1)	80 (36.7)	40 (18.3)	34 (15.6)
See which care units and staff groups have accessed my information (see log data).	10 (4.6)	8 (3.7)	43 (19.7)	77 (35.3)	80 (36.7)
Ability to contact health care provider electronically and ask questions about medical record.	3 (1.4)	10 (4.6)	26 (11.9)	90 (41.3)	89 (40.8)
Ability to point out errors I find in the health record.	9 (4.1)	7 (3.2)	33 (15.1)	90 (41.3)	79 (36.2)
Ability to write own comments to text in the health record.	19 (8.7)	26 (11.9)	48 (22.0)	70 (32.1)	55 (25.2)
Contribute with information on health, for example by providing a health declaration for next visit.	3 (1.4)	9 (4.1)	32 (14.7)	90 (41.3)	84 (38.5)
Contribute with information of self-testing/monitoring at home.	13 (6.0)	12 (5.5)	65 (29.8)	74 (33.9)	54 (24.8)
Contribute information about expectations for the health care visit.	22 (10.1)	26 (11.9)	80 (36.7)	51 (23.4)	39 (17.9)
Ability to order and manage medical certificate and other certificates.	7 (3.2)	4 (1.8)	36 (16.5)	65 (29.8)	106 (48.6)

BMJ Paediatrics Open

Adolescents' reasons for accessing their health records online, perceived usefulness, and experienced provider encouragement: A national survey in Sweden

Journal:	*BMJ Paediatrics Open*
Manuscript ID	bmjpo-2023-002258.R2
Article Type:	Original research
Date Submitted by the Author:	23-Jan-2024
Complete List of Authors:	Hagström, Josefin; Uppsala Universitet, Department of Women's and Children's Health; Uppsala University Hospital, Medtech Science & Innovation Centre<br>Blease, Charlotte; Uppsala Universitet, Department of Women's and Children's Health; Harvard Medical School, Beth Israel Deaconess Medical Center<br>Scandurra, Isabella; Örebro University, School of Business<br>Moll, Jonas; Örebro University, School of Business<br>Cajander, Åsa; Uppsala Universitet, Department of Information Technology<br>Rexhepi, Hanife; University of Skövde, School of Informatics<br>Hägglund, Maria; Uppsala Universitet, Department of Women's and Children's Health; Uppsala University Hospital, Medtech Science & Innovation Centre
Keywords:	Adolescent Health, Health services research, Data Collection

SCHOLARONE™
Manuscripts

I, the Submitting Author has the right to grant and does grant on behalf of all authors of the Work (as defined in the below author licence), an exclusive licence and/or a non-exclusive licence for contributions from authors who are: i) UK Crown employees; ii) where BMJ has agreed a CC-BY licence shall apply, and/or iii) in accordance with the terms applicable for US Federal Government officers or employees acting as part of their official duties; on a worldwide, perpetual, irrevocable, royalty-free basis to BMJ Publishing Group Ltd ("BMJ") its licensees and where the relevant Journal is co-owned by BMJ to the co-owners of the Journal, to publish the Work in this journal and any other BMJ products and to exploit all rights, as set out in our licence.

The Submitting Author accepts and understands that any supply made under these terms is made by BMJ to the Submitting Author unless you are acting as an employee on behalf of your employer or a postgraduate student of an affiliated institution which is paying any applicable article publishing charge ("APC") for Open Access articles. Where the Submitting Author wishes to make the Work available on an Open Access basis (and intends to pay the relevant APC), the terms of reuse of such Open Access shall be governed by a Creative Commons licence – details of these licences and which Creative Commons licence will apply to this Work are set out in our licence referred to above.

Other than as permitted in any relevant BMJ Author's Self Archiving Policies, I confirm this Work has not been accepted for publication elsewhere, is not being considered for publication elsewhere and does not duplicate material already published. I confirm all authors consent to publication of this Work and authorise the granting of this licence.

Confidential: For Review Only

TITLE PAGE

Article title: Adolescents' reasons for accessing their health records online, perceived usefulness, and experienced provider encouragement: A national survey in Sweden

Author information:
Corresponding author:
Josefin Hagström, MSc[1,2] (ORCID: 0000-0003-2835-0259)
Department of Women's and Children's Health, Uppsala University, Uppsala, Sweden

josefin.hagstrom@uu.se
+46184715080

Co-authors:
Charlotte Blease PhD[1,3] (ORCID: 0000-0002-0205-1165)
Isabella Scandurra PhD[4] (ORCID: 0000-0002-2597-1079)
Jonas Moll PhD[4] (ORCID: 0000-0002-4772-4730)
Åsa Cajander PhD[5] (ORCID: 0000-0001-7472-2215)
Hanife Rexhepi PhD[6] (ORCID: 0000-0001-8957-9853)
Maria Hägglund PhD[1,2] (ORCID: 0000-0002-6839-3651)

[1] Department of Women's and Children's Health, Uppsala University, Uppsala, Sweden
[2] Medtech Science & Innovation Centre, Uppsala University Hospital, Uppsala, Sweden
[3] Beth Israel Deaconess Medical Center, Harvard Medical School, Boston, MA, USA
[4] School of Business, Örebro University, Örebro, Sweden
[5] Department of Information Technology, Uppsala University, Uppsala, Sweden
[6] School of Informatics, University of Skövde, Skövde, Sweden

Disclaimers: The views expressed in the submitted article are his or her own and not an official position of the institution or funder.

Source(s) of support: This work was supported by NordForsk (grant number #100477) and the Swedish Research Council for Health, Working Life and Welfare (FORTE) (grant number #2020-01229).

Word count: 2522

Number of figures: 7

Number of tables: 2

Key words: adolescents; adolescent health; Patient Accessible Electronic Health Record (PAEHR); Electronic Health Record (EHR); patient portal; national survey; ehealth

References: 39

Disclosure of relationships and activities: There are no conflicts of interest to declare.

Confidential: For Review Only

ABSTRACT

Background: Having online access to electronic health records (EHRs) may help patients become engaged in their care at an early age. However, little is known about adolescents using patient portals. A national survey conducted within the Nordic eHealth project NORDeHEALTH provided an important opportunity to advance our understanding of adolescent users of patient portals. The present study explored reasons for reading the EHRs, the perceived usefulness of information and functions in a patient portal, and the association between frequency of use and encouragement to read the EHR.
Methods: Data were collected in a survey using convenience sampling, available through the Swedish online health portal during three weeks in January and February 2022. This study included a subset of items and only respondents aged 15-19. Demographic factors and frequencies on Likert-style questions were reported with descriptive statistics, while Fisher's exact test was used to explore differences in use frequency based on having been encouraged to read by a HCP.
Results: Of 13 008 users who completed the survey, 218 (1.7%) were unique users aged 15-19 (females: 77.1%). One fifth (47/218, 21.6%) had been encouraged by HCPs to read their records, and having been encouraged by HCP was related to higher use frequency (p=.043). All types of information were rated high on usefulness, while some functions were rated low, such as blocking specific clinical notes from HCPs and managing services for family members. The main reason for reading their health records online was out of curiosity.
Conclusions: Adolescents who read their records online perceive it to be useful. Encouragement by HCPs can lead to increased use of patient portals among adolescents. Findings should be considered in the future design of patient portals for adolescents.

Keywords: adolescents; adolescent health; Patient Accessible Electronic Health Record (PAEHR); Electronic Health Record (EHR); patient portal; national survey; ehealth

KEY MESSAGES

What is already known on this topic

- Implementation of online record access for adolescents is controversial
- Citizens in Sweden have had online record access via a national patient portal for a decade
- Prior research has focused on adolescents without experience of using a patient portal, while existing studies of adolescent patient portal users have been limited to contexts of serious illness.
- Adolescents report similar benefits of reading EHRs to those of adults, such as reduced anxiety, enhanced knowledge about their illness, ability to ask informed questions and reflecting more on their health

What this study adds

- Encouragement by HCPs was related to higher use frequency. However, adolescents are rarely encouraged by HCPs to read their notes online.
- Adolescents reading their records perceive all types of information in the EHR as useful.

How this study might affect research, practice or policy

- Enhanced encouragement and support for adolescents will be required to support the transition into adulthood and involve adolescents in their own care.
- EHR vendors designing patient portals and patient portals intended for adolescent use in particular may benefit from considering the types of information and functions that adolescents find useful.

Confidential: For Review Only

INTRODUCTION

An increasing number of healthcare providers are giving patients access to their electronic health records (EHR), including patient information such as clinical notes, diagnoses, and medication lists. The practice of enabling access to free-text notes written in the EHRs by healthcare professionals (HCPs) is sometimes called 'open notes'.[1] Commonly, patients gain online record access (ORA) via secure online portals. Patient-accessible electronic health records (PAEHR) are becoming increasingly expected among patients, even affecting patients' choice of healthcare provider. In particular, adolescents are growing up with expectations of digital convenience that also applies to healthcare.[2]

During early childhood, PAEHR portal systems can often allow for parental proxy access, where parents or guardians of the minor have access to the EHR of the child. However, adolescents needing healthcare face a hidden battle in balancing privacy and personal autonomy with the need for support from parents. Worries about sensitive health information (e.g. mental health issues and sexual health) being exposed to parents through the PAEHR can dissuade young people from accessing medical care.[3-4] The transition from adolescent to adult patienthood has therefore raised concerns among HCPs[5–7] and implementing online access to EHRs is especially controversial in this population.

The e-service for PAEHRs in Sweden is *Journalen*, available via the national web patient portal 1177.se, accessible to patients in all 21 Swedish regions.[8] According to publicly available statistics,[9] *Journalen* is being accessed by a steadily increasing number of users; approximately 1,360,000 unique users per month during 2022. Adult parent users have automatic access to their child's records in *Journalen*, but lose this access when the child turns 13, and adolescents gain their record access at 16. When an adolescent is between 13 and 16 years old, parents and adolescents can apply for extended access outside of the default. Adolescent patients access the same information as adults. The content includes for example a list of prescribed medications, lab results, growth curves, vaccinations, diagnoses, maternity care records, referrals, warnings, and treatment plans (Figure 1). However, availability differs according to region and connected health care providers (who have agreed to give access) (Figure 2). For example, psychiatric notes are shown in 17 of 21 regions.[10] Functions such as prescription renewal and appointment booking are conducted on the patient portal, yet externally from the PAEHR.

Figure 1. The patient-accessible electronic health record *Journalen* after log-in (manually translated), showing the functions and information available.[11] Service produced by Inera AB under the auspices of Swedish municipalities and regions.

Figure 2. The information shown in *Journalen* depending on region or health care provider (green squares) during the time of the survey.

Two literature reviews on pediatric patient portals found limited studies in this age group. One reviewed definition and utilization[12] while the other focused on use, views, and experiences among adolescents.[13] While expressing a lack of knowledge of how to use PAEHRs,[2,14] adolescents have stated a desire to receive such information from HCPs.[2,15] In a US study examining EHR access of adolescents' records by accounts divided into self, surrogate, and delegate, there was a significant increase of use during adolescence.[16] Furthermore, HCPs have stated the benefits of guiding adolescent patients to become more involved and activated in their care.[17-18] Research has mostly been observational or focused on adolescents' attitudes towards patient portals, rather than experiences. In observational studies, adolescents demonstrate the use of the same portal features as adults,[19] including lab results,[16,20-21] appointment review,[16,20-21] messaging,[16,20] and medications.[21] Existing studies of adolescent users have been limited to contexts of serious illness, and these adolescents report similar benefits of reading EHRs to those of adults, such as reduced anxiety, enhanced knowledge about their illness, ability to ask informed questions and reflecting more on their health,[22] and empowerment.[5,22] A benefit unique for adolescents concerned a supported transition into adulthood.[22] To date, no studies have systematically examined how adolescents more broadly experience ORA.

Confidential: For Review Only

Study Aim

A recent scoping review concluded that with few exceptions (Australia, Canada, New Zealand, and the United Kingdom), the entire body of literature on adolescent access to EHRs had been carried out in the United States.[13] The present research explored reasons for reading the EHRs, the perceived usefulness of information and functions in a patient portal, and the association between frequency of use and HCP encouragement to read the EHR, among adolescents in Sweden.

METHODS

Study Design

Within the NORDeHEALTH research project,[23] an open survey designed to elicit the opinions and experiences of PAEHR users was conducted. The data were collected for three weeks from January to February 2022. Convenience sampling was used. Participants were notified about the survey when they logged into their records through the Swedish national PAEHR *Journalen*, by receiving a request to voluntarily participate in the survey, along with detailed information about the study. Accessing the survey without logging in was impossible. The participation request did not appear once the patient had completed the survey. Participants provided electronic consent and there was no financial incentive. Based on the definition of adolescence proposed by the United Nations (UN), youths aged 10-19 [24], only survey respondents aged 19 and younger were included for this paper. Due to an ethical-legal requirement of written parental consent for participants younger than 15 (The Act concerning the Ethical Review of Research Involving Humans[25]), which would complicate survey distribution, we opted to exclude those younger than 15 years. Therefore, the first survey item after consent concerned age and selecting *14 or younger* triggered information about exclusion and survey termination. The Checklist for Reporting Results of Internet E-Surveys (CHERRIES)[26] was used to report this study.

Data Collection

An anonymous questionnaire covering 83 items was designed in English and translated into Swedish. The questionnaire included a mixture of close- and open-ended questions with various response options (yes/no, multiple choice, and free-text form). The design and selection of items were informed by prior studies.[1,19,27] Answering close-ended questions was mandatory, while free-text questions were optional. The Swedish survey was distributed over 12 pages and some items were conditionally displayed based on responses to other items. Including conditional questions, the number of items ranged between 1-10 item per page. Respondents could move back and forth between survey sections, however did not have an option to review the filled form before submission. For this study, five items were included (see Figure 3) based on the study's aim. Three of these items used a 5-point Likert scale (ranging from disagree to agree), and two were close-ended questions. Data on use frequency was collected to enable comparison across groups. The items used in the current study are found in Figure 3, and answer options and item number can be found in Appendix A (English original and Swedish translation). The full survey and further details about the data collection procedure are found in a separate publication.[28] It should be noted that the availability of information and functions in *Journalen* varies across regions in Sweden, which may affect respondents' assessment. However, the items on perceived usefulness are formulated anticipatory (see Figure 3).

Figure 3. Survey items included in the study.

Statistical Analysis

Only completed questionnaires were analyzed. Aside from using descriptive statistics for calculating percentages for different response options, the Fisher's exact test was used to explore the difference in use frequency based on whether a HCP (physician, nurse, psychologist, physiotherapist, or other medical staff) had encouraged PAEHR use. Non-parametric tests were conducted due to the small sample size, and the significance level was set to 95%. The use frequency response options *This is my*

Confidential: For Review Only

first time and *2 to 9 times* were merged for analysis due to a small sample. The SPSS 28 software was used for analyses, and figures were produced using R.

Patient and public involvement

The survey was tested with the public before launch to assess its clarity and response burden, leading to minor adjustments. There was no other patient and public involvement in the design or conduct of the study.

RESULTS

Characteristics of the Participants

During the study period, 23,855 users opened the survey, and 15,867 (66.5%) started filling it in. Of these, 13,010 (82.0%) submitted the survey. Two (2/13 010, 0.02%) were 14 years old or younger and were excluded from the survey. Of the 13,008 respondents who completed the survey, 218 were 15-19 years old (1.7%). Of respondents aged between 15-19, 77.1% (168/218) identified as women, 17.9% (39/218) as men while 5.0% (11/218) selected Other. See data on survey completion in a previous publication[28] and descriptive statistics for all variables in Appendix B.

Experience with Accessing Records Online

Two out of five respondents had logged in more than 20 times (85/218, 39.0%) and one third had logged in 2-9 times (74/218, 33.9%) (see Table 1).

Table 1. Participants' frequency of accessing their health records online.

**Frequency of health record access, n (%)**	**Participants (N=218)**
This is my first time	11 (5)
2 to 9 times	74 (33.9)
10-20 times	48 (22)
More than 20 times	85 (39)

Encouragement to Read the Health Records Online

One survey question focused on whether respondents had been encouraged or reminded by anyone or any media source to read their records online. The most common response was *nobody encouraged me* (118/218, 54.1%), see Figure 4. Of four respondents that chose *Other*, one respondent provided a free-text description, citing "myself". About a fifth (47/218, 21.6%) had been encouraged by some type of HCP while the remaining (171/218, 78.4%) had not, and Fisher's exact test revealed that those encouraged by a HCP were more frequent users of the patient portal than those who had not (p=.043).

Figure 4. Encouragement to read health records online. Because respondents could select multiple items, the total number can exceed 100%. Legend: *Types of HCPs.

Reasons for Reading the Health Records Online

Four reasons were chosen by more than half of respondents: *out of general curiosity* (170/218, 78.0%), *to get an overview of one's medical history and/or treatment* (159/218, 72.9%), *to ensure having understood what the physician or nurse said* (149/218, 68.3%), and *to improve one's understanding for one's health issue* (138/218, 63.3%) (see Figure 5). Of 50 respondents who selected *Other*, 16 provided free-text comments. The comments included ensuring accuracy, memory reinforcement, poor notification frequency (i.e. logging in to check for new information due to lack of notifications), specification of sought information (e.g., test results, Covid-19 vaccinations), and retrieving a record copy for monetary activity compensation.

Figure 5. Adolescents' reasons for reading health records online.

Usefulness of Portal Information and Function

All information types were considered useful by more than 3/4 of participants, see Figure 6.

Confidential: For Review Only

Figure 6. Adolescents' perceived usefulness of different types of patient portal information.

Perceived usefulness of various functions was also examined, most of which do not currently exist in *Journalen*. Most functions were perceived as useful by a majority of respondents, see Figure 7. Yet, four functions were considered useful by less than half of respondents: *accessing information and managing services for children* and for *family members*, *contributing information about expectations for the health care visit*, and *blocking specific clinical notes from certain HCPs/medical staff*. Of 24 participants who wrote a comment under *Other*, 7 referred to a specific function. Comments concerned the ability to view radiology images (median=4.5), diagnosis (median=3.5), dentistry records (median=5), ability to read children's records when under 18 (median=5), records from childhood (median=5), and to view appointments from gynecologic care (median=5). Other free-text answers were non-responses or unspecified. *Medical certificates and other certificates* concerned certificates from HCPs, such as COVID vaccination and for insurance and sick leave.

Figure 7. Adolescents' perceived usefulness of patient portal functions.

DISCUSSION

In our national survey of PAEHR experiences in Sweden, adolescents perceived all proposed clinical information as consistently useful. Ratings for portal functions varied. Common reasons for reading records were curiosity and to get an overview of medical history. Sharing clinical information was the least common reason for accessing the record. Encouragement by HCPs to read records was associated with higher use. The reasons for reading records align with previous research on adults[19] and US adolescents.[22]

While all information types were rated highly on usefulness, test results were considered the most useful. This result accords with earlier research on adolescents,[16,20-21] but also with research on parents[29-30] and general adult populations.[19] Unsurprisingly, adolescents rated the ability to manage family members or children low. The function perceived as the third most useful was the ability to order and manage various certificates. This was likely associated with the timing of survey distribution, as the ongoing COVID-19 pandemic required Swedish citizens to present COVID-19 certificates for traveling and attending events. The boost of PAEHR use as a result of the COVID-19 pandemic has been mentioned previously.[31] Related to this, it is somewhat unexpected that immunizations were rated among the least useful types of information. However, Swedish regions differed in managing COVID-19 vaccination through the national e-service or other apps, and not all regions displayed information on vaccinations. Another finding was that adolescents do not read their PAEHRs to share information. This finding is consistent with a previous survey study based in Canada, where adolescents without access to their records anticipated not being likely to share information from the records with others.[32]

Encouragement from HCPs was linked to higher use, indicating the influence of social factors in eHealth adoption.[33] Another possible explanation is that those with more active medical issues needed to review their health record more frequently. Encouragement from HCPs and family/friends has increased PAEHR use in adults,[34] suggesting that this may be an important factor influencing adoption and use also in adolescents. Lack of HCP encouragement aligns with research on adults in the United States[35] and Sweden,[36] and may stem from concerns about medical literacy, increased workload, and uncertainty about visible information.[2,5,37–39] Not encouraging adolescents may lead to a missed opportunity for early patient involvement.[17] Increased PAEHR usage among adolescents can motivate policy-making stakeholders and EHR vendors to improve patient portals for this population.[5]

This study has limitations. Response biases in self-report surveys are unknown. Survey availability on the national patient portal may have influenced respondents' decisions to respond based on their competence and skills in using the service, potentially limiting the generalizability of the findings and causing positive bias as most adolescent respondents were frequent users. However, we opted to use convenience sampling as part of an exploratory approach. The majority of participants were women, which is consistent with adult and previous adolescent PAEHR users but represents a

Confidential: For Review Only

strong gender imbalance. Only 1.7% of the total respondents were aged 15-19, possibly due to younger individuals being less affected by health conditions and less engaged in personal health management. The survey was also designed for adults, and adolescents may have struggled to understand some questions. Furthermore, the reliability and validity of the survey utilized in this study have not been formally evaluated. While the survey was designed to capture the intended data, a comprehensive assessment of its psychometric properties was beyond the scope of this research. Still, the survey was tested with the public, as stated under Patient and public involvement.

The present results provide a broader understanding of adolescents reading their EHRs, however further work is required to establish the viability of the findings in other settings. Several questions remain, relating to for example eHealth literacy and confidentiality. It should be noted that the current study included only a subset of items from a larger survey, and there is an intention to publish additional findings on the adolescent population, on participants' views about security and privacy. Regarding our research methodology, we offer a few suggestions for future work. First, frequency of use was defined by how many times (first time, 2-9 times etc.) the users accessed their records in the past 12 months. Another approach is to ask how often users access their records (daily, weekly, etc.), which allows for an understanding of the visit regularity. Second, further research may benefit from providing a clearer distinction between a specific PAEHR service and patients' ORA more broadly. Lastly, including a larger and more evenly gender-distributed sample and specific patient groups with chronic diseases could furnish greater insights into adolescent users' experiences.

Data availability statement

Data are available from the corresponding author on reasonable request.

Ethics statements

Patient consent for publication

Electronic consent obtained from survey respondents.

Ethics approval

Ethical approval for this study was obtained from the Regional Ethical Review Board in Uppsala, Sweden (EPN 2021/05229). All respondents received study information and provided informed consent.

Abbreviations

EHR: electronic health record
HCP: healthcare professional
ORA: online record access
PAEHR: patient-accessible electronic health record

FOOTNOTES

Contributors: JH wrote the manuscript, created the tables, and designed the figures. MH, CB, IS, JM, ÅC, and HR contributed to the study design. All authors read the manuscript, provided feedback, and approved the final draft for submission.

Funding: This work was supported by NordForsk (grant number #100477) and the Swedish Research Council for Health, Working Life and Welfare (FORTE) (grant number #2020-01229).

Competing interests: None.

Patient and public involvement: Patients and/or the public were involved in survey testing but otherwise not involved in the design, or conduct, or reporting, or dissemination plans of this research.

Provenance and peer review: Not commissioned; externally peer reviewed.

Confidential: For Review Only

Figure captions:
Figure 1. The patient-accessible electronic health record *Journalen* after log-in (manually translated), showing the functions and information available.[11] Service produced by Inera AB under the auspices of Swedish municipalities and regions.
Figure 2. The information shown in *Journalen* depending on region or health care provider (green squares) during the time of the survey.
Figure 3. Survey items included in the study.
Figure 4. Encouragement to read health records online. Because respondents could select multiple items, the total number can exceed 100%. Legend: *Types of HCPs.
Figure 5. Adolescents' reasons for reading health records online.
Figure 6. Adolescents' perceived usefulness of different types of patient portal information.
Figure 7. Adolescents' perceived usefulness of patient portal functions.

REFERENCES

1. Walker J, Leveille S, Bell S, et al. OpenNotes After 7 Years: Patient Experiences With Ongoing Access to Their Clinicians' Outpatient Visit Notes. J Med Internet Res. 2019;21(5):e13876. doi:10.2196/13876
2. Beaton L, Williams I, Sanci L. Exploring adolescent and clinician perspectives on Australia. Aust J Prim Health. Published online March 15, 2021. doi:10.1071/PY20169
3. Bayer R, Santelli J, Klitzman R. New Challenges for Electronic Health Records: Confidentiality and Access to Sensitive Health Information About Parents and Adolescents. JAMA. 2015;313(1):29. doi:10.1001/jama.2014.15391
4. Ford CA. Foregone Health Care Among Adolescents. JAMA. 1999;282(23):2227. doi:10.1001/jama.282.23.2227
5. Carlson JL, Goldstein R, Buhr T, Buhr N. Teenager, parent, and clinician perspectives on the electronic health record. Pediatrics. 2020;145(3). doi:10.1542/peds.2019-0193
6. Goldstein RL, Anoshiravani A, Svetaz MV, Carlson JL. Providers' Perspectives on Adolescent Confidentiality and the Electronic Health Record: A State of Transition. J Adolesc Health. 2020;66:296-300. doi:10.1016/j.jadohealth.2019.09.020
7. Stablein T, Loud KJ, DiCapua C, Anthony DL. The Catch to Confidentiality: The Use of Electronic Health Records in Adolescent Health Care. J Adolesc Health. 2018;62(5):577-582. doi:10.1016/j.jadohealth.2017.11.296
8. Hägglund M, Scandurra I. Patients' Online Access to Electronic Health Records: Current Status and Experiences from the Implementation in Sweden. MEDINFO 2017 Precis Healthc Inform. Published online 2017:723-727. doi:10.3233/978-1-61499-830-3-723
9. Statistik för Ineras tjänster [Statistics for Inera's services]. Inera AB. Accessed August 25, 2023. https://www.inera.se/tjanster/statistik-for-ineras-tjanster/
10. Bärkås A, Scandurra I, Rexhepi H, Blease C, Cajander Å, Hägglund M. Patients' Access to Their Psychiatric Notes: Current Policies and Practices in Sweden. Int J Environ Res Public Health. 2021;18(17):9140. doi:10.3390/ijerph18179140
11. Journalen. Inera. Published December 2022. Accessed December 8, 2022. https://journalen.1177.se/
12. Bush RA, Connelly CD, Fuller M, Pérez A. Implementation of the Integrated Electronic Patient Portal in the Pediatric Population: A Systematic Review. Telemed J E-Health Off J Am Telemed Assoc. 2016;22(2):144-152. doi:10.1089/tmj.2015.0033
13. Hagström J, Blease C, Haage B, Scandurra I, Hansson S, Hägglund M. Use, Views of and Experiences of Web-Based Access to Electronic Health Records for Children, Adolescents, and Parents: Scoping Review. J Med Internet Res. Published online 2022. doi:10.2196/40328
14. Miklin DJ, Vangara SS, Delamater AM, Goodman KW. Understanding of and Barriers to Electronic Health Record Patient Portal Access in a Culturally Diverse Pediatric Population. JMIR Med Inform. 2019;7(2):e11570. doi:10.2196/11570

Confidential: For Review Only

15. Bergman DA, Brown NL, Wilson S. Teen Use of a Patient Portal: A Qualitative Study of Parent and Teen Attitudes. Perspect Health Inf Manag. 2008;5:13.
16. Steitz BD, Cronin RM, Davis SE, Yan E, Jackson GP. Long-term patterns of patient portal use for pediatric patients at an academic medical center. Appl Clin Inform. 2017;8(3):779-793. doi:10.4338/ACI-2017-01-RA-0005
17. Bourgeois FC, DesRoches CM, Bell SK. Ethical Challenges Raised by OpenNotes for Pediatric and Adolescent Patients. Pediatrics. 2018;141(6):e20172745. doi:10.1542/peds.2017-2745
18. Lam BD, Bourgeois F, DesRoches CM, Dong Z, Bell SK. Attitudes, experiences, and safety behaviours of adolescents and young adults who read visit notes: Opportunities to engage patients early in their care. Future Heal J. 2021;8(3):e585-e592. doi:10.7861/fhj.2021-0118
19. Moll J, Rexhepi H, Cajander Å, et al. Patients' Experiences of Accessing Their Electronic Health Records: National Patient Survey in Sweden. J Med Internet Res. 2018;20(11):e9492. doi:10.2196/jmir.9492
20. Calman N, Pfister HR, Lesnewski R, Hauser D, Shroff N. Electronic Access to Adolescents' Health Records: Legal, Policy, and Practice Implications. Vol 22.; 2014:11-14. https://www.aafp.org/fpm/2015/0300/p11.html
21. Thompson LA, Martinko T, Budd P, Mercado R, Schentrup AM. Meaningful use of a confidential adolescent patient portal. J Adolesc Health. 2016;58(2):134-140. doi:10.1016/j.jadohealth.2015.10.015
22. Hong MK, Wilcox L, Feustel C, Wasileski-Masker K, Olson TA, Simoneaux SF. Adolescent and Caregiver use of a Tethered Personal Health Record System. AMIA Annu Symp Proc AMIA Symp. 2016;2016:628-637.
23. Hägglund M. Nordic countries lead new initiative on patient access to EHRs. TheBMJopinion. Published May 18, 2021. Accessed January 16, 2022. https://blogs.bmj.com/bmj/2021/05/18/maria-hagglund-nordic-countries-lead-new-initiative-on-patient-access-to-ehrs/
24. "Definition of Youth." United Nations Department of Economic and Social Affairs (UNDESA).
25. Riksdagsförvaltningen. 2003:460.; 2003. Accessed August 3, 2022. https://www.riksdagen.se/sv/dokument-lagar/dokument/svensk-forfattningssamling/lag-2003460-om-etikprovning-av-forskning-som_sfs-2003-460
26. Eysenbach G. Improving the Quality of Web Surveys: The Checklist for Reporting Results of Internet E-Surveys (CHERRIES). J Med Internet Res. 2004;6(3):e34. doi:10.2196/jmir.6.3.e34
27. Zanaboni P, Kummervold PE, Sørensen T, Johansen MA. Patient use and experience with online access to electronic health records in Norway: Results from an online survey. J Med Internet Res. 2020;22(2). doi:10.2196/16144
28. Hägglund M, Kharko A, Hagström J, et al. The NORDeHEALTH 2022 Patient Survey: A cross-sectional survey of national patient portal users in Norway, Sweden, Finland, and Estonia. J Med Internet Res. 2023;25:e47573. doi:10.2196/47573
29. Ahlers-Schmidt CR, Nguyen M. Parent Intention to Use a Patient Portal as Related to Their Children Following a Facilitated Demonstration. Telemed E-Health. 2013;19(12):979-981. doi:10.1089/tmj.2013.0041
30. Thompson LA, Black EW, Saliba H, Schentrup AM. Parents' knowledge of and opinions about healthcare laws and technology in primary care. Inform Prim Care. 2012;20(1):69-74. doi:10.14236/jhi.v20i1.49
31. Blease C, Salmi L, Hägglund M, Wachenheim D, DesRoches C. COVID-19 and Open Notes: A New Method to Enhance Patient Safety and Trust. JMIR Ment Health. 2021;8(6):e29314. doi:10.2196/29314
32. Frazer C, Ratchford F, Roch J. Enabling Adolescent Electronic Access to Personal Health Information. Stud Health Technol Inform. 2017;234:115-119.
33. Venkatesh V, Morris MG, Davis GB, Davis FD. User Acceptance of Information Technology: Toward a Unified View. MIS Q. 2003;27(3):425-478. doi:10.2307/30036540

Confidential: For Review Only

34. Mukhopadhyay S, Basak R, Carpenter D, Reithel BJ. Patient use of online medical records: an application of technology acceptance framework. Inf Comput Secur. 2019;28(1):97-115. doi:10.1108/ICS-07-2019-0076
35. DesRoches CM, Leveille S, Bell SK, et al. The Views and Experiences of Clinicians Sharing Medical Record Notes With Patients. JAMA Netw Open. 2020;3(3):e201753. doi:10.1001/jamanetworkopen.2020.1753
36. Rexhepi H, Moll J, Huvila I. Online electronic healthcare records: Comparing the views of cancer patients and others. Health Informatics J. 2020;26(4):2915-2929. doi:10.1177/1460458220944727
37. Hagström J, Blease C, Haage B, Scandurra I, Hansson S, Hägglund M. Views, Use, and Experiences of Web-Based Access to Pediatric Electronic Health Records for Children, Adolescents, and Parents: Scoping Review. J Med Internet Res. 2022;24(11):e40328. doi:10.2196/40328
38. Grünloh C, Myreteg G, Cajander Å, Rexhepi H. "Why Do They Need to Check Me?" Patient Participation Through eHealth and the Doctor-Patient Relationship: Qualitative Study. J Med Internet Res. 2018;20(1):e8444. doi:10.2196/JMIR.8444
39. Petersson J, Backman C. Patient-accessible online health records: Reconfigurations of clinical rhythms and doctors' front- and backstage spaces. Soc Sci Med. 2022;292:114635. doi:10.1016/j.socscimed.2021.114635

Confidential: For Review Only

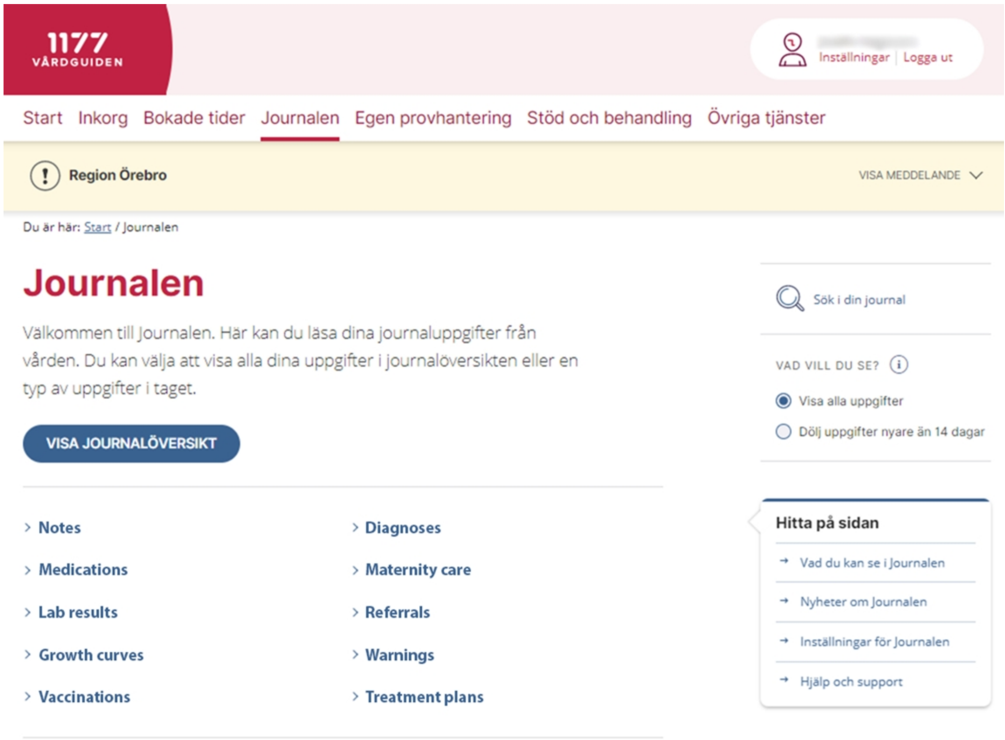

Figure 1. The patient-accessible electronic health record Journalen after log-in (manually translated), showing the functions and information available.[11] Service produced by Inera AB under the auspices of Swedish county councils and regions.

578x427mm (300 x 300 DPI)

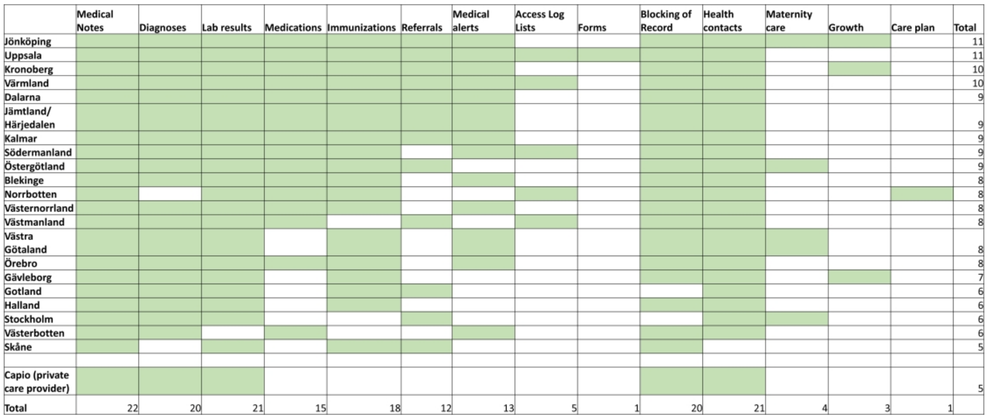

	Medical Notes	Diagnoses	Lab results	Medications	Immunizations	Referrals	Medical alerts	Access Log Lists	Forms	Blocking of Record	Health contacts	Maternity care	Growth	Care plan	Total
Jönköping															11
Uppsala															11
Kronoberg															10
Värmland															10
Dalarna															9
Jämtland/ Härjedalen															9
Kalmar															9
Södermanland															9
Östergötland															9
Blekinge															8
Norrbotten															8
Västernorrland															8
Västmanland															8
Västra Götaland															8
Örebro															8
Gävleborg															7
Gotland															6
Halland															6
Stockholm															6
Västerbotten															6
Skåne															5
Capio (private care provider)															5
Total	22	20	21	15	18	12	13	5	1	20	21	4	3	1	

Figure 2. The information shown in Journalen depending on region or health care provider (green squares) during the time of the survey.

758x320mm (300 x 300 DPI)

1. How often do you think that you have accessed your health record during the last 12 months?
2. Did any of the following encourage or remind you to read your health record? (check all that apply)
3. Please indicate how much you disagree or agree with the following statement: I read my health record online… [reasons for reading]
4. How useful would it be to have access to the following information in the portal?
5. How useful would it be to have access to the following functions in the portal?

Figure 3. Survey questions included in the study.

520x176mm (300 x 300 DPI)

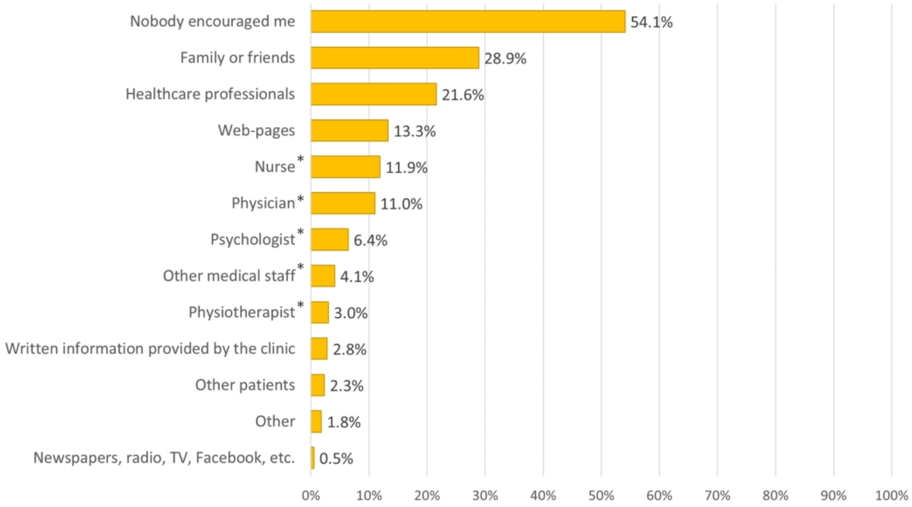

Figure 4. Encouragement to read health records online. Because respondents could select yes for multiple items, the total number can exceed 100%. Legend: *Types of HCPs.

478x250mm (300 x 300 DPI)

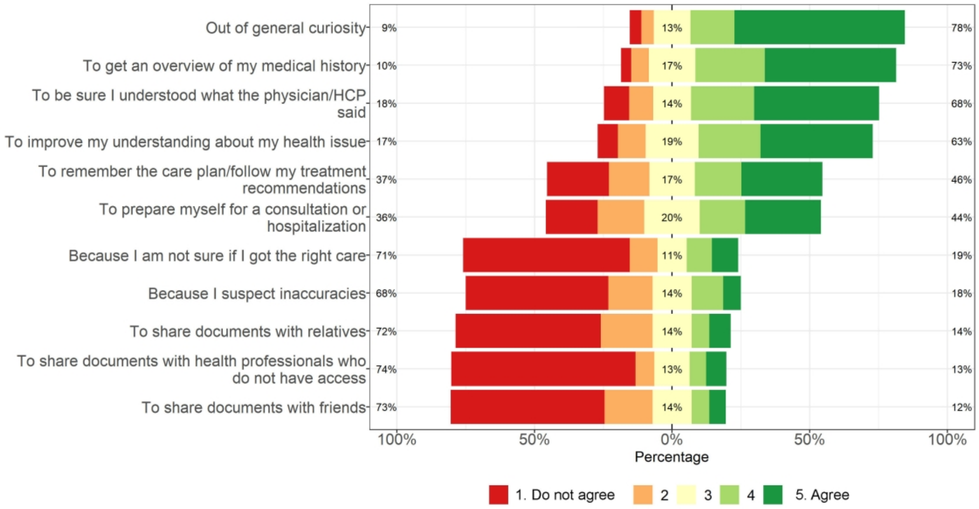

Figure 5. Adolescents' reasons for reading health records online.

292x152mm (300 x 300 DPI)

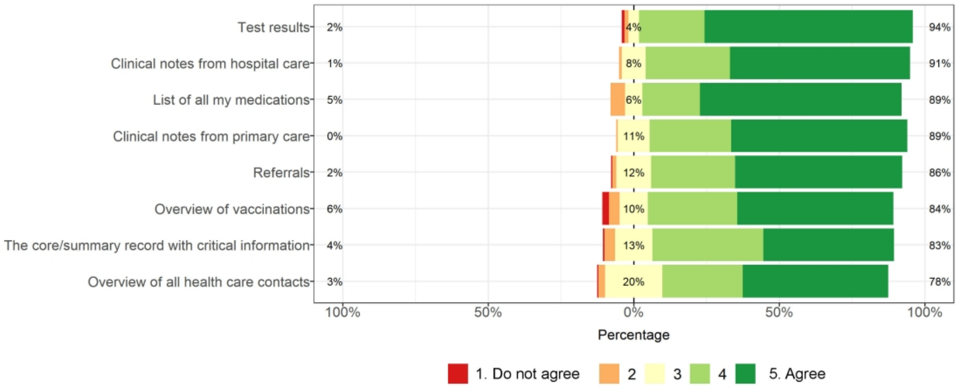

Figure 6. Adolescents' perceived usefulness of different types of patient portal information.

279x112mm (300 x 300 DPI)

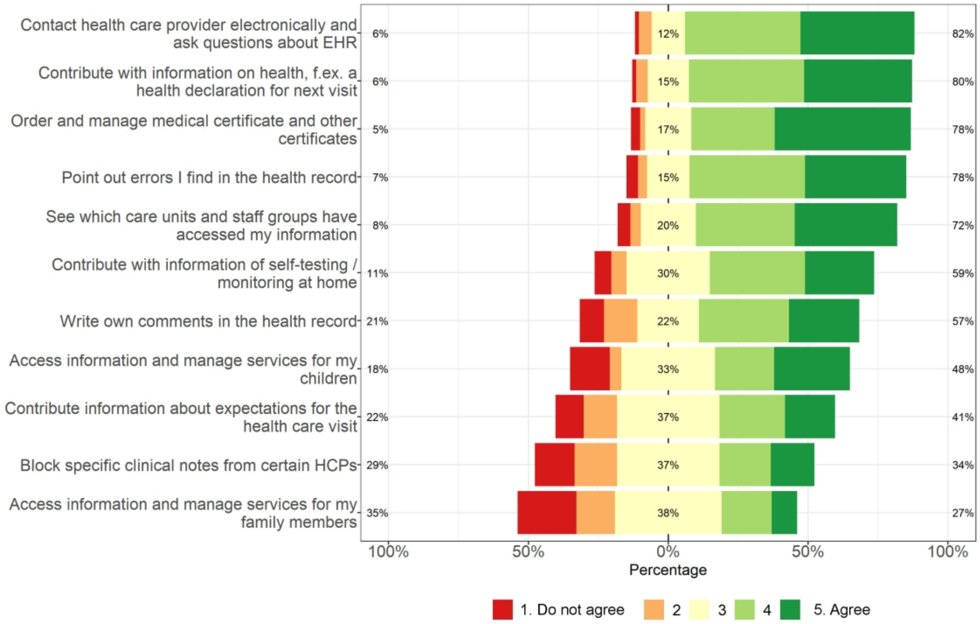

Figure 7. Adolescents' perceived usefulness of patient portal functions. Legend: *Functions that exist in all or some regions in Sweden. Only adult users over 18 years old have access to their children's records.

279x177mm (300 x 300 DPI)

Appendix A

Table 1. Original survey created in English

Number in survey	Question	Response alternatives
6	**How often do you think that you have accessed your health record during the last 12 months?**	1 - This is my first time<br>2 - 2 to 9 times<br>3 - 10 to 20 times<br>4 - More than 20 times
7	**Did any of the following encourage or remind you to read your health record?**	HCPs: Physician<br>HCPs: Nurse<br>HCPs: Psychologist<br>HCPs: Physiotherapist<br>HCPs: Other medical staff<br>Written information provided by the hospital/healthcare facility /clinic<br>Family or friends<br>Web-pages, such as the National health portal web-page, etc.<br>Newspapers, radio, TV, Facebook, etc.<br>Other patients<br>Nobody encouraged me<br>Other
8	Please indicate how much you disagree or agree with the following statement:<br>**I read my health record online…**	1 - Disagree,<br>2, 3, 4,<br>5 - Agree
		out of general curiosity
		to improve my understanding about my health issue
		to prepare myself for a consultation or hospitalization
		to get an overview of my medical history and/or treatment
		to be sure I understood what the physician/healthcare professional said
		to remember the care plan/follow my treatment recommendations
		because I suspect inaccuracies
		to share documents with relatives
		to share documents with friends
		to share documents with health professionals who do not have access
		because I am not sure if I got the right care
		Other, please specify

38	**How useful would it be to have access to the following information in the portal?**		1 - Not useful at all<br>2 - Not very useful<br>3 - Neither useful nor useless<br>4 - Useful<br>5 - Very useful
		The core/summary record with critical information	
		Referrals (content and how it is handled in care)	
		List of all pharmaceuticals/my medications	
		Overview of vaccinations	
		Results of tests	
		Clinical notes from primary care	
		Clinical notes from hospital care	
		Overview of all health care contacts	
39	**How useful would it be to have access to the following functions in the portal?**		1 - Not useful at all<br>2 - Not very useful<br>3 - Neither useful nor useless<br>4 - Useful<br>5 - Very useful
		Ability to access information and manage services for my children	
		Ability to access information and manage services for my family members	
		See which care units and staff groups have accessed my information (see log data)	
		Ability to block certain medical records from access by other medical staff	
		Ability to contact health care provider electronically and ask questions about medical record	
		Ability to point out errors I find in the health record	
		Ability to write own comments to text in the health record	
		Contribute with information on health, for example by providing a health declaration for next visit	
		Contribute with information of self-testing/monitoring at home	
		Contribute information about expectations for the health care visit	
		Ability to order and manage medical certificate and other certificates	
		Other	

Table B2. Swedish translation of survey

Number in survey	Question	Response alternatives
6	**Hur ofta har du läst i din journal under de senaste 12 månaderna?**	1 - Det här är första gången<br>2 - 2-9 gånger<br>3 - 10-20 gånger<br>4 - Mer än 20 gånger
7	**Har du blivit uppmuntrad eller påmind att läsa din journal av någon/något av följande:**	Vårdpersonal: Läkare<br>Vårdpersonal: Sjuksköterska<br>Vårdpersonal: Psykolog<br>Vårdpersonal: Fysioterapeut/sjukgymnast<br>Vårdpersonal: Annan vårdpersonal<br>Skriftlig information från sjukhuset/vårdinrättning/klinik<br>Familj eller vänner<br>Webbsidor, såsom 1177.se Vårdguiden, etc.<br>Tidningar, radio, TV, Facebook, etc.<br>Andra patienter<br>Ingen har uppmuntrat eller påmint mig<br>Annat
8	Markera i vilken utsträckning du instämmer med följande påståenden:<br>**Jag läser min journal på internet…**	1 - Instämmer inte, 2, 3, 4,<br>5 - Instämmer
		Av nyfikenhet
		För att förbättra min förståelse för mitt hälsotillstånd
		För att förbereda mig själv för ett vårdbesök eller sjukhusvistelse
		För att få en överblick av min hälsohistoria och/eller bakgrund
		För att försäkra mig om att jag förstått vad läkaren/vårdpersonalen sagt
		För att komma ihåg vårdplanen/följa mina behandlingsrekommendationer
		För att jag misstänker felaktigheter
		För att dela med mig av information till släkt
		För att dela med mig av information till vänner
		För att dela med mig av information till vårdpersonal som inte har tillgång till min journal
		För att jag är osäker på om jag fått rätt vård
		Annat, specificera gärna

38	**Hur användbart skulle det vara för dig att ha tillgång till följande information i *Journalen*?**	1 - Inte alls användbart<br>2 - Inte särskilt användbart<br>3 - Varken eller<br>4 - Användbart<br>5 - Väldigt användbart
		En sammanfattning av journalen med viktig information
		Remisser (innehåll och status)
		Lista över mina läkemedel/mediciner
		Vaccinationer
		Provsvar
		Journalanteckningar från primärvården
		Journalanteckningar från specialistvården
		Överblick av samtliga kontakter med vården
39	**Hur användbart skulle det vara för dig att ha tillgång till följande funktioner i *Journalen*?**	1 - Inte alls användbart<br>2 - Inte särskilt användbart<br>3 - Varken eller<br>4 - Användbart<br>5 - Väldigt användbart
		Möjlighet att ta del av information och hantera tjänster för mina barn
		Möjlighet att ta del av information och hantera tjänster för andra familjemedlemmar
		Möjlighet att kunna blockera specifika journalanteckningar för viss vårdpersonal
		Se vilka vårdinrättningar och personal som har läst i min journal (loggar)
		Möjlighet att kunna kontakta vårdpersonal online för att ställa frågor om min journal
		Möjlighet att påpeka felaktigheter i journalen
		Möjlighet att skriva egna kommentarer i journalen
		Möjlighet att bidra med information om min hälsa, t ex genom att fylla i en hälsodeklaration, inför ett besök
		Möjlighet att bidra med information från självtest/monitorering hemma
		Möjlighet att bidra med information om mina förväntningar på vårdbesöket
		Möjlighet att beställa och hantera sjukskrivning, medicinsk intyg och andra dokument (vaccinationsintyg t ex)
		Annat

Appendix B

Detailed results from the survey questions chosen for this study.

Encouragement to Read.

**Did any of the following encourage or remind you to read your health record? n (%)**	**Frequency**
HCPs: Physician	24 (11.0)
HCPs: Nurse	26 (11.9)
HCPs: Psychologist	14 (6.4)
HCPs: Physiotherapist	6 (2.8)
HCPs: Other medical staff	9 (4.1)
Written information provided by the hospital/healthcare facility/clinic	12 (5.5)
Family or friends	63 (28.9)
Web-pages, such as the National health portal web-page, etc.	29 (13.3)
Newspapers, radio, TV, Facebook, etc.	1 (0.5)
Other patients	5 (2.3)
Nobody encouraged me	118 (54.1)
Other	4 (1.8)
**Encouraged by HCP or not, n (%)**	
Encouraged by HCP	47 (21.6)
Not encouraged by HCP	171 (78.4)

Reasons for reading health records online.

**I read my health record online…, n** (%)	**1. Disagree**	**2**	**3**	**4**	**5. Agree**
… out of general curiosity	9 (4.1)	10 (4.6)	29 (13.3)	35 (16.1)	135 (61.9)
… to improve my understanding about my health issue	16 (7.3)	22 (10.1)	42 (19.3)	49 (22.5)	89 (40.8)
… to prepare myself for a consultation or hospitalization	41 (18.8)	37 (17.0)	44 (20.2)	36 (16.5)	60 (27.5)
… to get an overview of my medical history and/or treatment	8 (3.7)	14 (6.4)	37 (17.0)	55 (25.2)	104 (47.7)
… to be sure I understood what the physician/healthcare professional said	20 (9.2)	19 (8.7)	30 (13.8)	50 (22.9)	99 (45.4)
… to remember the care plan/follow my treatment recommendations	49 (22.5)	32 (14.7)	36 (16.5)	37 (17.0)	64 (29.4)
… because I suspect inaccuracies	113 (51.8)	35 (16.1)	31 (14.2)	25 (11.5)	14 (6.4)
… to share documents with relatives	115 (52.8)	41 (18.8)	31 (14.2)	14 (6.4)	17 (7.8)
… to share documents with friends	122 (56.0)	38 (17.4)	31 (14.2)	14 (6.4)	13 (6.0)
… to share documents with healthcare professionals who do not have access	146 (67.0)	15 (6.9)	28 (12.8)	13 (6.0)	16 (7.3)
… because I am not sure if I got the right care	132 (60.6)	22 (10.1)	23 (10.6)	20 (9.2)	21 (9.6)
Other	168 (77.1)	20 (9.2)	5 (2.3)	3 (1.4)	22 (10.1)

Perceived usefulness of various types of information.

**Information**	**Not useful at all**	**Not very useful**	**Neither useful nor useless**	**Useful**	**Very useful**
The core/summary record with critical information	1 (0.5)	8 (3.7)	28 (12.8)	83 (38.1)	98 (45.0)
Referrals (content and how it is handled in care)	1 (0.5)	3 (1.4)	26 (11.9)	63 (28.9)	125 (11.9)
List of all pharmaceuticals / my medications	0 (0)	11 (5.0)	13 (6.0)	43 (19.7)	151 (69.3)
Overview of vaccinations	5 (2.3)	8 (3.7)	21 (9.6)	67 (30.7)	117 (53.7)
Results of tests (not available in all regions)	2 (0.9)	3 (1.4)	8 (3.7)	49 (22.5)	156 (71.6)
Clinical notes from primary care	0 (0)	1 (0.5)	24 (11.0)	61 (28.0)	132 (60.6)
Clinical notes from hospital care	0 (0)	2 (0.9)	18 (8.3)	63 (28.9)	135 (61.9)
Overview of all health care contacts	1 (0.5)	5 (2.3)	43 (19.7)	60 (27.5)	109 (50)

Perceived usefulness of various functions.

Function	Not useful at all	Not very useful	Neither useful nor useless	Useful	Very useful
Ability to access information and manage services for my children (not available in all regions).	31 (14.2)	9 (4.1)	73 (33.5)	46 (21.1)	59 (27.1)
Ability to access information and manage services for my family members.	46 (21.1)	30 (13.8)	83 (38.1)	39 (17.9)	20 (9.2)
Ability to block specific clinical notes from certain healthcare professionals/medical staff.	31 (14.2)	33 (15.1)	80 (36.7)	40 (18.3)	34 (15.6)
See which care units and staff groups have accessed my information (see log data).	10 (4.6)	8 (3.7)	43 (19.7)	77 (35.3)	80 (36.7)
Ability to contact health care provider electronically and ask questions about medical record.	3 (1.4)	10 (4.6)	26 (11.9)	90 (41.3)	89 (40.8)
Ability to point out errors I find in the health record.	9 (4.1)	7 (3.2)	33 (15.1)	90 (41.3)	79 (36.2)
Ability to write own comments to text in the health record.	19 (8.7)	26 (11.9)	48 (22.0)	70 (32.1)	55 (25.2)
Contribute with information on health, for example by providing a health declaration for next visit.	3 (1.4)	9 (4.1)	32 (14.7)	90 (41.3)	84 (38.5)
Contribute with information of self-testing/monitoring at home.	13 (6.0)	12 (5.5)	65 (29.8)	74 (33.9)	54 (24.8)
Contribute information about expectations for the health care visit.	22 (10.1)	26 (11.9)	80 (36.7)	51 (23.4)	39 (17.9)
Ability to order and manage medical certificate and other certificates.	7 (3.2)	4 (1.8)	36 (16.5)	65 (29.8)	106 (48.6)

BMJ Paediatrics Open

Adolescents’ reasons for accessing their health records online, perceived usefulness, and experienced provider encouragement: A national survey in Sweden

Journal:	*BMJ Paediatrics Open*
Manuscript ID	bmjpo-2023-002258.R3
Article Type:	Original research
Date Submitted by the Author:	07-Feb-2024
Complete List of Authors:	Hagström, Josefin; Uppsala Universitet, Department of Women's and Children's Health; Uppsala University Hospital, Medtech Science & Innovation Centre<br>Blease, Charlotte; Uppsala Universitet, Department of Women's and Children's Health; Harvard Medical School, Beth Israel Deaconess Medical Center<br>Scandurra, Isabella; Örebro University, School of Business<br>Moll, Jonas; Örebro University, School of Business<br>Cajander, Åsa; Uppsala Universitet, Department of Information Technology<br>Rexhepi, Hanife; University of Skövde, School of Informatics<br>Hägglund, Maria; Uppsala Universitet, Department of Women's and Children's Health; Uppsala University Hospital, Medtech Science & Innovation Centre
Keywords:	Adolescent Health, Health services research, Data Collection

SCHOLARONE™
Manuscripts

I, the Submitting Author has the right to grant and does grant on behalf of all authors of the Work (as defined in the below author licence), an exclusive licence and/or a non-exclusive licence for contributions from authors who are: i) UK Crown employees; ii) where BMJ has agreed a CC-BY licence shall apply, and/or iii) in accordance with the terms applicable for US Federal Government officers or employees acting as part of their official duties; on a worldwide, perpetual, irrevocable, royalty-free basis to BMJ Publishing Group Ltd ("BMJ") its licensees and where the relevant Journal is co-owned by BMJ to the co-owners of the Journal, to publish the Work in this journal and any other BMJ products and to exploit all rights, as set out in our licence.

The Submitting Author accepts and understands that any supply made under these terms is made by BMJ to the Submitting Author unless you are acting as an employee on behalf of your employer or a postgraduate student of an affiliated institution which is paying any applicable article publishing charge ("APC") for Open Access articles. Where the Submitting Author wishes to make the Work available on an Open Access basis (and intends to pay the relevant APC), the terms of reuse of such Open Access shall be governed by a Creative Commons licence – details of these licences and which Creative Commons licence will apply to this Work are set out in our licence referred to above.

Other than as permitted in any relevant BMJ Author's Self Archiving Policies, I confirm this Work has not been accepted for publication elsewhere, is not being considered for publication elsewhere and does not duplicate material already published. I confirm all authors consent to publication of this Work and authorise the granting of this licence.

Confidential: For Review Only

TITLE PAGE

Article title: Adolescents' reasons for accessing their health records online, perceived usefulness, and experienced provider encouragement: A national survey in Sweden

Author information:
Corresponding author:
Josefin Hagström, MSc[1,2] (ORCID: 0000-0003-2835-0259)
Department of Women's and Children's Health, Uppsala University, Uppsala, Sweden

josefin.hagstrom@uu.se
+46184715080

Co-authors:
Charlotte Blease PhD[1,3] (ORCID: 0000-0002-0205-1165)
Isabella Scandurra PhD[4] (ORCID: 0000-0002-2597-1079)
Jonas Moll PhD[4] (ORCID: 0000-0002-4772-4730)
Åsa Cajander PhD[5] (ORCID: 0000-0001-7472-2215)
Hanife Rexhepi PhD[6] (ORCID: 0000-0001-8957-9853)
Maria Hägglund PhD[1,2] (ORCID: 0000-0002-6839-3651)

[1] Department of Women's and Children's Health, Uppsala University, Uppsala, Sweden
[2] Medtech Science & Innovation Centre, Uppsala University Hospital, Uppsala, Sweden
[3] Beth Israel Deaconess Medical Center, Harvard Medical School, Boston, MA, USA
[4] School of Business, Örebro University, Örebro, Sweden
[5] Department of Information Technology, Uppsala University, Uppsala, Sweden
[6] School of Informatics, University of Skövde, Skövde, Sweden

Disclaimers: The views expressed in the submitted article are his or her own and not an official position of the institution or funder.

Source(s) of support: This work was supported by NordForsk (grant number #100477) and the Swedish Research Council for Health, Working Life and Welfare (FORTE) (grant number #2020-01229).

Word count: 2511

Number of figures: 7

Number of tables: 2

Key words: adolescents; adolescent health; Patient Accessible Electronic Health Record (PAEHR); Electronic Health Record (EHR); patient portal; national survey; ehealth

References: 39

Disclosure of relationships and activities: There are no conflicts of interest to declare.

Confidential: For Review Only

ABSTRACT

Background: Having online access to electronic health records (EHRs) may help patients become engaged in their care at an early age. However, little is known about adolescents using patient portals. A national survey conducted within the Nordic eHealth project NORDeHEALTH provided an important opportunity to advance our understanding of adolescent users of patient portals. The present study explored reasons for reading the EHRs, the perceived usefulness of information and functions in a patient portal, and the association between frequency of use and encouragement to read the EHR.
Methods: Data were collected in a survey using convenience sampling, available through the Swedish online health portal during three weeks in January and February 2022. This study included a subset of items and only respondents aged 15-19. Demographic factors and frequencies on Likert-style questions were reported with descriptive statistics, while Fisher's exact test was used to explore differences in use frequency based on having been encouraged to read by a HCP.
Results: Of 13 008 users who completed the survey, 218 (1.7%) were unique users aged 15-19 (females: 77.1%). One fifth (47/218, 21.6%) had been encouraged by HCPs to read their records, and having been encouraged by HCP was related to higher use frequency (p=.043). All types of information were rated high on usefulness, while some functions were rated low, such as blocking specific clinical notes from HCPs and managing services for family members. The main reason for reading their health records online was out of curiosity.
Conclusions: Adolescents who read their records online perceive it to be useful. Encouragement by HCPs can lead to increased use of patient portals among adolescents. Findings should be considered in the future design of patient portals for adolescents.

Keywords: adolescents; adolescent health; Patient Accessible Electronic Health Record (PAEHR); Electronic Health Record (EHR); patient portal; national survey; ehealth

KEY MESSAGES

What is already known on this topic

- Implementation of online record access for adolescents is controversial
- Citizens in Sweden have had online record access via a national patient portal for a decade
- Prior research has focused on adolescents without experience of using a patient portal, while existing studies of adolescent patient portal users have been limited to contexts of serious illness.
- Adolescents report similar benefits of reading EHRs to those of adults, such as reduced anxiety, enhanced knowledge about their illness, ability to ask informed questions and reflecting more on their health

What this study adds

- Encouragement by HCPs was related to higher use frequency. However, adolescents are rarely encouraged by HCPs to read their notes online.
- Adolescents reading their records perceive all types of information in the EHR as useful.

How this study might affect research, practice or policy

- Enhanced encouragement and support for adolescents will be required to support the transition into adulthood and involve adolescents in their own care.
- EHR vendors designing patient portals and patient portals intended for adolescent use in particular may benefit from considering the types of information and functions that adolescents find useful.

Confidential: For Review Only

INTRODUCTION

An increasing number of healthcare providers are giving patients access to their electronic health records (EHR), including patient information such as clinical notes, diagnoses, and medication lists. The practice of enabling access to free-text notes written in the EHRs by healthcare professionals (HCPs) is sometimes called 'open notes'.[1] Commonly, patients gain online record access (ORA) via secure online portals. Patient-accessible electronic health records (PAEHR) are becoming increasingly expected among patients, even affecting patients' choice of healthcare provider. In particular, adolescents are growing up with expectations of digital convenience that also applies to healthcare.[2]

During early childhood, PAEHR portal systems can often allow for parental proxy access, where parents or guardians of the minor have access to the EHR of the child. However, adolescents needing healthcare face a hidden battle in balancing privacy and personal autonomy with the need for support from parents. Worries about sensitive health information (e.g. mental health issues and sexual health) being exposed to parents through the PAEHR can dissuade young people from accessing medical care.[3-4] The transition from adolescent to adult patienthood has therefore raised concerns among HCPs[5–7] and implementing online access to EHRs is especially controversial in this population.

The e-service for PAEHRs in Sweden is *Journalen*, available via the national web patient portal 1177.se, accessible to patients in all 21 Swedish regions.[8] According to publicly available statistics,[9] *Journalen* is being accessed by a steadily increasing number of users; approximately 1,360,000 unique users per month during 2022. Adult parent users have automatic access to their child's records in *Journalen*, but lose this access when the child turns 13, and adolescents gain their record access at 16. When an adolescent is between 13 and 16 years old, parents and adolescents can apply for extended access outside of the default. Adolescent patients access the same information as adults. The content includes for example a list of prescribed medications, lab results, growth curves, vaccinations, diagnoses, maternity care records, referrals, warnings, and treatment plans (Figure 1). However, availability differs according to region and connected health care providers (who have agreed to give access) (Figure 2). For example, psychiatric notes are shown in 17 of 21 regions.[10] Functions such as prescription renewal and appointment booking are conducted on the patient portal, yet externally from the PAEHR.

Figure 1. The patient-accessible electronic health record *Journalen* after log-in (manually translated), showing the functions and information available.[11] Service produced by Inera AB under the auspices of Swedish municipalities and regions.

Figure 2. The information shown in *Journalen* depending on region or health care provider (green squares) during the time of the survey.

Two literature reviews on pediatric patient portals found limited studies in this age group. One reviewed definition and utilization[12] while the other focused on use, views, and experiences among adolescents.[13] While expressing a lack of knowledge of how to use PAEHRs,[2,14] adolescents have stated a desire to receive such information from HCPs.[2,15] In a US study examining EHR access of adolescents' records by accounts divided into self, surrogate, and delegate, there was a significant increase of use during adolescence.[16] Furthermore, HCPs have stated the benefits of guiding adolescent patients to become more involved and activated in their care.[17-18] Research has mostly been observational or focused on adolescents' attitudes towards patient portals, rather than experiences. In observational studies, adolescents demonstrate the use of the same portal features as adults,[19] including lab results,[16,20-21] appointment review,[16,20-21] messaging,[16,20] and medications.[21] Existing studies of adolescent users have been limited to contexts of serious illness, and these adolescents report similar benefits of reading EHRs to those of adults, such as reduced anxiety, enhanced knowledge about their illness, ability to ask informed questions and reflecting more on their health,[22] and empowerment.[5,22] A benefit unique for adolescents concerned a supported transition into adulthood.[22] To date, no studies have systematically examined how adolescents more broadly experience ORA.

Study Aim

A recent scoping review concluded that with few exceptions (Australia, Canada, New Zealand, and the United Kingdom), the entire body of literature on adolescent access to EHRs had been carried out in the United States.[13] The present research explored reasons for reading the EHRs, the perceived usefulness of information and functions in a patient portal, and the association between frequency of use and HCP encouragement to read the EHR, among adolescents in Sweden.

METHODS

Study Design

Within the NORDeHEALTH research project,[23] an open survey designed to elicit the opinions and experiences of PAEHR users was conducted. The data were collected for three weeks from January to February 2022. Convenience sampling was used. Participants were notified about the survey when they logged into their records through the Swedish national PAEHR *Journalen*, by receiving a request to voluntarily participate in the survey, along with detailed information about the study. Accessing the survey without logging in was impossible. The participation request did not appear once the patient had completed the survey. Participants provided electronic consent and there was no financial incentive. Based on the definition of adolescence proposed by the United Nations (UN), youths aged 10-19 [24], only survey respondents aged 19 and younger were included for this paper. Due to an ethical-legal requirement of written parental consent for participants younger than 15 (The Act concerning the Ethical Review of Research Involving Humans[25]), which would complicate survey distribution, we opted to exclude those younger than 15 years. Therefore, the first survey item after consent concerned age and selecting *14 or younger* triggered information about exclusion and survey termination. The Checklist for Reporting Results of Internet E-Surveys (CHERRIES)[26] was used to report this study.

Data Collection

An anonymous questionnaire covering 83 items was designed in English and translated into Swedish. The questionnaire included a mixture of close- and open-ended questions with various response options (yes/no, multiple choice, and free-text form). The design and selection of items were informed by prior studies.[1,19,27] Answering close-ended questions was mandatory, while free-text questions were optional. The Swedish survey was distributed over 12 pages and some items were conditionally displayed based on responses to other items. Including conditional questions, the number of items ranged between 1-10 item per page. Respondents could move back and forth between survey sections, however did not have an option to review the filled form before submission. For this study, five items were included (see Figure 3) based on the study's aim. Three of these items used a 5-point Likert scale (ranging from disagree to agree), and two were close-ended questions. Data on use frequency was collected to enable comparison across groups. The items used in the current study are found in Figure 3, and answer options and item number can be found in Appendix A (English original and Swedish translation). The full survey and further details about the data collection procedure are found in a separate publication.[28] It should be noted that the availability of information and functions in *Journalen* varies across regions in Sweden, which may affect respondents' assessment. However, the items on perceived usefulness are formulated anticipatory (see Figure 3).

Figure 3. Survey items included in the study.

Statistical Analysis

Only completed questionnaires were analyzed. Aside from using descriptive statistics for calculating percentages for different response options, the Fisher's exact test was used to explore the difference in use frequency based on whether a HCP (physician, nurse, psychologist, physiotherapist, or other medical staff) had encouraged PAEHR use. Non-parametric tests were conducted due to the small sample size, and the significance level was set to 95%. The use frequency response options *10-20*

Confidential: For Review Only

times and *More than 20 times* were merged for analysis to represent more frequent use. The SPSS 28 software was used for analyses, and figures were produced using R.

Patient and public involvement

The survey was tested with the public before launch to assess its clarity and response burden, leading to minor adjustments. There was no other patient and public involvement in the design or conduct of the study.

RESULTS

Characteristics of the Participants

During the study period, 23,855 users opened the survey, and 15,867 (66.5%) started filling it in. Of these, 13,010 (82.0%) submitted the survey. Two (2/13 010, 0.02%) were 14 years old or younger and were excluded from the survey. Of the 13,008 respondents who completed the survey, 218 were 15-19 years old (1.7%). Of respondents aged between 15-19, 77.1% (168/218) identified as women, 17.9% (39/218) as men while 5.0% (11/218) selected Other. See data on survey completion in a previous publication[28] and descriptive statistics for all variables in Appendix B.

Experience with Accessing Records Online

Two out of five respondents had logged in more than 20 times (85/218, 39.0%) and one third had logged in 2-9 times (74/218, 33.9%) (see Table 1).

Table 1. Participants' frequency of accessing their health records online.

**Frequency of health record access, n (%)**	**Participants (N=218)**
This is my first time	11 (5)
2 to 9 times	74 (33.9)
10-20 times	48 (22)
More than 20 times	85 (39)

Encouragement to Read the Health Records Online

One survey question focused on whether respondents had been encouraged or reminded by anyone or any media source to read their records online. The most common response was *nobody encouraged me* (118/218, 54.1%), see Figure 4. Of four respondents that chose *Other*, one respondent provided a free-text description, citing "myself". Among the 47 individuals (47/218, 21.6%) who had been encouraged by some type of healthcare provider (HCP), 36 (76.6%) were frequent users of the patient portal, which is higher than those who had not been encouraged (97/171, 56.7%) (p=.043).

Figure 4. Encouragement to read health records online. Because respondents could select multiple items, the total number can exceed 100%. Legend: *Types of HCPs.

Reasons for Reading the Health Records Online

Four reasons were chosen by more than half of respondents: *out of general curiosity* (170/218, 78.0%), *to get an overview of one's medical history and/or treatment* (159/218, 72.9%), *to ensure having understood what the physician or nurse said* (149/218, 68.3%), and *to improve one's understanding for one's health issue* (138/218, 63.3%) (see Figure 5). Of 50 respondents who selected *Other*, 16 provided free-text comments. The comments included ensuring accuracy, memory reinforcement, poor notification frequency (i.e. logging in to check for new information due to lack of notifications), specification of sought information (e.g., test results, Covid-19 vaccinations), and retrieving a record copy for monetary activity compensation.

Figure 5. Adolescents' reasons for reading health records online.

Usefulness of Portal Information and Function

All information types were considered useful by more than 3/4 of participants, see Figure 6.

Confidential: For Review Only

Figure 6. Adolescents' perceived usefulness of different types of patient portal information.

Perceived usefulness of various functions was also examined, most of which do not currently exist in *Journalen*. Most functions were perceived as useful by a majority of respondents, see Figure 7. Yet, four functions were considered useful by less than half of respondents: *accessing information and managing services for children* and for *family members*, *contributing information about expectations for the health care visit*, and *blocking specific clinical notes from certain HCPs/medical staff*. Of 24 participants who wrote a comment under *Other*, 7 referred to a specific function. Comments concerned the ability to view radiology images (median=4.5), diagnosis (median=3.5), dentistry records (median=5), ability to read children's records when under 18 (median=5), records from childhood (median=5), and to view appointments from gynecologic care (median=5). Other free-text answers were non-responses or unspecified. *Medical certificates and other certificates* concerned certificates from HCPs, such as COVID-19 vaccination and for insurance and sick leave.

Figure 7. Adolescents' perceived usefulness of patient portal functions.

DISCUSSION

In our national survey of PAEHR experiences in Sweden, adolescents perceived all proposed clinical information as consistently useful. Ratings for portal functions varied. Common reasons for reading records were curiosity and to get an overview of medical history. Sharing clinical information was the least common reason for accessing the record. Encouragement by HCPs to read records was associated with higher use. The reasons for reading records align with previous research on adults[19] and US adolescents.[22]

While all information types were rated highly on usefulness, test results were considered the most useful. This result accords with earlier research on adolescents,[16,20-21] but also with research on parents[29-30] and general adult populations.[19] Unsurprisingly, adolescents rated the ability to manage family members or children low. The function perceived as the third most useful was the ability to order and manage various certificates. This was likely associated with the timing of survey distribution, as the ongoing COVID-19 pandemic required Swedish citizens to present COVID-19 certificates for traveling and attending events. The boost of PAEHR use as a result of the COVID-19 pandemic has been mentioned previously.[31] Related to this, it is somewhat unexpected that immunizations were rated among the least useful types of information. However, Swedish regions differed in managing COVID-19 vaccination through the national e-service or other apps, and not all regions displayed information on vaccinations. Another finding was that adolescents do not read their PAEHRs to share information. This finding is consistent with a previous survey study based in Canada, where adolescents without access to their records anticipated not being likely to share information from the records with others.[32]

Encouragement from HCPs was linked to higher use, indicating the influence of social factors in eHealth adoption.[33] Another possible explanation is that those with more active medical issues needed to review their health record more frequently. Encouragement from HCPs and family/friends has increased PAEHR use in adults,[34] suggesting that this may be an important factor influencing adoption and use also in adolescents. Lack of HCP encouragement aligns with research on adults in the United States[35] and Sweden,[36] and may stem from concerns about medical literacy, increased workload, and uncertainty about visible information.[2,5,37–39] Not encouraging adolescents may lead to a missed opportunity for early patient involvement.[17] Increased PAEHR usage among adolescents can motivate policy-making stakeholders and EHR vendors to improve patient portals for this population.[5]

This study has limitations. Response biases in self-report surveys are unknown. Survey availability on the national patient portal may have influenced respondents' decisions to respond based on their competence and skills in using the service, potentially limiting the generalizability of the findings and causing positive bias as most adolescent respondents were frequent users. However, we opted to use convenience sampling as part of an exploratory approach. The majority of participants were women, which is consistent with adult and previous adolescent PAEHR users but represents a

Confidential: For Review Only

strong gender imbalance. Only 1.7% of the total respondents were aged 15-19, possibly due to younger individuals being less affected by health conditions and less engaged in personal health management. The survey was also designed for adults, and adolescents may have struggled to understand some questions. Furthermore, the reliability and validity of the survey utilized in this study have not been formally evaluated. While the survey was designed to capture the intended data, a comprehensive assessment of its psychometric properties was beyond the scope of this research. Still, the survey was tested with the public, as stated under Patient and public involvement.

The present results provide a broader understanding of adolescents reading their EHRs, however further work is required to establish the viability of the findings in other settings. Several questions remain, relating to for example eHealth literacy and confidentiality. It should be noted that the current study included only a subset of items from a larger survey, and there is an intention to publish additional findings on the adolescent population, on participants' views about security and privacy. Regarding our research methodology, we offer a few suggestions for future work. First, frequency of use was defined by how many times (first time, 2-9 times etc.) the users accessed their records in the past 12 months. Another approach is to ask how often users access their records (daily, weekly, etc.), which allows for an understanding of the visit regularity. Second, further research may benefit from providing a clearer distinction between a specific PAEHR service and patients' ORA more broadly. Lastly, including a larger and more evenly gender-distributed sample and specific patient groups with chronic diseases could furnish greater insights into adolescent users' experiences.

Data availability statement

Data are available from the corresponding author on reasonable request.

Ethics statements

Patient consent for publication

Electronic consent obtained from survey respondents.

Ethics approval

Ethical approval for this study was obtained from the Regional Ethical Review Board in Uppsala, Sweden (EPN 2021/05229). All respondents received study information and provided informed consent.

Abbreviations

EHR: electronic health record
HCP: healthcare professional
ORA: online record access
PAEHR: patient-accessible electronic health record

FOOTNOTES

Contributors: JH wrote the manuscript, created the tables, and designed the figures. MH, CB, IS, JM, ÅC, and HR contributed to the study design. All authors read the manuscript, provided feedback, and approved the final draft for submission.

Funding: This work was supported by NordForsk (grant number #100477) and the Swedish Research Council for Health, Working Life and Welfare (FORTE) (grant number #2020-01229).

Competing interests: None.

Patient and public involvement: Patients and/or the public were involved in survey testing but otherwise not involved in the design, or conduct, or reporting, or dissemination plans of this research.

Provenance and peer review: Not commissioned; externally peer reviewed.

Confidential: For Review Only

Figure captions:
Figure 1. The patient-accessible electronic health record *Journalen* after log-in (manually translated), showing the functions and information available.[11] Service produced by Inera AB under the auspices of Swedish municipalities and regions.
Figure 2. The information shown in *Journalen* depending on region or health care provider (green squares) during the time of the survey.
Figure 3. Survey items included in the study.
Figure 4. Encouragement to read health records online. Because respondents could select multiple items, the total number can exceed 100%. Legend: *Types of HCPs.
Figure 5. Adolescents' reasons for reading health records online.
Figure 6. Adolescents' perceived usefulness of different types of patient portal information.
Figure 7. Adolescents' perceived usefulness of patient portal functions.

REFERENCES

1. Walker J, Leveille S, Bell S, et al. OpenNotes After 7 Years: Patient Experiences With Ongoing Access to Their Clinicians' Outpatient Visit Notes. J Med Internet Res. 2019;21(5):e13876. doi:10.2196/13876
2. Beaton L, Williams I, Sanci L. Exploring adolescent and clinician perspectives on Australia. Aust J Prim Health. Published online March 15, 2021. doi:10.1071/PY20169
3. Bayer R, Santelli J, Klitzman R. New Challenges for Electronic Health Records: Confidentiality and Access to Sensitive Health Information About Parents and Adolescents. JAMA. 2015;313(1):29. doi:10.1001/jama.2014.15391
4. Ford CA. Foregone Health Care Among Adolescents. JAMA. 1999;282(23):2227. doi:10.1001/jama.282.23.2227
5. Carlson JL, Goldstein R, Buhr T, Buhr N. Teenager, parent, and clinician perspectives on the electronic health record. Pediatrics. 2020;145(3). doi:10.1542/peds.2019-0193
6. Goldstein RL, Anoshiravani A, Svetaz MV, Carlson JL. Providers' Perspectives on Adolescent Confidentiality and the Electronic Health Record: A State of Transition. J Adolesc Health. 2020;66:296-300. doi:10.1016/j.jadohealth.2019.09.020
7. Stablein T, Loud KJ, DiCapua C, Anthony DL. The Catch to Confidentiality: The Use of Electronic Health Records in Adolescent Health Care. J Adolesc Health. 2018;62(5):577-582. doi:10.1016/j.jadohealth.2017.11.296
8. Hägglund M, Scandurra I. Patients' Online Access to Electronic Health Records: Current Status and Experiences from the Implementation in Sweden. MEDINFO 2017 Precis Healthc Inform. Published online 2017:723-727. doi:10.3233/978-1-61499-830-3-723
9. Statistik för Ineras tjänster [Statistics for Inera's services]. Inera AB. Accessed August 25, 2023. https://www.inera.se/tjanster/statistik-for-ineras-tjanster/
10. Bärkås A, Scandurra I, Rexhepi H, Blease C, Cajander Å, Hägglund M. Patients' Access to Their Psychiatric Notes: Current Policies and Practices in Sweden. Int J Environ Res Public Health. 2021;18(17):9140. doi:10.3390/ijerph18179140
11. Journalen. Inera. Published December 2022. Accessed December 8, 2022. https://journalen.1177.se/
12. Bush RA, Connelly CD, Fuller M, Pérez A. Implementation of the Integrated Electronic Patient Portal in the Pediatric Population: A Systematic Review. Telemed J E-Health Off J Am Telemed Assoc. 2016;22(2):144-152. doi:10.1089/tmj.2015.0033
13. Hagström J, Blease C, Haage B, Scandurra I, Hansson S, Hägglund M. Use, Views of and Experiences of Web-Based Access to Electronic Health Records for Children, Adolescents, and Parents: Scoping Review. J Med Internet Res. Published online 2022. doi:10.2196/40328
14. Miklin DJ, Vangara SS, Delamater AM, Goodman KW. Understanding of and Barriers to Electronic Health Record Patient Portal Access in a Culturally Diverse Pediatric Population. JMIR Med Inform. 2019;7(2):e11570. doi:10.2196/11570

Confidential: For Review Only

15. Bergman DA, Brown NL, Wilson S. Teen Use of a Patient Portal: A Qualitative Study of Parent and Teen Attitudes. Perspect Health Inf Manag. 2008;5:13.
16. Steitz BD, Cronin RM, Davis SE, Yan E, Jackson GP. Long-term patterns of patient portal use for pediatric patients at an academic medical center. Appl Clin Inform. 2017;8(3):779-793. doi:10.4338/ACI-2017-01-RA-0005
17. Bourgeois FC, DesRoches CM, Bell SK. Ethical Challenges Raised by OpenNotes for Pediatric and Adolescent Patients. Pediatrics. 2018;141(6):e20172745. doi:10.1542/peds.2017-2745
18. Lam BD, Bourgeois F, DesRoches CM, Dong Z, Bell SK. Attitudes, experiences, and safety behaviours of adolescents and young adults who read visit notes: Opportunities to engage patients early in their care. Future Heal J. 2021;8(3):e585-e592. doi:10.7861/fhj.2021-0118
19. Moll J, Rexhepi H, Cajander Å, et al. Patients' Experiences of Accessing Their Electronic Health Records: National Patient Survey in Sweden. J Med Internet Res. 2018;20(11):e9492. doi:10.2196/jmir.9492
20. Calman N, Pfister HR, Lesnewski R, Hauser D, Shroff N. Electronic Access to Adolescents' Health Records: Legal, Policy, and Practice Implications. Vol 22.; 2014:11-14. https://www.aafp.org/fpm/2015/0300/p11.html
21. Thompson LA, Martinko T, Budd P, Mercado R, Schentrup AM. Meaningful use of a confidential adolescent patient portal. J Adolesc Health. 2016;58(2):134-140. doi:10.1016/j.jadohealth.2015.10.015
22. Hong MK, Wilcox L, Feustel C, Wasileski-Masker K, Olson TA, Simoneaux SF. Adolescent and Caregiver use of a Tethered Personal Health Record System. AMIA Annu Symp Proc AMIA Symp. 2016;2016:628-637.
23. Hägglund M. Nordic countries lead new initiative on patient access to EHRs. TheBMJopinion. Published May 18, 2021. Accessed January 16, 2022. https://blogs.bmj.com/bmj/2021/05/18/maria-hagglund-nordic-countries-lead-new-initiative-on-patient-access-to-ehrs/
24. "Definition of Youth." United Nations Department of Economic and Social Affairs (UNDESA).
25. Riksdagsförvaltningen. 2003:460.; 2003. Accessed August 3, 2022. https://www.riksdagen.se/sv/dokument-lagar/dokument/svensk-forfattningssamling/lag-2003460-om-etikprovning-av-forskning-som_sfs-2003-460
26. Eysenbach G. Improving the Quality of Web Surveys: The Checklist for Reporting Results of Internet E-Surveys (CHERRIES). J Med Internet Res. 2004;6(3):e34. doi:10.2196/jmir.6.3.e34
27. Zanaboni P, Kummervold PE, Sørensen T, Johansen MA. Patient use and experience with online access to electronic health records in Norway: Results from an online survey. J Med Internet Res. 2020;22(2). doi:10.2196/16144
28. Hägglund M, Kharko A, Hagström J, et al. The NORDeHEALTH 2022 Patient Survey: A cross-sectional survey of national patient portal users in Norway, Sweden, Finland, and Estonia. J Med Internet Res. 2023;25:e47573. doi:10.2196/47573
29. Ahlers-Schmidt CR, Nguyen M. Parent Intention to Use a Patient Portal as Related to Their Children Following a Facilitated Demonstration. Telemed E-Health. 2013;19(12):979-981. doi:10.1089/tmj.2013.0041
30. Thompson LA, Black EW, Saliba H, Schentrup AM. Parents' knowledge of and opinions about healthcare laws and technology in primary care. Inform Prim Care. 2012;20(1):69-74. doi:10.14236/jhi.v20i1.49
31. Blease C, Salmi L, Hägglund M, Wachenheim D, DesRoches C. COVID-19 and Open Notes: A New Method to Enhance Patient Safety and Trust. JMIR Ment Health. 2021;8(6):e29314. doi:10.2196/29314
32. Frazer C, Ratchford F, Roch J. Enabling Adolescent Electronic Access to Personal Health Information. Stud Health Technol Inform. 2017;234:115-119.
33. Venkatesh V, Morris MG, Davis GB, Davis FD. User Acceptance of Information Technology: Toward a Unified View. MIS Q. 2003;27(3):425-478. doi:10.2307/30036540

34. Mukhopadhyay S, Basak R, Carpenter D, Reithel BJ. Patient use of online medical records: an application of technology acceptance framework. Inf Comput Secur. 2019;28(1):97-115. doi:10.1108/ICS-07-2019-0076
35. DesRoches CM, Leveille S, Bell SK, et al. The Views and Experiences of Clinicians Sharing Medical Record Notes With Patients. JAMA Netw Open. 2020;3(3):e201753. doi:10.1001/jamanetworkopen.2020.1753
36. Rexhepi H, Moll J, Huvila I. Online electronic healthcare records: Comparing the views of cancer patients and others. Health Informatics J. 2020;26(4):2915-2929. doi:10.1177/1460458220944727
37. Hagström J, Blease C, Haage B, Scandurra I, Hansson S, Hägglund M. Views, Use, and Experiences of Web-Based Access to Pediatric Electronic Health Records for Children, Adolescents, and Parents: Scoping Review. J Med Internet Res. 2022;24(11):e40328. doi:10.2196/40328
38. Grünloh C, Myreteg G, Cajander Å, Rexhepi H. "Why Do They Need to Check Me?" Patient Participation Through eHealth and the Doctor-Patient Relationship: Qualitative Study. J Med Internet Res. 2018;20(1):e8444. doi:10.2196/JMIR.8444
39. Petersson J, Backman C. Patient-accessible online health records: Reconfigurations of clinical rhythms and doctors' front- and backstage spaces. Soc Sci Med. 2022;292:114635. doi:10.1016/j.socscimed.2021.114635

Confidential: For Review Only

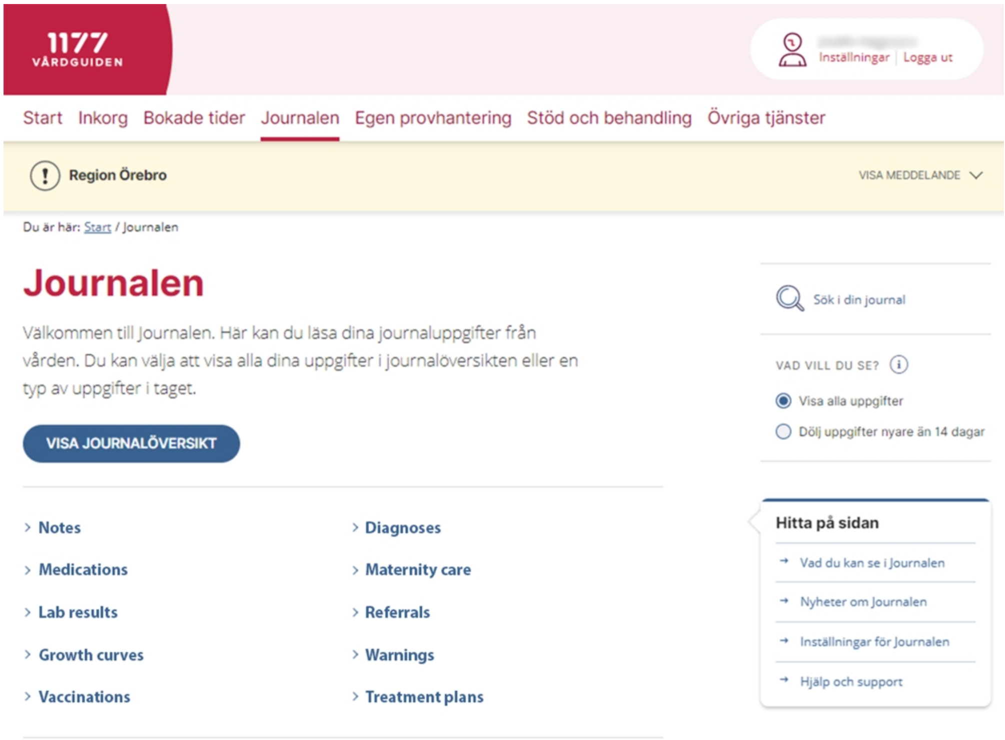

Figure 1. The patient-accessible electronic health record Journalen after log-in (manually translated), showing the functions and information available.[11] Service produced by Inera AB under the auspices of Swedish county councils and regions.

578x427mm (300 x 300 DPI)

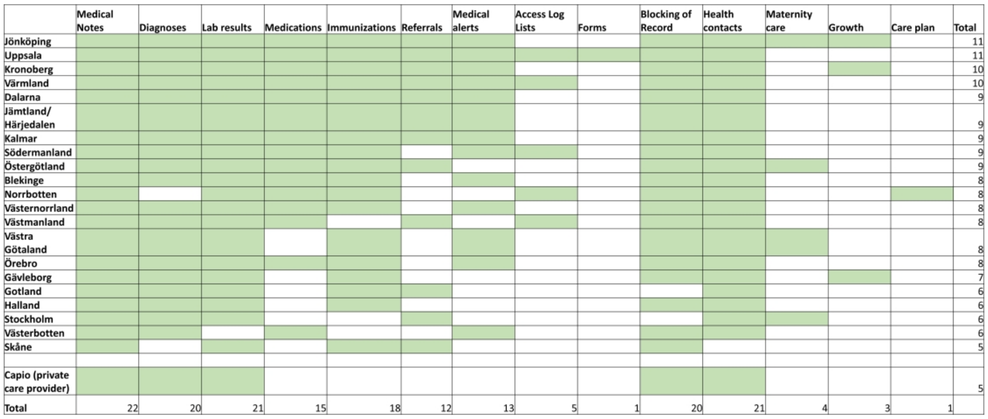

	Medical Notes	Diagnoses	Lab results	Medications	Immunizations	Referrals	Medical alerts	Access Log Lists	Forms	Blocking of Record	Health contacts	Maternity care	Growth	Care plan	Total
Jönköping															11
Uppsala															11
Kronoberg															10
Värmland															10
Dalarna															9
Jämtland/ Härjedalen															9
Kalmar															9
Södermanland															9
Östergötland															9
Blekinge															8
Norrbotten															8
Västernorrland															8
Västmanland															8
Västra Götaland															8
Örebro															8
Gävleborg															7
Gotland															6
Halland															6
Stockholm															6
Västerbotten															6
Skåne															5
Capio (private care provider)															5
Total	22	20	21	15	18	12	13	5	1	20	21	4	3	1	

Figure 2. The information shown in Journalen depending on region or health care provider (green squares) during the time of the survey.

758x320mm (300 x 300 DPI)

1. How often do you think that you have accessed your health record during the last 12 months?
2. Did any of the following encourage or remind you to read your health record?
   (check all that apply)
3. Please indicate how much you disagree or agree with the following statement:
   I read my health record online… [reasons for reading]
4. How useful would it be to have access to the following information in the portal?
5. How useful would it be to have access to the following functions in the portal?

Figure 3. Survey questions included in the study.

520x176mm (300 x 300 DPI)

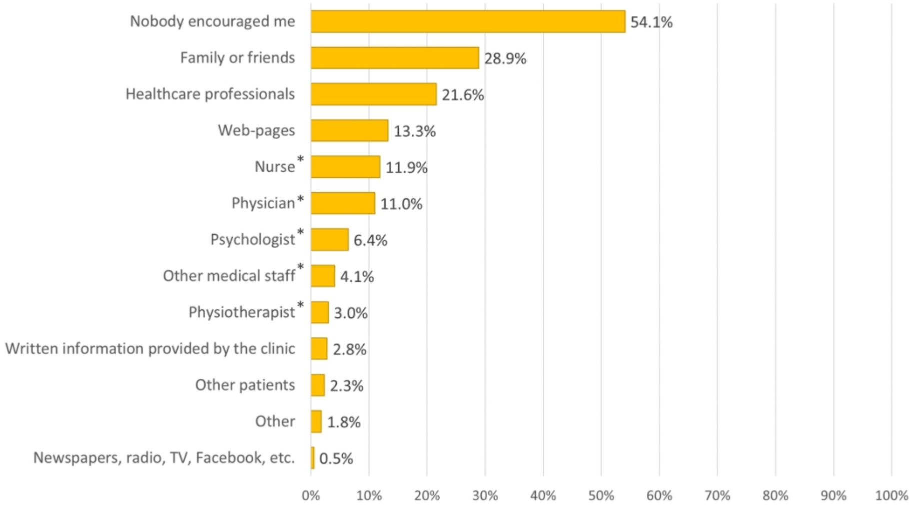

Figure 4. Encouragement to read health records online. Because respondents could select yes for multiple items, the total number can exceed 100%. Legend: *Types of HCPs.

478x250mm (300 x 300 DPI)

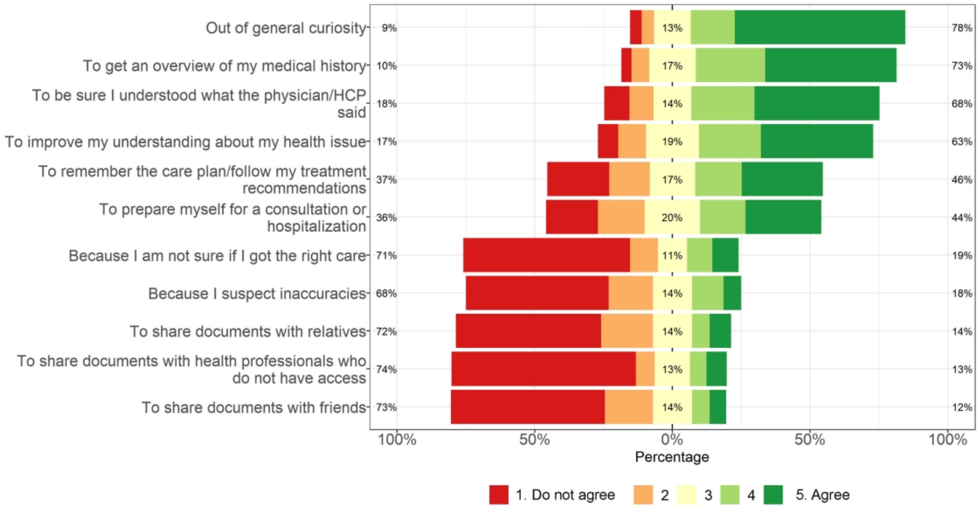

Figure 5. Adolescents' reasons for reading health records online.

292x152mm (300 x 300 DPI)

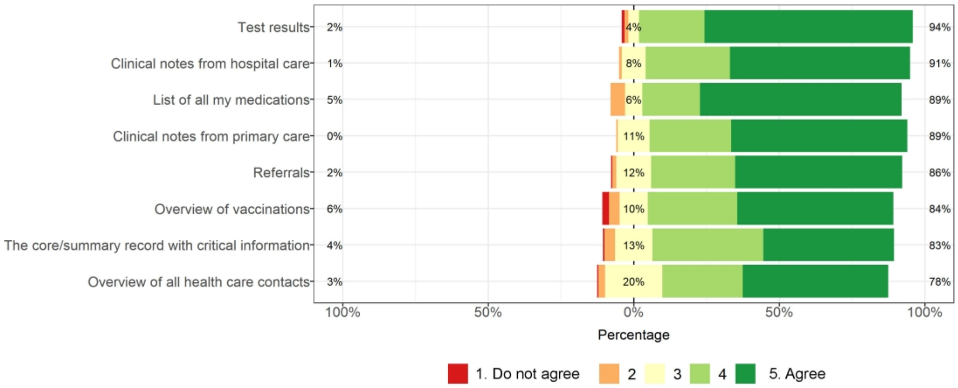

Figure 6. Adolescents’ perceived usefulness of different types of patient portal information.

279x112mm (300 x 300 DPI)

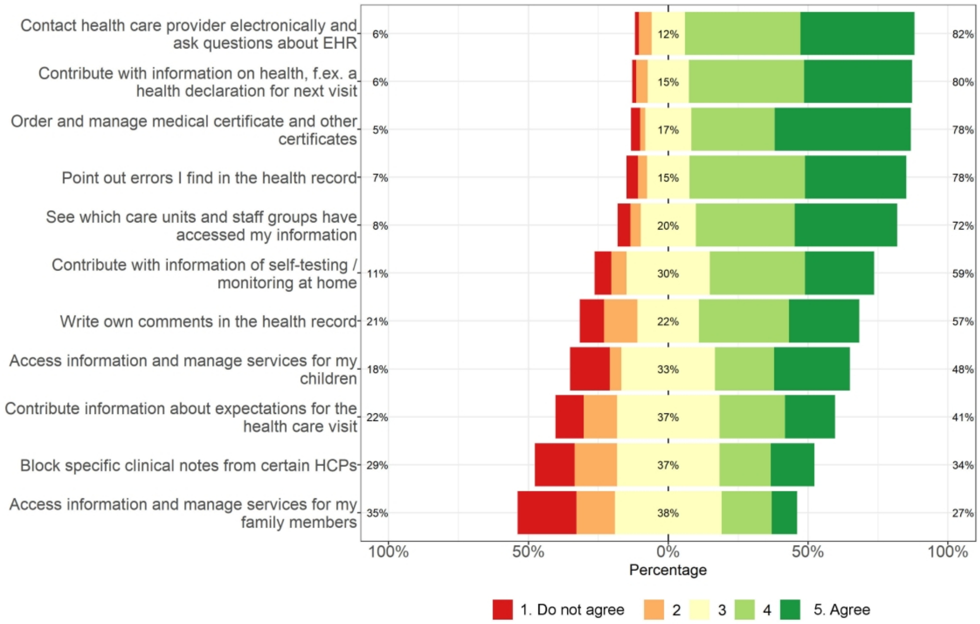

Figure 7. Adolescents' perceived usefulness of patient portal functions. Legend: *Functions that exist in all or some regions in Sweden. Only adult users over 18 years old have access to their children's records.

279x177mm (300 x 300 DPI)

Appendix A

Table 1. Original survey created in English

Number in survey	Question	Response alternatives
6	**How often do you think that you have accessed your health record during the last 12 months?**	1 - This is my first time<br>2 - 2 to 9 times<br>3 - 10 to 20 times<br>4 - More than 20 times
7	**Did any of the following encourage or remind you to read your health record?**	HCPs: Physician<br>HCPs: Nurse<br>HCPs: Psychologist<br>HCPs: Physiotherapist<br>HCPs: Other medical staff<br>Written information provided by the hospital/healthcare facility /clinic<br>Family or friends<br>Web-pages, such as the National health portal web-page, etc.<br>Newspapers, radio, TV, Facebook, etc.<br>Other patients<br>Nobody encouraged me<br>Other
8	Please indicate how much you disagree or agree with the following statement:<br>**I read my health record online…**	1 - Disagree,<br>2, 3, 4,<br>5 - Agree
	out of general curiosity	
	to improve my understanding about my health issue	
	to prepare myself for a consultation or hospitalization	
	to get an overview of my medical history and/or treatment	
	to be sure I understood what the physician/healthcare professional said	
	to remember the care plan/follow my treatment recommendations	
	because I suspect inaccuracies	
	to share documents with relatives	
	to share documents with friends	
	to share documents with health professionals who do not have access	
	because I am not sure if I got the right care	
	Other, please specify	

38	**How useful would it be to have access to the following information in the portal?**		1 - Not useful at all<br>2 - Not very useful<br>3 - Neither useful nor useless<br>4 - Useful<br>5 - Very useful
		The core/summary record with critical information	
		Referrals (content and how it is handled in care)	
		List of all pharmaceuticals/my medications	
		Overview of vaccinations	
		Results of tests	
		Clinical notes from primary care	
		Clinical notes from hospital care	
		Overview of all health care contacts	
39	**How useful would it be to have access to the following functions in the portal?**		1 - Not useful at all<br>2 - Not very useful<br>3 - Neither useful nor useless<br>4 - Useful<br>5 - Very useful
		Ability to access information and manage services for my children	
		Ability to access information and manage services for my family members	
		See which care units and staff groups have accessed my information (see log data)	
		Ability to block certain medical records from access by other medical staff	
		Ability to contact health care provider electronically and ask questions about medical record	
		Ability to point out errors I find in the health record	
		Ability to write own comments to text in the health record	
		Contribute with information on health, for example by providing a health declaration for next visit	
		Contribute with information of self-testing/monitoring at home	
		Contribute information about expectations for the health care visit	
		Ability to order and manage medical certificate and other certificates	
		Other	

Table B2. Swedish translation of survey

Number in survey	Question		Response alternatives
6	**Hur ofta har du läst i din journal under de senaste 12 månaderna?**		1 - Det här är första gången<br>2 - 2-9 gånger<br>3 - 10-20 gånger<br>4 - Mer än 20 gånger
7	**Har du blivit uppmuntrad eller påmind att läsa din journal av någon/något av följande:**		Vårdpersonal: Läkare<br>Vårdpersonal: Sjuksköterska<br>Vårdpersonal: Psykolog<br>Vårdpersonal: Fysioterapeut/sjukgymnast<br>Vårdpersonal: Annan vårdpersonal<br>Skriftlig information från sjukhuset/vårdinrättning/klinik<br>Familj eller vänner<br>Webbsidor, såsom 1177.se Vårdguiden, etc.<br>Tidningar, radio, TV, Facebook, etc.<br>Andra patienter<br>Ingen har uppmuntrat eller påmint mig<br>Annat
8	Markera i vilken utsträckning du instämmer med följande påståenden:<br>**Jag läser min journal på internet…**		1 - Instämmer inte, 2, 3, 4,<br>5 - Instämmer
		Av nyfikenhet	
		För att förbättra min förståelse för mitt hälsotillstånd	
		För att förbereda mig själv för ett vårdbesök eller sjukhusvistelse	
		För att få en överblick av min hälsohistoria och/eller bakgrund	
		För att försäkra mig om att jag förstått vad läkaren/vårdpersonalen sagt	
		För att komma ihåg vårdplanen/följa mina behandlingsrekommendationer	
		För att jag misstänker felaktigheter	
		För att dela med mig av information till släkt	
		För att dela med mig av information till vänner	
		För att dela med mig av information till vårdpersonal som inte har tillgång till min journal	
		För att jag är osäker på om jag fått rätt vård	
		Annat, specificera gärna	

38	**Hur användbart skulle det vara för dig att ha tillgång till följande information i *Journalen*?**		1 - Inte alls användbart<br>2 - Inte särskilt användbart<br>3 - Varken eller<br>4 - Användbart<br>5 - Väldigt användbart
		En sammanfattning av journalen med viktig information	
		Remisser (innehåll och status)	
		Lista över mina läkemedel/mediciner	
		Vaccinationer	
		Provsvar	
		Journalanteckningar från primärvården	
		Journalanteckningar från specialistvården	
		Överblick av samtliga kontakter med vården	
39	**Hur användbart skulle det vara för dig att ha tillgång till följande funktioner i *Journalen*?**		1 - Inte alls användbart<br>2 - Inte särskilt användbart<br>3 - Varken eller<br>4 - Användbart<br>5 - Väldigt användbart
		Möjlighet att ta del av information och hantera tjänster för mina barn	
		Möjlighet att ta del av information och hantera tjänster för andra familjemedlemmar	
		Möjlighet att kunna blockera specifika journalanteckningar för viss vårdpersonal	
		Se vilka vårdinrättningar och personal som har läst i min journal (loggar)	
		Möjlighet att kunna kontakta vårdpersonal online för att ställa frågor om min journal	
		Möjlighet att påpeka felaktigheter i journalen	
		Möjlighet att skriva egna kommentarer i journalen	
		Möjlighet att bidra med information om min hälsa, t ex genom att fylla i en hälsodeklaration, inför ett besök	
		Möjlighet att bidra med information från självtest/monitorering hemma	
		Möjlighet att bidra med information om mina förväntningar på vårdbesöket	
		Möjlighet att beställa och hantera sjukskrivning, medicinsk intyg och andra dokument (vaccinationsintyg t ex)	
		Annat	

Appendix B

Detailed results from the survey questions chosen for this study.

Encouragement to Read.

Did any of the following encourage or remind you to read your health record? n (%)	Frequency
HCPs: Physician	24 (11.0)
HCPs: Nurse	26 (11.9)
HCPs: Psychologist	14 (6.4)
HCPs: Physiotherapist	6 (2.8)
HCPs: Other medical staff	9 (4.1)
Written information provided by the hospital/healthcare facility/clinic	12 (5.5)
Family or friends	63 (28.9)
Web-pages, such as the National health portal web-page, etc.	29 (13.3)
Newspapers, radio, TV, Facebook, etc.	1 (0.5)
Other patients	5 (2.3)
Nobody encouraged me	118 (54.1)
Other	4 (1.8)
**Encouraged by HCP or not, n (%)**	
Encouraged by HCP	47 (21.6)
Not encouraged by HCP	171 (78.4)

Reasons for reading health records online.

I read my health record online…, n (%)	1. Disagree	2	3	4	5. Agree
… out of general curiosity	9 (4.1)	10 (4.6)	29 (13.3)	35 (16.1)	135 (61.9)
… to improve my understanding about my health issue	16 (7.3)	22 (10.1)	42 (19.3)	49 (22.5)	89 (40.8)
… to prepare myself for a consultation or hospitalization	41 (18.8)	37 (17.0)	44 (20.2)	36 (16.5)	60 (27.5)
… to get an overview of my medical history and/or treatment	8 (3.7)	14 (6.4)	37 (17.0)	55 (25.2)	104 (47.7)
… to be sure I understood what the physician/healthcare professional said	20 (9.2)	19 (8.7)	30 (13.8)	50 (22.9)	99 (45.4)
… to remember the care plan/follow my treatment recommendations	49 (22.5)	32 (14.7)	36 (16.5)	37 (17.0)	64 (29.4)
… because I suspect inaccuracies	113 (51.8)	35 (16.1)	31 (14.2)	25 (11.5)	14 (6.4)
… to share documents with relatives	115 (52.8)	41 (18.8)	31 (14.2)	14 (6.4)	17 (7.8)
… to share documents with friends	122 (56.0)	38 (17.4)	31 (14.2)	14 (6.4)	13 (6.0)
… to share documents with healthcare professionals who do not have access	146 (67.0)	15 (6.9)	28 (12.8)	13 (6.0)	16 (7.3)
… because I am not sure if I got the right care	132 (60.6)	22 (10.1)	23 (10.6)	20 (9.2)	21 (9.6)
Other	168 (77.1)	20 (9.2)	5 (2.3)	3 (1.4)	22 (10.1)

Perceived usefulness of various types of information.

Information	Not useful at all	Not very useful	Neither useful nor useless	Useful	Very useful
The core/summary record with critical information	1 (0.5)	8 (3.7)	28 (12.8)	83 (38.1)	98 (45.0)
Referrals (content and how it is handled in care)	1 (0.5)	3 (1.4)	26 (11.9)	63 (28.9)	125 (11.9)
List of all pharmaceuticals / my medications	0 (0)	11 (5.0)	13 (6.0)	43 (19.7)	151 (69.3)
Overview of vaccinations	5 (2.3)	8 (3.7)	21 (9.6)	67 (30.7)	117 (53.7)
Results of tests (not available in all regions)	2 (0.9)	3 (1.4)	8 (3.7)	49 (22.5)	156 (71.6)
Clinical notes from primary care	0 (0)	1 (0.5)	24 (11.0)	61 (28.0)	132 (60.6)
Clinical notes from hospital care	0 (0)	2 (0.9)	18 (8.3)	63 (28.9)	135 (61.9)
Overview of all health care contacts	1 (0.5)	5 (2.3)	43 (19.7)	60 (27.5)	109 (50)

Perceived usefulness of various functions.

Function	Not useful at all	Not very useful	Neither useful nor useless	Useful	Very useful
Ability to access information and manage services for my children (not available in all regions).	31 (14.2)	9 (4.1)	73 (33.5)	46 (21.1)	59 (27.1)
Ability to access information and manage services for my family members.	46 (21.1)	30 (13.8)	83 (38.1)	39 (17.9)	20 (9.2)
Ability to block specific clinical notes from certain healthcare professionals/medical staff.	31 (14.2)	33 (15.1)	80 (36.7)	40 (18.3)	34 (15.6)
See which care units and staff groups have accessed my information (see log data).	10 (4.6)	8 (3.7)	43 (19.7)	77 (35.3)	80 (36.7)
Ability to contact health care provider electronically and ask questions about medical record.	3 (1.4)	10 (4.6)	26 (11.9)	90 (41.3)	89 (40.8)
Ability to point out errors I find in the health record.	9 (4.1)	7 (3.2)	33 (15.1)	90 (41.3)	79 (36.2)
Ability to write own comments to text in the health record.	19 (8.7)	26 (11.9)	48 (22.0)	70 (32.1)	55 (25.2)
Contribute with information on health, for example by providing a health declaration for next visit.	3 (1.4)	9 (4.1)	32 (14.7)	90 (41.3)	84 (38.5)
Contribute with information of self-testing/monitoring at home.	13 (6.0)	12 (5.5)	65 (29.8)	74 (33.9)	54 (24.8)
Contribute information about expectations for the health care visit.	22 (10.1)	26 (11.9)	80 (36.7)	51 (23.4)	39 (17.9)
Ability to order and manage medical certificate and other certificates.	7 (3.2)	4 (1.8)	36 (16.5)	65 (29.8)	106 (48.6)

BMJ Paediatrics Open

Adolescents' reasons for accessing their health records online, perceived usefulness, and experienced provider encouragement: A national survey in Sweden

Journal:	*BMJ Paediatrics Open*
Manuscript ID	bmjpo-2023-002258.R4
Article Type:	Original research
Date Submitted by the Author:	21-Feb-2024
Complete List of Authors:	Hagström, Josefin; Uppsala Universitet, Department of Women's and Children's Health; Uppsala University Hospital, Medtech Science & Innovation Centre<br>Blease, Charlotte; Uppsala Universitet, Department of Women's and Children's Health; Harvard Medical School, Beth Israel Deaconess Medical Center<br>Scandurra, Isabella; Örebro University, School of Business<br>Moll, Jonas; Örebro University, School of Business<br>Cajander, Åsa; Uppsala Universitet, Department of Information Technology<br>Rexhepi, Hanife; University of Skövde, School of Informatics<br>Hägglund, Maria; Uppsala Universitet, Department of Women's and Children's Health; Uppsala University Hospital, Medtech Science & Innovation Centre
Keywords:	Adolescent Health, Health services research, Data Collection

SCHOLARONE™
Manuscripts

I, the Submitting Author has the right to grant and does grant on behalf of all authors of the Work (as defined in the below author licence), an exclusive licence and/or a non-exclusive licence for contributions from authors who are: i) UK Crown employees; ii) where BMJ has agreed a CC-BY licence shall apply, and/or iii) in accordance with the terms applicable for US Federal Government officers or employees acting as part of their official duties; on a worldwide, perpetual, irrevocable, royalty-free basis to BMJ Publishing Group Ltd ("BMJ") its licensees and where the relevant Journal is co-owned by BMJ to the co-owners of the Journal, to publish the Work in this journal and any other BMJ products and to exploit all rights, as set out in our licence.

The Submitting Author accepts and understands that any supply made under these terms is made by BMJ to the Submitting Author unless you are acting as an employee on behalf of your employer or a postgraduate student of an affiliated institution which is paying any applicable article publishing charge ("APC") for Open Access articles. Where the Submitting Author wishes to make the Work available on an Open Access basis (and intends to pay the relevant APC), the terms of reuse of such Open Access shall be governed by a Creative Commons licence – details of these licences and which Creative Commons licence will apply to this Work are set out in our licence referred to above.

Other than as permitted in any relevant BMJ Author's Self Archiving Policies, I confirm this Work has not been accepted for publication elsewhere, is not being considered for publication elsewhere and does not duplicate material already published. I confirm all authors consent to publication of this Work and authorise the granting of this licence.

Confidential: For Review Only

TITLE PAGE

Article title: Adolescents' reasons for accessing their health records online, perceived usefulness, and experienced provider encouragement: A national survey in Sweden

Author information:
Corresponding author:
Josefin Hagström, MSc[1,2] (ORCID: 0000-0003-2835-0259)
Department of Women's and Children's Health, Uppsala University, Uppsala, Sweden

josefin.hagstrom@uu.se
+46184715080

Co-authors:
Charlotte Blease PhD[1,3] (ORCID: 0000-0002-0205-1165)
Isabella Scandurra PhD[4] (ORCID: 0000-0002-2597-1079)
Jonas Moll PhD[4] (ORCID: 0000-0002-4772-4730)
Åsa Cajander PhD[5] (ORCID: 0000-0001-7472-2215)
Hanife Rexhepi PhD[6] (ORCID: 0000-0001-8957-9853)
Maria Hägglund PhD[1,2] (ORCID: 0000-0002-6839-3651)

[1] Department of Women's and Children's Health, Uppsala University, Uppsala, Sweden
[2] Medtech Science & Innovation Centre, Uppsala University Hospital, Uppsala, Sweden
[3] Beth Israel Deaconess Medical Center, Harvard Medical School, Boston, MA, USA
[4] School of Business, Örebro University, Örebro, Sweden
[5] Department of Information Technology, Uppsala University, Uppsala, Sweden
[6] School of Informatics, University of Skövde, Skövde, Sweden

Disclaimers: The views expressed in the submitted article are his or her own and not an official position of the institution or funder.

Source(s) of support: This work was supported by NordForsk (grant number #100477) and the Swedish Research Council for Health, Working Life and Welfare (FORTE) (grant number #2020-01229).

Word count: 2515

Number of figures: 7

Number of tables: 2

Key words: adolescents; adolescent health; Patient Accessible Electronic Health Record (PAEHR); Electronic Health Record (EHR); patient portal; national survey; ehealth

References: 39

Disclosure of relationships and activities: There are no conflicts of interest to declare.

Confidential: For Review Only

ABSTRACT

Background: Having online access to electronic health records (EHRs) may help patients become engaged in their care at an early age. However, little is known about adolescents using patient portals. A national survey conducted within the Nordic eHealth project NORDeHEALTH provided an important opportunity to advance our understanding of adolescent users of patient portals. The present study explored reasons for reading the EHRs, the perceived usefulness of information and functions in a patient portal, and the association between frequency of use and encouragement to read the EHR.
Methods: Data were collected in a survey using convenience sampling, available through the Swedish online health portal during three weeks in January and February 2022. This study included a subset of items and only respondents aged 15-19. Demographic factors and frequencies on Likert-style questions were reported with descriptive statistics, while Fisher's exact test was used to explore differences in use frequency based on having been encouraged to read by a HCP.
Results: Of 13 008 users who completed the survey, 218 (1.7%) were unique users aged 15-19 (females: 77.1%). One fifth (47/218, 21.6%) had been encouraged by HCPs to read their records, and having been encouraged by HCPs was related to higher use frequency (p=.018). All types of information were rated high on usefulness, while some functions were rated low, such as blocking specific clinical notes from HCPs and managing services for family members. The main reason for reading their health records online was out of curiosity.
Conclusions: Adolescents who read their records online perceive it to be useful. Encouragement by HCPs can lead to increased use of patient portals among adolescents. Findings should be considered in the future design of patient portals for adolescents.

Keywords: adolescents; adolescent health; Patient Accessible Electronic Health Record (PAEHR); Electronic Health Record (EHR); patient portal; national survey; ehealth

KEY MESSAGES

What is already known on this topic

- Implementation of online record access for adolescents is controversial
- Citizens in Sweden have had online record access via a national patient portal for a decade
- Prior research has focused on adolescents without experience of using a patient portal, while existing studies of adolescent patient portal users have been limited to contexts of serious illness.
- Adolescents report similar benefits of reading EHRs to those of adults, such as reduced anxiety, enhanced knowledge about their illness, ability to ask informed questions and reflecting more on their health

What this study adds

- Encouragement by HCPs was related to higher use frequency. However, adolescents are rarely encouraged by HCPs to read their notes online.
- Adolescents reading their records perceive all types of information in the EHR as useful.

How this study might affect research, practice or policy

- Enhanced encouragement and support for adolescents will be required to support the transition into adulthood and involve adolescents in their own care.
- EHR vendors designing patient portals and patient portals intended for adolescent use in particular may benefit from considering the types of information and functions that adolescents find useful.

Confidential: For Review Only

INTRODUCTION

An increasing number of healthcare providers are giving patients access to their electronic health records (EHR), including patient information such as clinical notes, diagnoses, and medication lists. The practice of enabling access to free-text notes written in the EHRs by healthcare professionals (HCPs) is sometimes called 'open notes'.[1] Commonly, patients gain online record access (ORA) via secure online portals. Patient-accessible electronic health records (PAEHR) are becoming increasingly expected among patients, even affecting patients' choice of healthcare provider. In particular, adolescents are growing up with expectations of digital convenience that also applies to healthcare.[2]

During early childhood, PAEHR portal systems can often allow for parental proxy access, where parents or guardians of the minor have access to the EHR of the child. However, adolescents needing healthcare face a hidden battle in balancing privacy and personal autonomy with the need for support from parents. Worries about sensitive health information (e.g. mental health issues and sexual health) being exposed to parents through the PAEHR can dissuade young people from accessing medical care.[3-4] The transition from adolescent to adult patienthood has therefore raised concerns among HCPs[5–7] and implementing online access to EHRs is especially controversial in this population.

The e-service for PAEHRs in Sweden is *Journalen*, available via the national web patient portal 1177.se, accessible to patients in all 21 Swedish regions.[8] According to publicly available statistics,[9] *Journalen* is being accessed by a steadily increasing number of users; approximately 1,360,000 unique users per month during 2022. Adult parent users have automatic access to their child's records in *Journalen*, but lose this access when the child turns 13, and adolescents gain their record access at 16. When an adolescent is between 13 and 16 years old, parents and adolescents can apply for extended access outside of the default. Adolescent patients access the same information as adults. The content includes for example a list of prescribed medications, lab results, growth curves, vaccinations, diagnoses, maternity care records, referrals, warnings, and treatment plans (Figure 1). However, availability differs according to region and connected health care providers (who have agreed to give access) (Figure 2). For example, psychiatric notes are shown in 17 of 21 regions.[10] Functions such as prescription renewal and appointment booking are conducted on the patient portal, yet externally from the PAEHR.

Figure 1. The patient-accessible electronic health record *Journalen* after log-in (manually translated), showing the functions and information available.[11] Service produced by Inera AB under the auspices of Swedish municipalities and regions.

Figure 2. The information shown in *Journalen* depending on region or health care provider (green squares) during the time of the survey.

Two literature reviews on pediatric patient portals found limited studies in this age group. One reviewed definition and utilization[12] while the other focused on use, views, and experiences among adolescents.[13] While expressing a lack of knowledge of how to use PAEHRs,[2,14] adolescents have stated a desire to receive such information from HCPs.[2,15] In a US study examining EHR access of adolescents' records by accounts divided into self, surrogate, and delegate, there was a significant increase of use during adolescence.[16] Furthermore, HCPs have stated the benefits of guiding adolescent patients to become more involved and activated in their care.[17-18] Research has mostly been observational or focused on adolescents' attitudes towards patient portals, rather than experiences. In observational studies, adolescents demonstrate the use of the same portal features as adults,[19] including lab results,[16,20-21] appointment review,[16,20-21] messaging,[16,20] and medications.[21] Existing studies of adolescent users have been limited to contexts of serious illness, and these adolescents report similar benefits of reading EHRs to those of adults, such as reduced anxiety, enhanced knowledge about their illness, ability to ask informed questions and reflecting more on their health,[22] and empowerment.[5,22] A benefit unique for adolescents concerned a supported transition into adulthood.[22] To date, no studies have systematically examined how adolescents more broadly experience ORA.

Confidential: For Review Only

Study Aim

A recent scoping review concluded that with few exceptions (Australia, Canada, New Zealand, and the United Kingdom), the entire body of literature on adolescent access to EHRs had been carried out in the United States.[13] The present research explored reasons for reading the EHRs, the perceived usefulness of information and functions in a patient portal, and the association between frequency of use and HCP encouragement to read the EHR, among adolescents in Sweden.

METHODS

Study Design

Within the NORDeHEALTH research project,[23] an open survey designed to elicit the opinions and experiences of PAEHR users was conducted. The data were collected for three weeks from January to February 2022. Convenience sampling was used. Participants were notified about the survey when they logged into their records through the Swedish national PAEHR *Journalen*, by receiving a request to voluntarily participate in the survey, along with detailed information about the study. Accessing the survey without logging in was impossible. The participation request did not appear once the patient had completed the survey. Participants provided electronic consent and there was no financial incentive. Based on the definition of adolescence proposed by the United Nations (UN), youths aged 10-19 [24], only survey respondents aged 19 and younger were included for this paper. Due to an ethical-legal requirement of written parental consent for participants younger than 15 (The Act concerning the Ethical Review of Research Involving Humans[25]), which would complicate survey distribution, we opted to exclude those younger than 15 years. Therefore, the first survey item after consent concerned age and selecting *14 or younger* triggered information about exclusion and survey termination. The Checklist for Reporting Results of Internet E-Surveys (CHERRIES)[26] was used to report this study.

Data Collection

An anonymous questionnaire covering 83 items was designed in English and translated into Swedish. The questionnaire included a mixture of close- and open-ended questions with various response options (yes/no, multiple choice, and free-text form). The design and selection of items were informed by prior studies.[1,19,27] Answering close-ended questions was mandatory, while free-text questions were optional. The Swedish survey was distributed over 12 pages and some items were conditionally displayed based on responses to other items. Including conditional questions, the number of items ranged between 1-10 item per page. Respondents could move back and forth between survey sections, however did not have an option to review the filled form before submission. For this study, five items were included (see Figure 3) based on the study's aim. Three of these items used a 5-point Likert scale (ranging from disagree to agree), and two were close-ended questions. Data on use frequency was collected to enable comparison across groups. The items used in the current study are found in Figure 3, and answer options and item number can be found in Appendix A (English original and Swedish translation). The full survey and further details about the data collection procedure are found in a separate publication.[28] It should be noted that the availability of information and functions in *Journalen* varies across regions in Sweden, which may affect respondents' assessment. However, the items on perceived usefulness are formulated anticipatory (see Figure 3).

Figure 3. Survey items included in the study.

Statistical Analysis

Only completed questionnaires were analyzed. Aside from using descriptive statistics for calculating percentages for different response options, the Fisher's exact test was used to explore the difference in use frequency based on whether a HCP (physician, nurse, psychologist, physiotherapist, or other medical staff) had encouraged PAEHR use. Non-parametric tests were conducted due to the small sample size, and the significance level was set to 95%. The use frequency response options *This is my*

Confidential: For Review Only

first time and *2-9 times* as well as *10-20 times* and *More than 20 times* were merged for analysis. The SPSS 28 software was used for analyses, and figures were produced using R.

Patient and public involvement
The survey was tested with the public before launch to assess its clarity and response burden, leading to minor adjustments. There was no other patient and public involvement in the design or conduct of the study.

RESULTS

Characteristics of the Participants
During the study period, 23,855 users opened the survey, and 15,867 (66.5%) started filling it in. Of these, 13,010 (82.0%) submitted the survey. Two (2/13 010, 0.02%) were 14 years old or younger and were excluded from the survey. Of the 13,008 respondents who completed the survey, 218 were 15-19 years old (1.7%). Of respondents aged between 15-19, 77.1% (168/218) identified as women, 17.9% (39/218) as men while 5.0% (11/218) selected Other. See data on survey completion in a previous publication[28] and descriptive statistics for all variables in Appendix B.

Experience with Accessing Records Online
Two out of five respondents had logged in more than 20 times (85/218, 39.0%) and one third had logged in 2-9 times (74/218, 33.9%) (see Table 1).

Table 1. Participants' frequency of accessing their health records online.

Frequency of health record access, n (%)	Participants (N=218)
This is my first time	11 (5)
2 to 9 times	74 (33.9)
10-20 times	48 (22)
More than 20 times	85 (39)

Encouragement to Read the Health Records Online
One survey question focused on whether respondents had been encouraged or reminded by anyone or any media source to read their records online. The most common response was *nobody encouraged me* (118/218, 54.1%), see Figure 4. Of four respondents that chose *Other*, one respondent provided a free-text description, citing "myself". Among the 47 individuals (47/218, 21.6%) who had been encouraged by some type of HCP, 36 (76.6%) were frequent users of the patient portal, which is higher than those who had not been encouraged (97/171, 56.7%) (p=.018).

Figure 4. Encouragement to read health records online. Because respondents could select multiple items, the total number can exceed 100%. Legend: *Types of HCPs.

Reasons for Reading the Health Records Online
Four reasons were chosen by more than half of respondents: *out of general curiosity* (170/218, 78.0%), *to get an overview of one's medical history and/or treatment* (159/218, 72.9%), *to ensure having understood what the physician or nurse said* (149/218, 68.3%), and *to improve one's understanding for one's health issue* (138/218, 63.3%) (see Figure 5). Of 50 respondents who selected *Other*, 16 provided free-text comments. The comments included ensuring accuracy, memory reinforcement, poor notification frequency (i.e. logging in to check for new information due to lack of notifications), specification of sought information (e.g., test results, Covid-19 vaccinations), and retrieving a record copy for monetary activity compensation.

Figure 5. Adolescents' reasons for reading health records online.

Usefulness of Portal Information and Function
All information types were considered useful by more than 3/4 of participants, see Figure 6.

Confidential: For Review Only

Figure 6. Adolescents' perceived usefulness of different types of patient portal information.

Perceived usefulness of various functions was also examined, most of which do not currently exist in *Journalen*. Most functions were perceived as useful by a majority of respondents, see Figure 7. Yet, four functions were considered useful by less than half of respondents: *accessing information and managing services for children* and for *family members*, *contributing information about expectations for the health care visit*, and *blocking specific clinical notes from certain HCPs/medical staff*. Of 24 participants who wrote a comment under *Other*, 7 referred to a specific function. Comments concerned the ability to view radiology images (median=4.5), diagnosis (median=3.5), dentistry records (median=5), ability to read children's records when under 18 (median=5), records from childhood (median=5), and to view appointments from gynecologic care (median=5). Other free-text answers were non-responses or unspecified. *Medical certificates and other certificates* concerned certificates from HCPs, such as COVID-19 vaccination and for insurance and sick leave.

Figure 7. Adolescents' perceived usefulness of patient portal functions.

DISCUSSION

In our national survey of PAEHR experiences in Sweden, adolescents perceived all proposed clinical information as consistently useful. Ratings for portal functions varied. Common reasons for reading records were curiosity and to get an overview of medical history. Sharing clinical information was the least common reason for accessing the record. Encouragement by HCPs to read records was associated with higher use. The reasons for reading records align with previous research on adults[19] and US adolescents.[22]

While all information types were rated highly on usefulness, test results were considered the most useful. This result accords with earlier research on adolescents,[16,20-21] but also with research on parents[29-30] and general adult populations.[19] Unsurprisingly, adolescents rated the ability to manage family members or children low. The function perceived as the third most useful was the ability to order and manage various certificates. This was likely associated with the timing of survey distribution, as the ongoing COVID-19 pandemic required Swedish citizens to present COVID-19 certificates for traveling and attending events. The boost of PAEHR use as a result of the COVID-19 pandemic has been mentioned previously.[31] Related to this, it is somewhat unexpected that immunizations were rated among the least useful types of information. However, Swedish regions differed in managing COVID-19 vaccination through the national e-service or other apps, and not all regions displayed information on vaccinations. Another finding was that adolescents do not read their PAEHRs to share information. This finding is consistent with a previous survey study based in Canada, where adolescents without access to their records anticipated not being likely to share information from the records with others.[32]

Encouragement from HCPs was linked to higher use, indicating the influence of social factors in eHealth adoption.[33] Another possible explanation is that those with more active medical issues needed to review their health record more frequently. Encouragement from HCPs and family/friends has increased PAEHR use in adults,[34] suggesting that this may be an important factor influencing adoption and use also in adolescents. Lack of HCP encouragement aligns with research on adults in the United States[35] and Sweden,[36] and may stem from concerns about medical literacy, increased workload, and uncertainty about visible information.[2,5,37–39] Not encouraging adolescents may lead to a missed opportunity for early patient involvement.[17] Increased PAEHR usage among adolescents can motivate policy-making stakeholders and EHR vendors to improve patient portals for this population.[5]

This study has limitations. Response biases in self-report surveys are unknown. Survey availability on the national patient portal may have influenced respondents' decisions to respond based on their competence and skills in using the service, potentially limiting the generalizability of the findings and causing positive bias as most adolescent respondents were frequent users. However, we opted to use convenience sampling as part of an exploratory approach. The majority of participants were women, which is consistent with adult and previous adolescent PAEHR users but represents a

Confidential: For Review Only

strong gender imbalance. Only 1.7% of the total respondents were aged 15-19, possibly due to younger individuals being less affected by health conditions and less engaged in personal health management. The survey was also designed for adults, and adolescents may have struggled to understand some questions. Furthermore, the reliability and validity of the survey utilized in this study have not been formally evaluated. While the survey was designed to capture the intended data, a comprehensive assessment of its psychometric properties was beyond the scope of this research. Still, the survey was tested with the public, as stated under Patient and public involvement.

The present results provide a broader understanding of adolescents reading their EHRs, however further work is required to establish the viability of the findings in other settings. Several questions remain, relating to for example eHealth literacy and confidentiality. It should be noted that the current study included only a subset of items from a larger survey, and there is an intention to publish additional findings on the adolescent population, on participants' views about security and privacy. Regarding our research methodology, we offer a few suggestions for future work. First, frequency of use was defined by how many times (first time, 2-9 times etc.) the users accessed their records in the past 12 months. Another approach is to ask how often users access their records (daily, weekly, etc.), which allows for an understanding of the visit regularity. Second, further research may benefit from providing a clearer distinction between a specific PAEHR service and patients' ORA more broadly. Lastly, including a larger and more evenly gender-distributed sample and specific patient groups with chronic diseases could furnish greater insights into adolescent users' experiences.

Data availability statement

Data are available from the corresponding author on reasonable request.

Ethics statements

Patient consent for publication

Electronic consent obtained from survey respondents.

Ethics approval

Ethical approval for this study was obtained from the Regional Ethical Review Board in Uppsala, Sweden (EPN 2021/05229). All respondents received study information and provided informed consent.

Abbreviations

EHR: electronic health record
HCP: healthcare professional
ORA: online record access
PAEHR: patient-accessible electronic health record

FOOTNOTES

Contributors: JH wrote the manuscript, created the tables, and designed the figures. MH, CB, IS, JM, ÅC, and HR contributed to the study design. All authors read the manuscript, provided feedback, and approved the final draft for submission.

Funding: This work was supported by NordForsk (grant number #100477) and the Swedish Research Council for Health, Working Life and Welfare (FORTE) (grant number #2020-01229).

Competing interests: None.

Patient and public involvement: Patients and/or the public were involved in survey testing but otherwise not involved in the design, or conduct, or reporting, or dissemination plans of this research.

Provenance and peer review: Not commissioned; externally peer reviewed.

Confidential: For Review Only

Figure captions:
Figure 1. The patient-accessible electronic health record *Journalen* after log-in (manually translated), showing the functions and information available.[11] Service produced by Inera AB under the auspices of Swedish municipalities and regions.
Figure 2. The information shown in *Journalen* depending on region or health care provider (green squares) during the time of the survey.
Figure 3. Survey items included in the study.
Figure 4. Encouragement to read health records online. Because respondents could select multiple items, the total number can exceed 100%. Legend: *Types of HCPs.
Figure 5. Adolescents' reasons for reading health records online.
Figure 6. Adolescents' perceived usefulness of different types of patient portal information.
Figure 7. Adolescents' perceived usefulness of patient portal functions.

Confidential: For Review Only

REFERENCES

1. Walker J, Leveille S, Bell S, et al. OpenNotes After 7 Years: Patient Experiences With Ongoing Access to Their Clinicians' Outpatient Visit Notes. J Med Internet Res. 2019;21(5):e13876. doi:10.2196/13876
2. Beaton L, Williams I, Sanci L. Exploring adolescent and clinician perspectives on Australia. Aust J Prim Health. Published online March 15, 2021. doi:10.1071/PY20169
3. Bayer R, Santelli J, Klitzman R. New Challenges for Electronic Health Records: Confidentiality and Access to Sensitive Health Information About Parents and Adolescents. JAMA. 2015;313(1):29. doi:10.1001/jama.2014.15391
4. Ford CA. Foregone Health Care Among Adolescents. JAMA. 1999;282(23):2227. doi:10.1001/jama.282.23.2227
5. Carlson JL, Goldstein R, Buhr T, Buhr N. Teenager, parent, and clinician perspectives on the electronic health record. Pediatrics. 2020;145(3). doi:10.1542/peds.2019-0193
6. Goldstein RL, Anoshiravani A, Svetaz MV, Carlson JL. Providers' Perspectives on Adolescent Confidentiality and the Electronic Health Record: A State of Transition. J Adolesc Health. 2020;66:296-300. doi:10.1016/j.jadohealth.2019.09.020
7. Stablein T, Loud KJ, DiCapua C, Anthony DL. The Catch to Confidentiality: The Use of Electronic Health Records in Adolescent Health Care. J Adolesc Health. 2018;62(5):577-582. doi:10.1016/j.jadohealth.2017.11.296
8. Hägglund M, Scandurra I. Patients' Online Access to Electronic Health Records: Current Status and Experiences from the Implementation in Sweden. MEDINFO 2017 Precis Healthc Inform. Published online 2017:723-727. doi:10.3233/978-1-61499-830-3-723
9. Statistik för Ineras tjänster [Statistics for Inera's services]. Inera AB. Accessed August 25, 2023. https://www.inera.se/tjanster/statistik-for-ineras-tjanster/
10. Bärkås A, Scandurra I, Rexhepi H, Blease C, Cajander Å, Hägglund M. Patients' Access to Their Psychiatric Notes: Current Policies and Practices in Sweden. Int J Environ Res Public Health. 2021;18(17):9140. doi:10.3390/ijerph18179140
11. Journalen. Inera. Published December 2022. Accessed December 8, 2022. https://journalen.1177.se/
12. Bush RA, Connelly CD, Fuller M, Pérez A. Implementation of the Integrated Electronic Patient Portal in the Pediatric Population: A Systematic Review. Telemed J E-Health Off J Am Telemed Assoc. 2016;22(2):144-152. doi:10.1089/tmj.2015.0033
13. Hagström J, Blease C, Haage B, Scandurra I, Hansson S, Hägglund M. Use, Views of and Experiences of Web-Based Access to Electronic Health Records for Children, Adolescents, and Parents: Scoping Review. J Med Internet Res. Published online 2022. doi:10.2196/40328
14. Miklin DJ, Vangara SS, Delamater AM, Goodman KW. Understanding of and Barriers to Electronic Health Record Patient Portal Access in a Culturally Diverse Pediatric Population. JMIR Med Inform. 2019;7(2):e11570. doi:10.2196/11570

15. Bergman DA, Brown NL, Wilson S. Teen Use of a Patient Portal: A Qualitative Study of Parent and Teen Attitudes. Perspect Health Inf Manag. 2008;5:13.
16. Steitz BD, Cronin RM, Davis SE, Yan E, Jackson GP. Long-term patterns of patient portal use for pediatric patients at an academic medical center. Appl Clin Inform. 2017;8(3):779-793. doi:10.4338/ACI-2017-01-RA-0005
17. Bourgeois FC, DesRoches CM, Bell SK. Ethical Challenges Raised by OpenNotes for Pediatric and Adolescent Patients. Pediatrics. 2018;141(6):e20172745. doi:10.1542/peds.2017-2745
18. Lam BD, Bourgeois F, DesRoches CM, Dong Z, Bell SK. Attitudes, experiences, and safety behaviours of adolescents and young adults who read visit notes: Opportunities to engage patients early in their care. Future Heal J. 2021;8(3):e585-e592. doi:10.7861/fhj.2021-0118
19. Moll J, Rexhepi H, Cajander Å, et al. Patients' Experiences of Accessing Their Electronic Health Records: National Patient Survey in Sweden. J Med Internet Res. 2018;20(11):e9492. doi:10.2196/jmir.9492
20. Calman N, Pfister HR, Lesnewski R, Hauser D, Shroff N. Electronic Access to Adolescents' Health Records: Legal, Policy, and Practice Implications. Vol 22.; 2014:11-14. https://www.aafp.org/fpm/2015/0300/p11.html
21. Thompson LA, Martinko T, Budd P, Mercado R, Schentrup AM. Meaningful use of a confidential adolescent patient portal. J Adolesc Health. 2016;58(2):134-140. doi:10.1016/j.jadohealth.2015.10.015
22. Hong MK, Wilcox L, Feustel C, Wasileski-Masker K, Olson TA, Simoneaux SF. Adolescent and Caregiver use of a Tethered Personal Health Record System. AMIA Annu Symp Proc AMIA Symp. 2016;2016:628-637.
23. Hägglund M. Nordic countries lead new initiative on patient access to EHRs. TheBMJopinion. Published May 18, 2021. Accessed January 16, 2022. https://blogs.bmj.com/bmj/2021/05/18/maria-hagglund-nordic-countries-lead-new-initiative-on-patient-access-to-ehrs/
24. "Definition of Youth." United Nations Department of Economic and Social Affairs (UNDESA).
25. Riksdagsförvaltningen. 2003:460.; 2003. Accessed August 3, 2022. https://www.riksdagen.se/sv/dokument-lagar/dokument/svensk-forfattningssamling/lag-2003460-om-etikprovning-av-forskning-som_sfs-2003-460
26. Eysenbach G. Improving the Quality of Web Surveys: The Checklist for Reporting Results of Internet E-Surveys (CHERRIES). J Med Internet Res. 2004;6(3):e34. doi:10.2196/jmir.6.3.e34
27. Zanaboni P, Kummervold PE, Sørensen T, Johansen MA. Patient use and experience with online access to electronic health records in Norway: Results from an online survey. J Med Internet Res. 2020;22(2). doi:10.2196/16144
28. Hägglund M, Kharko A, Hagström J, et al. The NORDeHEALTH 2022 Patient Survey: A cross-sectional survey of national patient portal users in Norway, Sweden, Finland, and Estonia. J Med Internet Res. 2023;25:e47573. doi:10.2196/47573
29. Ahlers-Schmidt CR, Nguyen M. Parent Intention to Use a Patient Portal as Related to Their Children Following a Facilitated Demonstration. Telemed E-Health. 2013;19(12):979-981. doi:10.1089/tmj.2013.0041
30. Thompson LA, Black EW, Saliba H, Schentrup AM. Parents' knowledge of and opinions about healthcare laws and technology in primary care. Inform Prim Care. 2012;20(1):69-74. doi:10.14236/jhi.v20i1.49
31. Blease C, Salmi L, Hägglund M, Wachenheim D, DesRoches C. COVID-19 and Open Notes: A New Method to Enhance Patient Safety and Trust. JMIR Ment Health. 2021;8(6):e29314. doi:10.2196/29314
32. Frazer C, Ratchford F, Roch J. Enabling Adolescent Electronic Access to Personal Health Information. Stud Health Technol Inform. 2017;234:115-119.
33. Venkatesh V, Morris MG, Davis GB, Davis FD. User Acceptance of Information Technology: Toward a Unified View. MIS Q. 2003;27(3):425-478. doi:10.2307/30036540

Confidential: For Review Only

34. Mukhopadhyay S, Basak R, Carpenter D, Reithel BJ. Patient use of online medical records: an application of technology acceptance framework. Inf Comput Secur. 2019;28(1):97-115. doi:10.1108/ICS-07-2019-0076
35. DesRoches CM, Leveille S, Bell SK, et al. The Views and Experiences of Clinicians Sharing Medical Record Notes With Patients. JAMA Netw Open. 2020;3(3):e201753. doi:10.1001/jamanetworkopen.2020.1753
36. Rexhepi H, Moll J, Huvila I. Online electronic healthcare records: Comparing the views of cancer patients and others. Health Informatics J. 2020;26(4):2915-2929. doi:10.1177/1460458220944727
37. Hagström J, Blease C, Haage B, Scandurra I, Hansson S, Hägglund M. Views, Use, and Experiences of Web-Based Access to Pediatric Electronic Health Records for Children, Adolescents, and Parents: Scoping Review. J Med Internet Res. 2022;24(11):e40328. doi:10.2196/40328
38. Grünloh C, Myreteg G, Cajander Å, Rexhepi H. “Why Do They Need to Check Me?” Patient Participation Through eHealth and the Doctor-Patient Relationship: Qualitative Study. J Med Internet Res. 2018;20(1):e8444. doi:10.2196/JMIR.8444
39. Petersson J, Backman C. Patient-accessible online health records: Reconfigurations of clinical rhythms and doctors’ front- and backstage spaces. Soc Sci Med. 2022;292:114635. doi:10.1016/j.socscimed.2021.114635

Confidential: For Review Only

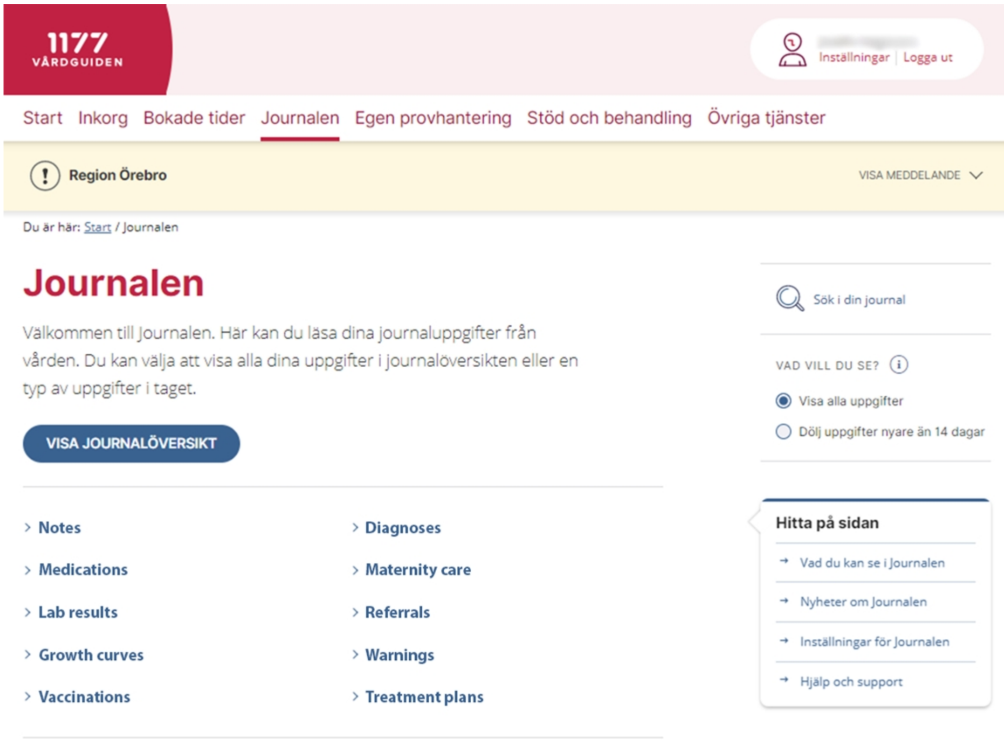

Figure 1. The patient-accessible electronic health record Journalen after log-in (manually translated), showing the functions and information available.[11] Service produced by Inera AB under the auspices of Swedish county councils and regions.

578x427mm (300 x 300 DPI)

	Medical Notes	Diagnoses	Lab results	Medications	Immunizations	Referrals	Medical alerts	Access Log Lists	Forms	Blocking of Record	Health contacts	Maternity care	Growth	Care plan	Total
Jönköping															11
Uppsala															11
Kronoberg															10
Värmland															10
Dalarna															9
Jämtland/ Härjedalen															9
Kalmar															9
Södermanland															9
Östergötland															9
Blekinge															8
Norrbotten															8
Västernorrland															8
Västmanland															8
Västra Götaland															8
Örebro															8
Gävleborg															7
Gotland															6
Halland															6
Stockholm															6
Västerbotten															6
Skåne															5
Capio (private care provider)															5
Total	22	20	21	15	18	12	13	5	1	20	21	4	3	1	

Figure 2. The information shown in Journalen depending on region or health care provider (green squares) during the time of the survey.

758x320mm (300 x 300 DPI)

1. How often do you think that you have accessed your health record during the last 12 months?
2. Did any of the following encourage or remind you to read your health record? (check all that apply)
3. Please indicate how much you disagree or agree with the following statement: I read my health record online… [reasons for reading]
4. How useful would it be to have access to the following information in the portal?
5. How useful would it be to have access to the following functions in the portal?

Figure 3. Survey questions included in the study.

520x176mm (300 x 300 DPI)

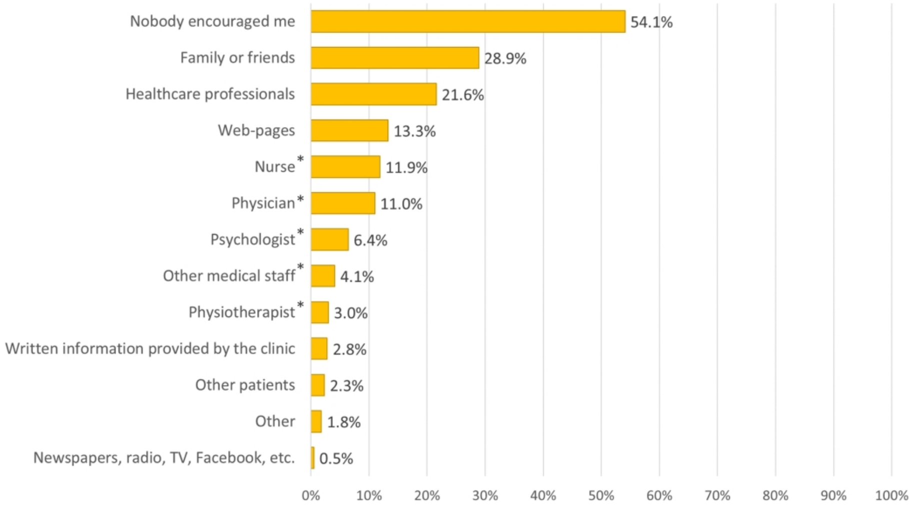

Figure 4. Encouragement to read health records online. Because respondents could select yes for multiple items, the total number can exceed 100%. Legend: *Types of HCPs.

478x250mm (300 x 300 DPI)

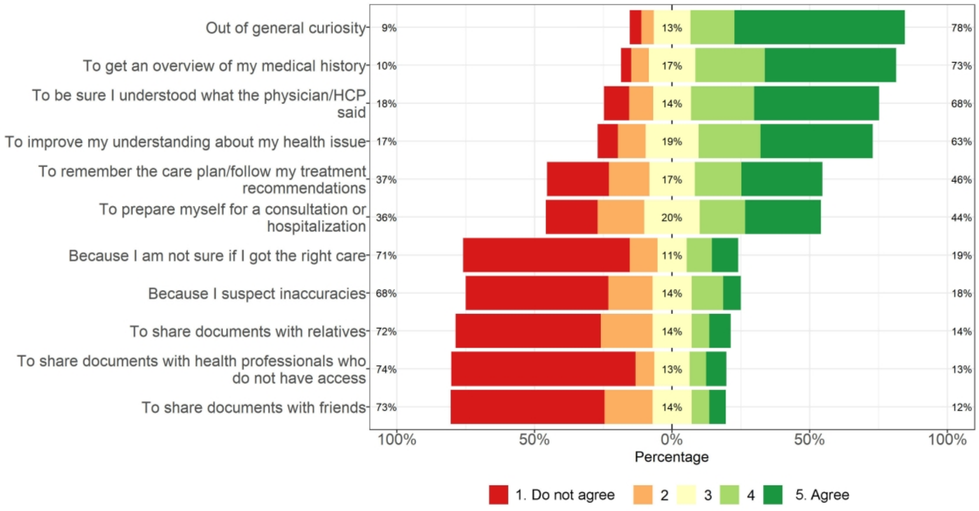

Figure 5. Adolescents' reasons for reading health records online.

292x152mm (300 x 300 DPI)

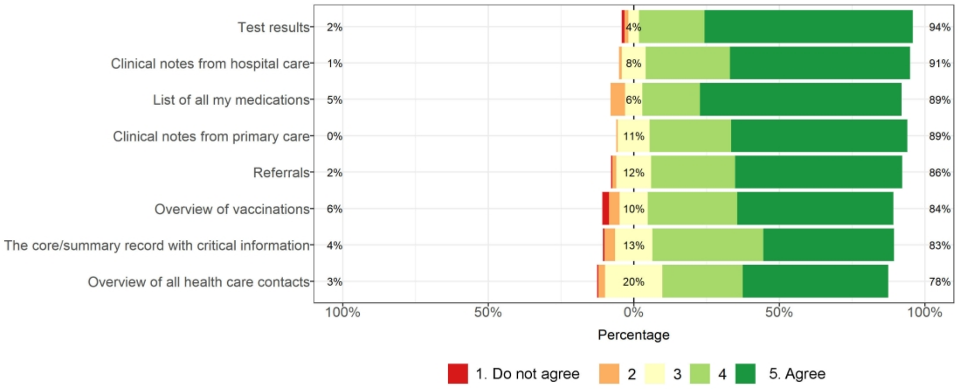

Figure 6. Adolescents' perceived usefulness of different types of patient portal information.

279x112mm (300 x 300 DPI)

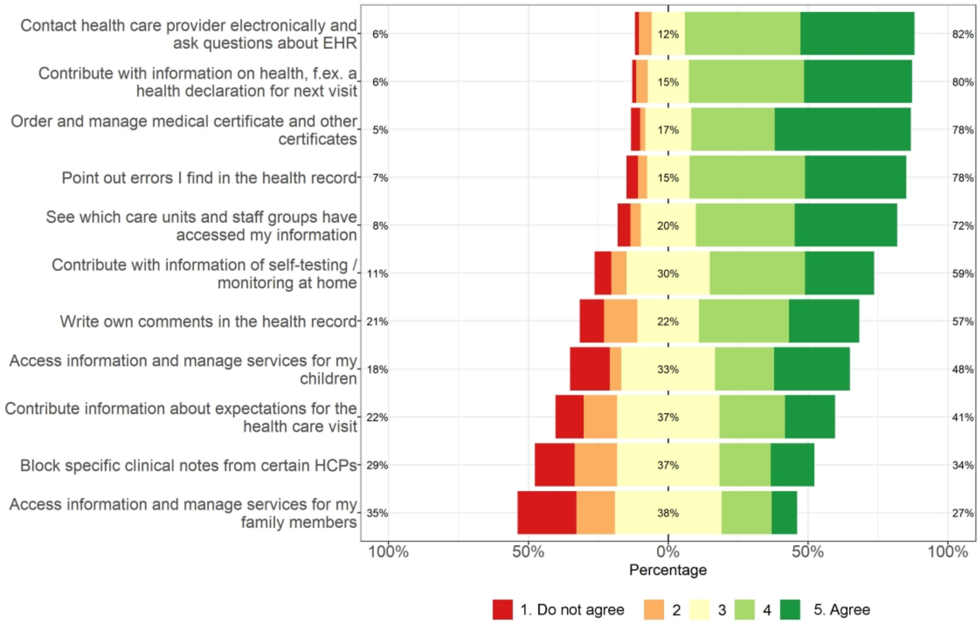

Figure 7. Adolescents' perceived usefulness of patient portal functions. Legend: *Functions that exist in all or some regions in Sweden. Only adult users over 18 years old have access to their children's records.

279x177mm (300 x 300 DPI)

Appendix A

Table 1. Original survey created in English

Number in survey	Question	Response alternatives
6	**How often do you think that you have accessed your health record during the last 12 months?**	1 - This is my first time<br>2 - 2 to 9 times<br>3 - 10 to 20 times<br>4 - More than 20 times
7	**Did any of the following encourage or remind you to read your health record?**	HCPs: Physician<br>HCPs: Nurse<br>HCPs: Psychologist<br>HCPs: Physiotherapist<br>HCPs: Other medical staff<br>Written information provided by the hospital/healthcare facility /clinic<br>Family or friends<br>Web-pages, such as the National health portal web-page, etc.<br>Newspapers, radio, TV, Facebook, etc.<br>Other patients<br>Nobody encouraged me<br>Other
8	Please indicate how much you disagree or agree with the following statement:<br>**I read my health record online…**	1 - Disagree,<br>2, 3, 4,<br>5 - Agree
	out of general curiosity	
	to improve my understanding about my health issue	
	to prepare myself for a consultation or hospitalization	
	to get an overview of my medical history and/or treatment	
	to be sure I understood what the physician/healthcare professional said	
	to remember the care plan/follow my treatment recommendations	
	because I suspect inaccuracies	
	to share documents with relatives	
	to share documents with friends	
	to share documents with health professionals who do not have access	
	because I am not sure if I got the right care	
	Other, please specify	

38	**How useful would it be to have access to the following information in the portal?**		1 - Not useful at all<br>2 - Not very useful<br>3 - Neither useful nor useless<br>4 - Useful<br>5 - Very useful
		The core/summary record with critical information	
		Referrals (content and how it is handled in care)	
		List of all pharmaceuticals/my medications	
		Overview of vaccinations	
		Results of tests	
		Clinical notes from primary care	
		Clinical notes from hospital care	
		Overview of all health care contacts	
39	**How useful would it be to have access to the following functions in the portal?**		1 - Not useful at all<br>2 - Not very useful<br>3 - Neither useful nor useless<br>4 - Useful<br>5 - Very useful
		Ability to access information and manage services for my children	
		Ability to access information and manage services for my family members	
		See which care units and staff groups have accessed my information (see log data)	
		Ability to block certain medical records from access by other medical staff	
		Ability to contact health care provider electronically and ask questions about medical record	
		Ability to point out errors I find in the health record	
		Ability to write own comments to text in the health record	
		Contribute with information on health, for example by providing a health declaration for next visit	
		Contribute with information of self-testing/monitoring at home	
		Contribute information about expectations for the health care visit	
		Ability to order and manage medical certificate and other certificates	
		Other	

Table B2. Swedish translation of survey

Number in survey	Question	Response alternatives
6	**Hur ofta har du läst i din journal under de senaste 12 månaderna?**	1 - Det här är första gången<br>2 - 2-9 gånger<br>3 - 10-20 gånger<br>4 - Mer än 20 gånger
7	**Har du blivit uppmuntrad eller påmind att läsa din journal av någon/något av följande:**	Vårdpersonal: Läkare<br>Vårdpersonal: Sjuksköterska<br>Vårdpersonal: Psykolog<br>Vårdpersonal: Fysioterapeut/sjukgymnast<br>Vårdpersonal: Annan vårdpersonal<br>Skriftlig information från sjukhuset/vårdinrättning/klinik<br>Familj eller vänner<br>Webbsidor, såsom 1177.se Vårdguiden, etc.<br>Tidningar, radio, TV, Facebook, etc.<br>Andra patienter<br>Ingen har uppmuntrat eller påmint mig<br>Annat
8	Markera i vilken utsträckning du instämmer med följande påståenden:<br>**Jag läser min journal på internet…**	1 - Instämmer inte, 2, 3, 4,<br>5 - Instämmer
	Av nyfikenhet	
	För att förbättra min förståelse för mitt hälsotillstånd	
	För att förbereda mig själv för ett vårdbesök eller sjukhusvistelse	
	För att få en överblick av min hälsohistoria och/eller bakgrund	
	För att försäkra mig om att jag förstått vad läkaren/vårdpersonalen sagt	
	För att komma ihåg vårdplanen/följa mina behandlingsrekommendationer	
	För att jag misstänker felaktigheter	
	För att dela med mig av information till släkt	
	För att dela med mig av information till vänner	
	För att dela med mig av information till vårdpersonal som inte har tillgång till min journal	
	För att jag är osäker på om jag fått rätt vård	
	Annat, specificera gärna	

38	**Hur användbart skulle det vara för dig att ha tillgång till följande information i *Journalen*?**		1 - Inte alls användbart<br>2 - Inte särskilt användbart<br>3 - Varken eller<br>4 - Användbart<br>5 - Väldigt användbart
		En sammanfattning av journalen med viktig information	
		Remisser (innehåll och status)	
		Lista över mina läkemedel/mediciner	
		Vaccinationer	
		Provsvar	
		Journalanteckningar från primärvården	
		Journalanteckningar från specialistvården	
		Överblick av samtliga kontakter med vården	
39	**Hur användbart skulle det vara för dig att ha tillgång till följande funktioner i *Journalen*?**		1 - Inte alls användbart<br>2 - Inte särskilt användbart<br>3 - Varken eller<br>4 - Användbart<br>5 - Väldigt användbart
		Möjlighet att ta del av information och hantera tjänster för mina barn	
		Möjlighet att ta del av information och hantera tjänster för andra familjemedlemmar	
		Möjlighet att kunna blockera specifika journalanteckningar för viss vårdpersonal	
		Se vilka vårdinrättningar och personal som har läst i min journal (loggar)	
		Möjlighet att kunna kontakta vårdpersonal online för att ställa frågor om min journal	
		Möjlighet att påpeka felaktigheter i journalen	
		Möjlighet att skriva egna kommentarer i journalen	
		Möjlighet att bidra med information om min hälsa, t ex genom att fylla i en hälsodeklaration, inför ett besök	
		Möjlighet att bidra med information från självtest/monitorering hemma	
		Möjlighet att bidra med information om mina förväntningar på vårdbesöket	
		Möjlighet att beställa och hantera sjukskrivning, medicinsk intyg och andra dokument (vaccinationsintyg t ex)	
		Annat	

Appendix B

Detailed results from the survey questions chosen for this study.

Encouragement to Read.

Did any of the following encourage or remind you to read your health record? n (%)	Frequency
HCPs: Physician	24 (11.0)
HCPs: Nurse	26 (11.9)
HCPs: Psychologist	14 (6.4)
HCPs: Physiotherapist	6 (2.8)
HCPs: Other medical staff	9 (4.1)
Written information provided by the hospital/healthcare facility/clinic	12 (5.5)
Family or friends	63 (28.9)
Web-pages, such as the National health portal web-page, etc.	29 (13.3)
Newspapers, radio, TV, Facebook, etc.	1 (0.5)
Other patients	5 (2.3)
Nobody encouraged me	118 (54.1)
Other	4 (1.8)
**Encouraged by HCP or not, n (%)**	
Encouraged by HCP	47 (21.6)
Not encouraged by HCP	171 (78.4)

Reasons for reading health records online.

**I read my health record online…, n** (%)	1. Disagree	2	3	4	5. Agree
… out of general curiosity	9 (4.1)	10 (4.6)	29 (13.3)	35 (16.1)	135 (61.9)
… to improve my understanding about my health issue	16 (7.3)	22 (10.1)	42 (19.3)	49 (22.5)	89 (40.8)
… to prepare myself for a consultation or hospitalization	41 (18.8)	37 (17.0)	44 (20.2)	36 (16.5)	60 (27.5)
… to get an overview of my medical history and/or treatment	8 (3.7)	14 (6.4)	37 (17.0)	55 (25.2)	104 (47.7)
… to be sure I understood what the physician/healthcare professional said	20 (9.2)	19 (8.7)	30 (13.8)	50 (22.9)	99 (45.4)
… to remember the care plan/follow my treatment recommendations	49 (22.5)	32 (14.7)	36 (16.5)	37 (17.0)	64 (29.4)
… because I suspect inaccuracies	113 (51.8)	35 (16.1)	31 (14.2)	25 (11.5)	14 (6.4)
… to share documents with relatives	115 (52.8)	41 (18.8)	31 (14.2)	14 (6.4)	17 (7.8)
… to share documents with friends	122 (56.0)	38 (17.4)	31 (14.2)	14 (6.4)	13 (6.0)
… to share documents with healthcare professionals who do not have access	146 (67.0)	15 (6.9)	28 (12.8)	13 (6.0)	16 (7.3)
… because I am not sure if I got the right care	132 (60.6)	22 (10.1)	23 (10.6)	20 (9.2)	21 (9.6)
Other	168 (77.1)	20 (9.2)	5 (2.3)	3 (1.4)	22 (10.1)

Perceived usefulness of various types of information.

Information	Not useful at all	Not very useful	Neither useful nor useless	Useful	Very useful
The core/summary record with critical information	1 (0.5)	8 (3.7)	28 (12.8)	83 (38.1)	98 (45.0)
Referrals (content and how it is handled in care)	1 (0.5)	3 (1.4)	26 (11.9)	63 (28.9)	125 (11.9)
List of all pharmaceuticals / my medications	0 (0)	11 (5.0)	13 (6.0)	43 (19.7)	151 (69.3)
Overview of vaccinations	5 (2.3)	8 (3.7)	21 (9.6)	67 (30.7)	117 (53.7)
Results of tests (not available in all regions)	2 (0.9)	3 (1.4)	8 (3.7)	49 (22.5)	156 (71.6)
Clinical notes from primary care	0 (0)	1 (0.5)	24 (11.0)	61 (28.0)	132 (60.6)
Clinical notes from hospital care	0 (0)	2 (0.9)	18 (8.3)	63 (28.9)	135 (61.9)
Overview of all health care contacts	1 (0.5)	5 (2.3)	43 (19.7)	60 (27.5)	109 (50)

Perceived usefulness of various functions.

Function	Not useful at all	Not very useful	Neither useful nor useless	Useful	Very useful
Ability to access information and manage services for my children (not available in all regions).	31 (14.2)	9 (4.1)	73 (33.5)	46 (21.1)	59 (27.1)
Ability to access information and manage services for my family members.	46 (21.1)	30 (13.8)	83 (38.1)	39 (17.9)	20 (9.2)
Ability to block specific clinical notes from certain healthcare professionals/medical staff.	31 (14.2)	33 (15.1)	80 (36.7)	40 (18.3)	34 (15.6)
See which care units and staff groups have accessed my information (see log data).	10 (4.6)	8 (3.7)	43 (19.7)	77 (35.3)	80 (36.7)
Ability to contact health care provider electronically and ask questions about medical record.	3 (1.4)	10 (4.6)	26 (11.9)	90 (41.3)	89 (40.8)
Ability to point out errors I find in the health record.	9 (4.1)	7 (3.2)	33 (15.1)	90 (41.3)	79 (36.2)
Ability to write own comments to text in the health record.	19 (8.7)	26 (11.9)	48 (22.0)	70 (32.1)	55 (25.2)
Contribute with information on health, for example by providing a health declaration for next visit.	3 (1.4)	9 (4.1)	32 (14.7)	90 (41.3)	84 (38.5)
Contribute with information of self-testing/monitoring at home.	13 (6.0)	12 (5.5)	65 (29.8)	74 (33.9)	54 (24.8)
Contribute information about expectations for the health care visit.	22 (10.1)	26 (11.9)	80 (36.7)	51 (23.4)	39 (17.9)
Ability to order and manage medical certificate and other certificates.	7 (3.2)	4 (1.8)	36 (16.5)	65 (29.8)	106 (48.6)
